# Synaptic circuitry of identified neurons in the antennal lobe of *Drosophila melanogaster*

**DOI:** 10.1002/cne.23966

**Published:** 2016-03-09

**Authors:** Jürgen Rybak, Giovanni Talarico, Santiago Ruiz, Christopher Arnold, Rafael Cantera, Bill S. Hansson

**Affiliations:** ^1^ Department of Evolutionary Neuroethology Max Planck Institute for Chemical Ecology 07745 Jena Germany; ^2^ Clemente Estable Institute of Biological Research 11600 Montevideo Uruguay; ^3^ Zoology Department Stockholm University 10691 Stockholm Sweden

**Keywords:** Drosophila *melanogaster*, olfactory system, glomerulus, projection neuron, ultrastructure, synaptic microcircuits

## Abstract

In *Drosophila melanogaster* olfactory sensory neurons (OSNs) establish synapses with projection neurons (PNs) and local interneurons within antennal lobe (AL) glomeruli. Substantial knowledge regarding this circuitry has been obtained by functional studies, whereas ultrastructural evidence of synaptic contacts is scarce. To fill this gap, we studied serial sections of three glomeruli using electron microscopy. Ectopic expression of a membrane‐bound peroxidase allowed us to map synaptic sites along PN dendrites. Our data prove for the first time that each of the three major types of AL neurons is both pre‐ and postsynaptic to the other two types, as previously indicated by functional studies. PN dendrites carry a large proportion of output synapses, with approximately one output per every three input synapses. Detailed reconstructions of PN dendrites showed that these synapses are distributed unevenly, with input and output sites partially segregated along a proximal–distal gradient and the thinnest branches carrying solely input synapses. Moreover, our data indicate synapse clustering, as we found evidence of dendritic tiling of PN dendrites. PN output synapses exhibited T‐shaped presynaptic densities, mostly arranged as tetrads. In contrast, output synapses from putative OSNs showed elongated presynaptic densities in which the T‐bar platform was supported by several pedestals and contacted as many as 20 postsynaptic profiles. We also discovered synaptic contacts between the putative OSNs. The average synaptic density in the glomerular neuropil was about two synapses/µm^3^. These results are discussed with regard to current models of olfactory glomerular microcircuits across species. J. Comp. Neurol. 524:1920–1956, 2016. © 2016 The Authors The Journal of Comparative Neurology Published by Wiley Periodicals, Inc.

**Abbreviations (Nomenclature after Ito et al., 2014)**
adPN: anterior–dorsal soma cluster of uniglomerular projection neurons
AMMC: antennal mechanosensory and motor center
AL: antennal lobe
ALH: antennal hub (nonglomerular neuropil of the antennal lobe)
ALT: antennal lobe tract
AN: antennal nerve
AN‐Me‐SN: antennal nerve mechanosensory sensory neurons
AN‐OSN: antennal nerve olfactory sensory neurons
ap‐cre: anterior–posterior tract of the crepine
ax: axon
c: connector
CRE: crepine of the inferior medial protocerebrum
DL5 DL5: glomerulus
DM2 DM2: glomerulus
ES: esophagus
gl: glia
GL: antennal lobe glomerulus
iALC: inferior antennal lobe commissure
lPN: lateral soma cluster of uniglomerular projection neurons
LH: lateral horn
LN: local interneuron
lp: lateral passage (bundle of LN and PN primary neurites)
lALT: lateral antennal lobe tract
mALT: medial antennal lobe tract
mlALT: mediolateral antennal lobe tract
mPN: multiglomerular projection neuron
MB: mushroom body
MB‐ML: medial lobe of the mushroom body
MV: microvolumen
N: nonlabeled (nonidentified), neuronal profile
NV: neurite volume
OSN: olfactory sensory neuron
PN: labeled projection neuron
pN: primary neurite
Pe: perineurium
PR: protocerebrum
sALC: superior antennal lobe commissure
so: soma(‐ta)
sy: synapse, synaptic
T: trachea
TEM: transmission electron microscopy
uPN: uniglomerular projection neuron
VA7: glomerulus VA7
V: glomerulus V
vPN: ventral soma cluster of uniglomerular projection neurons

The neural circuitry of the antennal lobe (AL) of the vinegar fly *Drosophila melanogaster* is one of the principal models for the study of how chemosensory information is processed in the brain. Thus it has been investigated intensively with a variety of anatomical, physiological, and genetic methods (Stocker, 1994; Vosshall and Stocker, 2007; Wilson, 2011). Very little is known, however, about the synaptic circuitry, synapse structure, numbers, and identity of pre‐ and postsynaptic components of this brain center.

The olfactory sensory neurons (OSNs) located in the antennae and maxillary palps send axons to the AL, where they form synapses with different types of interneurons within functional and morphological units called glomeruli (Stocker, 1994; Couto et al., 2005). These generic structural features of glomerular anatomy are preserved across species (Hildebrand and Shepherd, 1997; Ache and Young, 2005; Martin et al., 2011). Olfactory glomeruli are spherical structures of synaptic neuropil that can be morphologically recognized by size, shape, and location. Their number is specific for sex and species (Rospars, 1988), and their physiological identity results from selective innervation by OSNs that express different odorant receptors. It is thus possible to create both spatial and functional maps of the AL. Anatomical and physiological maps have been generated for *Drosophia melanogaster* (Stocker, 2001; Couto et al., 2005; Laissue and Vosshall, 2008; Knaden et al., 2012), the honeybee *Apis mellifera* (Galizia et al., 1999), and several moth species: the turnip moth *Agotis segetum* (Hansson et al., 1992), the tobacco budworm *Heliothis virescens* (Lofaldli et al., 2010), the silk moth *Bombyx mori* (Namiki et al., 2009), and the tobacco hawk moth *Manduca sexta* (el Jundi et al., 2009; Grosse‐Wilde et al., 2011).

Each of the 60 classes of OSNs projecting to discrete glomeruli in the vinegar fly (Vosshall and Stocker, 2007) form specific information channels, i.e., one‐to‐one connections to second‐order projection neurons (PNs) (for review, see Hong and Luo, 2014). Two classes of OSNs, expressing odorant receptors (ORs) and ionotropic receptors (IRs), respectively, form segregated pathways into olfactory subsystems of the AL (Silbering et al., 2011).

Two types of cell‐lineage–specific subsets of AL output neurons constitute the principal pathways from the AL to central brain regions, such as the mushroom bodies (MBs) and the lateral horn (LH). These output elements are uniglomerular projection neurons (uPNs), with axons running in the medial antennal lobe tract (mALT) and multiglomerular projections neurons (mPNs), with axons in the mediolateral AL tract (mlALT) and the mALT (Jefferis et al., 2001, 2007; Tanaka et al., 2012; Strutz et al., 2014; Wang et al., 2014).

Inter‐ and intraglomerular information is internally processed in the AL by local interneurons (LNs) that convey information between glomeruli (Stocker, 1994; Ignell et al., 2009; Nässel, 2009; Hu et al., 2010; Winther and Ignell, 2010). These modulatory, multiglomerular LNs interconnect the glomeruli, forming diverse subclasses of neurons with sparse and/or dense innervation of glomeruli (Okada et al., 2009; Tanaka et al., 2009; Chou et al., 2010; Seki et al., 2010). Local interneurons can be either inhibitory, containing γ‐aminobutyric acid (GABA) (Wilson and Laurent, 2005) or glutamate (Liu and Wilson, 2013), or excitatory, containing acetylcholine (Shang et al., 2007; Seki et al., 2010).

Synaptic activity in ensembles of glomeruli represent a spatiotemporal odor code that depends on the combinatorial pattern of glomerular activation (Galizia and Sachse, 2010). This code is the basis of behavior.

A certain relationship between the valence (attractive vs. aversive) of an odor stimulus and the topographic region of the AL where glomeruli are activated (Knaden et al., 2012) has been demonstrated in *D. melanogaster*. Processing in specific glomeruli can also be highly specialized for, e.g., CO_2_ (Suh et al., 2004; Sachse et al., 2007), geosmin (Kwon et al., 2006; Stensmyr et al., 2012), or pheromone information (Hansson et al., 1991; Agarwal and Isacoff, 2011; Grosjean et al., 2011), thereby forming part of a labeled line connectivity mediating ecologically relevant odor stimuli.

The glomeruli studied here were chosen to represent different behavioral or anatomical categories. DM2 is a glomerulus targeted by antennal ester‐specific OSNs mediating attraction in flies (de Bruyne et al., 2001; Stensmyr et al., 2003; Pelz et al., 2006). DL5 is innervated by antennal OSNs and is targeted by OSNs detecting odors of negative valence, such as benzaldehyde, whereas VA7 is targeted by unique OSNs from the palp. These neurons detect odors, such as 4‐methylphenol and 2‐methylphenol, of unknown function (de Bruyne et al., 1999).

## AL synaptic organization

The synaptic organization of the insect AL has been investigated in *Manduca sexta* (Sun et al., 1997; Lei et al., 2010), *Periplaneta americana* (Salecker and Distler, 1990; Malun, 1991a, 1991b; Distler et al., 1998), *Apis mellifera* (Gascuel and Masson, 1991; Brown et al., 2002), and *D. melanogaster* (Stocker et al., 1990; Tanaka et al., 2011). These studies revealed the existence of structural motifs and canonical circuits similar to those found in the olfactory bulb in vertebrates (Hildebrand and Shepherd, 1997; Chen and Shepherd, 2005). The most prominent feature is the OSN‐to‐PN synapse, where PNs receive strong, convergent sensory input via the OSN feed‐forward synapse (Boeckh and Tolbert, 1993; Stocker, 2001; Wilson, 2011). An interesting feature of the synaptic circuitry of the insect AL is the existence of reciprocal dendrodendritic synapses between PNs and LNs, providing further intra‐ and interglomerular communication (Boeckh and Tolbert, 1993; Sun et al., 1997; Ng et al., 2002) a feature also described in the olfactory bulb of mammals (Yuste and Tank, 1996; Didier et al., 2001; Ache and Young, 2005) but so far not demonstrated in flies. Spatial segregation of input and output synapses along PN fibers has been documented with electron microscopy in *Periplaneta americana* and *Manduca sexta* (Malun, 1991a; Sun et al., 1997; Lei et al., 2010), and indicated by optical imaging in *Drosophila* (Pech et al., 2015). In the honeybee, spatial segregation of sensory input has been macroscopically well described (Kelber et al., 2006; Nishino et al., 2009). In this species the glomeruli are divided into two compartments: the cap and the core, which are differentially innervated by diverse sets of local interneurons (Flanagan and Mercer, 1989b; Fonta et al., 1993; Abel et al., 2001; Meyer et al., 2013).

Anatomical studies thus indicate local, and complex dendritic processing in insect glomeruli, whereas behavioral and physiological studies have shown that certain types of behavior can be directly linked to the function of individually identifiable glomeruli (Suh et al., 2004; Ibba et al., 2010; Stensmyr et al., 2012; reviewed in Hansson and Stensmyr, 2011). Therefore, the question arises of whether the synaptic circuitry is specific and different in functionally distinct and segregated subzones within the AL. Although light microscopic evidence exists for spatial and function segregation of AL neurons (Hansson et al., 1991; Mosca and Luo, 2014), so far it is not known whether in *Drosophil*a a single glomerulus contains specific synaptic microcircuits at the ultrastructural level.

## Goal of the study

In contrast to the wealth of detailed scientific knowledge on different aspects of olfaction in *Drosophila*, our knowledge of the synaptic circuitry of olfactory centers is scarce. A deeper understanding of the function of olfactory circuits is not possible before information on types, numbers, and distribution of glomerular synapses is obtained, a task that requires the aid of the electron microscope. Here we used transgenic flies to label PNs with a membrane‐bound electron‐dense marker for transmission electron microscopy to identify synaptic sites at the electron microscope level. To identify specific glomeruli, we produced 3D models of the AL combining confocal and electron microscopy. Thus, we identified, counted, and mapped synaptic input and output sites along serially reconstructed PN branches in glomeruli VA7, DL5, and DM2. Using these methods we were able to show the segregation of input and output synapses at the dendrites of PNs and microcircuits that these PNs form with putative olfactory sensory and local interneurons.

## MATERIALS AND METHODS

### Fly strains

Flies were raised on standard *Drosophila* medium diet at 25 °C under 12:12‐hour light:dark cycles. Three‐day‐old male flies (Oregon R) of the following strains were used for transmission electron microscopy (TEM) experiments. One‐week‐old females were used for confocal laser scanning microscopy (CLSM). The transgenic lines used in this study were: GH146‐GAL4 (UAS‐GCaMP1.3; GH146→GAL4/CyO, as a driver specific for a subset of PNs [Stocker et al., 1997], kindly provided by Reinhard Stocker; RRID:BDSC_30026 as a specific driver for PNs [(Stocker et al., 1997]); UAS‐GCaMP3.0 (RRID:BDSC_32116 [Tian et al., 2009]); Np1227‐GAL4 as a specific driver for local interneurons (LN1) (Okada et al., 2009); UAS‐HRP::CD2 (P[w{+mC}=UAS‐CD2::hrp]2/CyO; Bloomington Stock Center) to express the enzyme horseradish peroxidase (HRP) to label the PNs for TEM tracing (Watts et al., 2004); and UAS‐GFP (UAS‐mCD8:GFP, kindly provided by Hiromu Tanimoto) to express green fluorescent protein (GFP) for the study of PNs with CLSM. OSN projection patterns were analyzed by using the genetically expressed markers Orco‐GAL4 (RRID:BDSC_26818; Larsson et al., 2004) and Or22a‐GAL4 (RRID:BDSC_9951; Vosshall et al., 2000; Fishilevich and Vosshall, 2005). This approach was expected to be informative about potential subzones in a given glomerulus.

### Immunohistochemistry

Immunostaining with an antibody specific for Bruchpilot (monoclonal mouse antibody nc82, Developmental Studies Hybridoma Bank [DSHB], Iowa City, IA; cat. no. nc82; RRID: AB_528108) was used for general neuropil staining. This antibody is an IgG produced by a hybridoma clone from a large library generated against *Drosophila* heads (Hofbauer et al., 2009). It recognizes two proteins of 190 and 170 kDa in western blots of homogenized *Drosophila* heads, and the loss‐of‐function in the bruchpilot gene causes loss of T‐bars at active zones (Wagh et al., 2006). An anti‐GFP serum was used to visualize glomerular structures in GFP‐expressing PNs in brains with genotype GH146‐GAL4, UAS‐GFP. Anti‐GFP is a rabbit polyclonal antiserum that was raised against GFP isolated directly from the jellyfish *Aequorea victoria* and purified by ion‐exchange to remove nonspecific immunoglobulins (Invitrogen, La Jolla, CA; cat. no. A11122; RRID: AB_221568). It was used at 1:1,000 dilution to enhance the GFP signal. Following incubation, the brains were washed for 60 minutes at room temperature (RT) and incubated in 1:500 goat anti‐rabbit Alexa Fluor 488 (A11008) for GFP immunostaining.

Brains with a Lucifer yellow–injected projection neuron (Seki et al., 2010) were fixed in 4% paraformaldehyde in phosphate‐buffered solution (PBS; 0.1 M, pH 7.4) for 30 minutes on ice. Then the brains were washed with PBS containing 0.2% Triton X‐100 (PBST) for 60 minutes (3 times for 20 minutes) at RT. After blocking in 5% normal goat serum (NGS) in PBST (PBST‐NGS) for 60 minutes at RT, the brains were coincubated with a 1:30 dilution of mouse nc82 antibody and 1:1,000 rabbit anti‐GFP in PBST for 2 days at 4 °C. Then the brains were washed for 60 minutes at RT, incubated in 1:200 goat anti‐mouse Alexa Fluor 633 (Invitrogen/Life Technologies, Eugene, OR; cat. no. A21070; RRID: AB_10562894), and coincubated with 1:500 goat anti‐rabbit Alexa Fluor 546 (Invitrogen/Life Technologies; cat. no. A11010; RRID: AB_143156) for 2 days at 4 °C. Afterwards, brains were washed again for 60 minutes and mounted in Vectashield (Vector, Burlingame, CA). For measurements of the AL neuropil and glomerular volumes, 1‐week‐old male specimens from wild‐type Canton S strains were dissected, processed as described above for nc82 staining, and incubated in anti‐mouse Alexa 488 (Invitrogen/ Life Technologies; cat. no. A11001; RRID: AB_10566289) as the secondary antibody.

### Confocal laser scanning microscopy

Dual confocal image stacks were generated with a Zeiss LSM510 META confocal microscope (Carl Zeiss, Jena, Germany), equipped with a 40 × water immersion objective (C‐Apochromat, NA: 1.2, Carl Zeiss) and a 63 × water immersion objective (C‐Apochromat, NA: 1.2, Carl Zeiss). For Lucifer yellow–stained cells, the argon–drypton 488‐nm laser line was used at a voxel resolution of approximately 1.5 × 1.5 × 3 μm. For immunohistochemistry, the 543‐nm line (detected with an emission spectrum of 550–620 nm) or the 633‐nm line (detected with an emission spectrum of 650–750 nm) of the helium–neon laser was used. Images were obtained at 0.45–46‐µm intervals with the 63 × objective and at 0.49–0.55‐m intervals with the 40 × objective at a resolution of 1,024 pixels. Images from the right AL were mirror‐imaged to match those from the left AL. Confocal images were adjusted for contrast and brightness by using Amira 5.3 (Fei, Visualization Science Group; RRID: RRID nif‐0000‐00262) and Adobe Photoshop CS (RRID: SciRes_000161, SCR_002078).

### In vitro 3D model of the antennal lobe

The mapping of neuronal structures was based on dual‐channel scans of brains doubly stained for synaptic neuropil (monoclonal antibody nc82) and GH146‐GAL4–driven expression in PNs. Glomeruli and anatomical structures of the AL were reconstructed and named according to Couto et al. (2005), Tanaka et al. (2012), and an AL atlas based on genetically labeled olfactory receptor neurons (OSNs) (Grabe et al., 2015). Interactive 3D pdf files were created following the strategy of Ruthensteiner and Hess (2008) and Rybak et al. (2010). The segmented structures were rendered as 3D surface models in Amira (Visage Imaging, San Diego, CA), or TrakEM2 (Cardona et al., 2012), exported as wavefront (.obj) files, and imported to Adobe 3D reviewer (Adobe 3D extension). All digitized anatomical structures, glomeruli, and anatomical landmark structures (tracts, somata cluster) were then indexed, converted to Adobe .u3D format, and imported to Acrobat Reader Pro. The resulting interactive model was used as a reference to identify glomeruli in the 3D TEM‐based model (Supplementary Figs. S1, S2). Additionally, image registration was done manually with the transform editor of Amira using landmark glomeruli and labels (i.e., neuropil border) as fiducially marks.

### Histochemistry for TEM

Specific neuronal types were recognized, at the TEM level, by implementing a simplified version of the method introduced by Watts et al. (2004). It is based on the use of the GAL4‐UAS method (Brand and Perrimon, 1993) to express a transgene encoding the enzyme HRP coupled to a transmembrane protein domain causing this enzyme to accumulate in the membrane of the neurons expressing the driver. The enzyme is detected with the aid of a histochemical reaction that produces an electron‐dense precipitate that is osmiophilic and results in a black staining of the membrane of the neurons expressing the transgene. The flies were anesthetized with nitric oxide (Sleeper TAS, INJECT + MATIC, Geneva, Switzerland) and decapitated with a sharp needle. After decapitation, the head was immediately dipped in 0.05% Triton X‐100 in 0.1 M Sørensen's phosphate buffer, pH 7.4, and transferred to a droplet of freshly prepared ice‐cooled 2.5% glutaraldehyde in 0.1 M Sørensen's phosphate buffer, pH 7.4 (Leitch and Laurent, 1996; here without 0.2 M sucrose) for brain dissection. With a fine forceps and a sharp needle or iris scissors, the proboscis was cut off and the back of the head opened to allow fast fixative penetration. After 5 minutes the brain was dissected out of the head case and fixed for 1.5 hours. Samples were rinsed 3 × 5 minutes in ice‐cooled 0.1 M Sørensen's phosphate buffer, pH 7.4, rinsed 1 × 5 minutes in 0.1 Tris buffer, and preincubated for 15 minutes in the dark at RT in a filtered 0.05% solution of 3,3′‐diaminobenzidine (DAB; Sigma Aldrich, Taufkirchen, Germany) in 0.1 M Tris buffer, pH 7.4. The histochemical reaction was started by adding 10 µl (up to 80 µl when needed) of 1% hydrogen peroxide in the dark at RT and was visually inspected every few minutes between 30 and 60 minutes, until staining of the antennal lobes was obtained. Then samples were rinsed 4 × 5 minutes in 0.1 M Tris buffer at room temperature and 2 × 5 minutes in distilled water, postfixed for 1 hour in an aqueous 1% solution of osmium tetroxide, and rinsed 3 × 5 minutes in distilled water. The samples were dehydrated in a gradual series of ethanol (25, 50, 70, 80, 90, 95, and 100%) and gradual series of resin (Durcupan ACM embedding kit, Fluka) in ethanol (1:3, 1:1, 3:1) and embedded in pure resin overnight. Finally, samples were placed in molds with pure resin for polymerization at 60 °C for 48 hours.

### Serial sectioning and transmission electron microscopy

Semithin sections (average thickness 150 nm) were cut along the coronal plane of the brain with a diamond knife (Histojumbo, Diatome, Hatfield, PA), mounted on microscope slides, stained with 1% boracic toluidine blue, and used to localize appropriate sites for ultrastructural analysis. Serial ultrathin sections (average thickness 50 nm) were cut with a diamond knife (Ultra 35 °, Diatome) on a Reichert‐Jung (Leica, Wetzlar, Germany) Ultracut E ultramicrotome, collected on single slot grids, and dried on 25% Pioloform BM18 support film. The ultrathin sections were studied without poststaining. Electron microscopy was performed on Zeiss EM 900 and CEM 902A as well as Jeol (Freising, Germany) JEM 1400 transmission electron microscopes. Images were acquired in different ways according to the instrumentation. Digital micrographs were obtained using either the Zeiss CEM 902A equipped with a FastScan‐F114B digital camera (Tietz Video and Image Processing Systems, Gauting, Germany) or for acquiring the series predominantly the Jeol JEM 1400 with a Gatan Orius SC 1000 CCD camera (Gatan, Munich, Germany). The resulting DRM raw files from the latter were converted into tif format. After resampling, they were imported to Amira (Visage Imaging), or TrakEM2, a plug‐in of open‐source ImageJ, Fiji (RRID: SciRes_000137, SCR_002285) (Cardona et al., 2012).

### 3D ultrastructural model of the antennal lobe

Low‐magnification TEM images were used to produce a 3D model of the AL (Supplementary Fig. S2). Panoramic views were collected for the entire AL on the right half of the brain starting at approximately 10 µm of its anterior surface. The resin block was cut from anterior to posterior, along an axis perpendicular to the anterior–posterior body axis with an approximate tilt of 10 degrees toward dorsal and a slight lateral tilting. The collection of panoramic views was started at the level of the DA2 glomerulus at approximately 5–10 µm from the anterior surface of the AL. Photos were either acquired digitally (see above) or made on TEM plates using Kodak EM film (Carestream® Kodak® EM film # 4489, Sigma) at the Zeiss EM 900 (courtesy of M. Westermann, EMZ, Jena, Germany), resulting in approximately one series of 40‐section series with a z‐resolution of approximately 1 µm. TEM plates were digitized with a scanner. After the images were imported to TrakEM software (RRID:nlx_151924, SCR_008954) and affine alignment of the sections, prominent anatomical structures (AL nerve, the antennal lobe tracts [ALTs], neuronal soma, neuropil, glomeruli, and tracheoles) were reconstructed. The borders of the glomeruli were recognized by incoming large fibers, partial glial wrapping, whirling fiber pattern, adjacent tracts, and other neuroanatomical features. The identity of individual glomeruli in the TEM reconstruction was aided by the use of the CLSM model (in vitro 3D AL model, Supplementary Fig. S1) – 3D models of the antennal lobe and the spatial distribution of synapses in identified glomeruli synaptic circuitry will be available online at http://www.ice.mpg.de/ext/suppl-rybak.html.

### Alignment of image stacks for high‐resolution EM

TEM photographs were sorted as z‐stacks, resampled in xy resolution, and aligned along the z‐axis using the Landmark Editor in TrakEM2. Image series were prepared at different lateral resolution. After each of the high‐resolution image stacks was aligned, two columns at TEM magnification 12,000 × and three columns at 20,000 × magnification were created using automated transformation algorithms, and the labeled PN profiles were subsequently segmented using the Segmentation Editor of TrakEM2 (Fiji). Using a sparse reconstruction technique, genetically labeled PN profiles containing blackened membranes were identified and traced in consecutive sections. Additional, nonlabeled profiles were also traced from dense reconstruction of synaptic networks in some regions.

Virtual objects for each profile (“area list”) were created using an ImageJ‐Fiji (http://fiji.sc/Fiji) plug‐in, TrakEM2 (http://www.ini.uzh.ch/∼acardona/trakem2.html). Synapses were marked with “ball” objects for pre‐ and postsynaptic parts of the synapse, and “connectors” were used to define synaptic configurations, i.e., the number of postsynaptic profiles opposing a presynaptic density, to document the number and location of postsynaptic sites for a given presynaptic element (see Fig. 8). To define morphometric features such as neuron shape (axiform for smooth, dendritiform for thin, and globular, or varicose‐bearing arborizations), terms were taken from Cardona et al. (2010b). For determining neurite length, branching order, and branching points of segmented profiles, data were exported from the 3D viewer of ImageJ‐Fiji either in .stl file format or as an Amira label field to Amira software for surface rendering using the Label Editor and SurfaceGen function of Amira. Digital data were then converted into a Skeleton graph by a simple threshold segmentation of the binary label file using the Autoskeleton function of Amira. Alternatively, these values were derived by the “treeline” construct of TrakEM2. Dendrograms of the PN profiles of the DL5 glomerulus were manually designed as a network diagram using E‐draw (www.edrawsoft.com/) and finally adjusted in Adobe Illustrator.

### Counting synaptic profiles in identified glomeruli

TEM digital micrographs were taken at magnifications of either 12,000 × and 15,000×, spanning the whole diameter of the glomeruli, or at 20,000 × to study regions of interest in the glomeruli. For some synapses additional images were made at higher magnification (50,000×). For each glomerulus (VA7, DL5, and DM2) a z‐series was done including 180 sections each approximately 50 nm thick and thus spanning 9 µm along the z‐axis, following predefined profiles in two or three subregions of the glomerulus. We performed both sparse reconstruction (on single‐labeled PN profiles) and dense reconstruction (including also unlabeled profiles) in all three glomeruli. All segmented profiles were named according to their cell type: projection neurons (PNs), putative olfactory sensory neurons (OSNs), and putative local interneurons (LNs), respectively, with an ascending number, e.g., PN1, PN2, LN1, LN2, and so on. Synaptic counts were given as the number of presynaptic sites (output synapse) and postsynaptic sites (input synapse), or both, for each neuron profile. The volumetric synaptic density is provided as synapses per neurite volume (NV), and synapses per neurite surface. The ratio was given by the number of synaptic output‐to‐input sites for a given neuron. The average of synapses along neuron arbors was given as synapse/µm for some selected profiles. Thus, synaptic measurement for all glomeruli are given as number, classified as either input or output synapses with regard to the labeled neuron profile in which the synapse was identified. The data were compiled in an Excel (Microsoft) table as the number of synaptic sites per volume (sy/µm^3^), per surface area (sy/µm^2^), and per length of neuron (sy/µm) (see Table 3, and Supplementary Figs. S3–S8).

Additionally, volumetric synaptic counts in defined subregions for all, i.e., labeled and nonlabeled profiles, were made in six microvolumes (MVs) in the center and the periphery of the glomeruli of interest. All series were coded and analyzed blind as to the glomerulus identity. Each MV measured 2 × 2 × 2 µm = 8 µm^3^, giving a total volume of 48 µm^3^ per glomerulus were studied in this way.

### Length analysis

Selected profiles and assembly of synaptic sites were reconstructed in TrakEM2 and exported as label to Amira 5.6 (Fei). The surface rendered constructs were resampled and smoothed, and then a centerline was determined that rendered the branching points and length of the neurite profiles.

### Nonlabeled profiles

In some cases, non‐HRP–labeled profiles were identified as putative LNs or OSNs according to their cellular features such as vesicle type, synaptic density, cellular shape, and location within the glomerulus (see Results). In the following, we refer to them as putative LN‐type and OSN‐type profiles. Please note that this identification is still hypothetical. Otherwise, nonlabeled profiles were designated as N, standing for nonlabeled and nonidentified profiles.

### Synaptic configuration and synaptic connectivity

To estimate the network connectivity between labeled, identified cell types (PNs) and putative OSN and LN cells, and between other nonidentified cells, we determined in selected profiles whether a presynaptic site would contact (or was opposed to) a labeled PN profile, a putative OSN‐, or LN‐type cell, or an unlabeled profile (N profile). To estimate the degree of connectivity, all postsynaptic profiles (labeled PNs and nonlabeled profiles) for a given presynaptic density were counted and designed as a connector (see Fig. 4A and Tables in Supplementary Figs. S3–S8). The postsynaptic profiles were not traced retrogradely.

The raw data for synaptic numbers and synaptic configurations for each neuron type can be found in the 3D pdf file of the respective glomeruli, VA7, DL5,and DM2 (Supplementary Figs. S3–S8).

### Axon profile counting

At the dorsomedial exit of the AL where the medial and mediolateral tract (ALTs) carry axons to the protocerebrum, all HRP‐labeled PN profiles were counted using the Landmark Editors in Amira software in a series of 10 consecutive TEM sections. This procedure was performed independently by three experimenters with similar results.

### Image segmentation

Digitized TEM photographs were imported to open source software ImageJ/Fiji (http://fiji.sc/Fiji; Schindelin et al., 2012) with the plug‐in TrakEM2 or to Amira. Image contrast was enhanced by adjusting the histograms. Segmentation of structures of interest was done with Fiji using the indexing system of the TrakEM2 module that allows the hierarchical order of neuronal features (glomerulus–neuron–synapses) and thus eventually facilitates network analysis. Reconstructed neuron profiles and their synaptic configuration were visualized using the TrakEM2 3D viewer and were exported as wavefront (.obj.) or universal 3D (.u3D) format, indexed, sorted in 3D Reviewer of Acrobat, and converted to Adobe Acrobat pdf format. For visualization, files were exported as .wrl files to Amira. Interactive models were used during reconstruction as an aid and are presented as interactive 3D model.

### Glomerular volume measurements

The measure of volume and surface, and the number of synapses and connectors of all segmented neuronal profiles at the TEM level were obtained in TrakEM2 and indexed and statistically evaluated in Excel. To relate, and to normalize, these PN measurements to *Drosophila* brain structures, the volumes of the glomeruli of interest and the AL volume including the nonglomerular hub were measured in males (*n* = 6). Independently, the complete brain volume, brain areas, and identified glomeruli of interest (DM2, DL5, and VA7) volume were measured in females (*n* = 5) from confocal images (data not shown).

### Intracellular marking and analysis of single neurons

Single‐neuron analysis of PNs and LNs (data provided by Y. Seki, Tokyo University of Pharmacy and Life Sciences, Tokyo, Japan) was done using dual‐channel confocal scans of brains stained with nc82 and costained intracellularly with Lucifer Yellow (Seki et al., 2010), to measure the neuronal and glomerular volume and the innervation pattern of the glomeruli of interest. Neurons were reconstructed using Amira software with a custom‐made SkeletonGraph tool (Evers et al., 2005). The morphologies of PNs and LNs were matched in the glomeruli of interest using imaging registration methods (Rybak et al., 2010). OSN projection patterns were analyzed using genetically expressed markers (Orco‐GAL4; RRID:BDSC_26818; Larsson et al., 2004) and Or22a‐GAL4; RRID:BDSC_9951; Vosshall et al., 2000; Fishilevich and Vosshall, 2005).

### Nomenclature and data repositories

Anatomical directions are given according to the neuraxis. As a digital visual reference system, *Drosophila* brain atlases from Chiang et al. (2011), Jenett et al. (2012), and Shinomiya et al. (2011) were used. Atlases of the *D. melanogaster* AL (Couto et al., 2005; Grabe et al., 2015) were used for glomeruli nomenclature. Brain anatomy nomenclature was decided using the terminology from BrainName (Ito et al., 2014) and the *Drosophila* Brain Atlas at Virtual Fly Brain (www.virtualflybrain.org/site/vfb_site/home.htm).

## RESULTS

**Figure 1:**
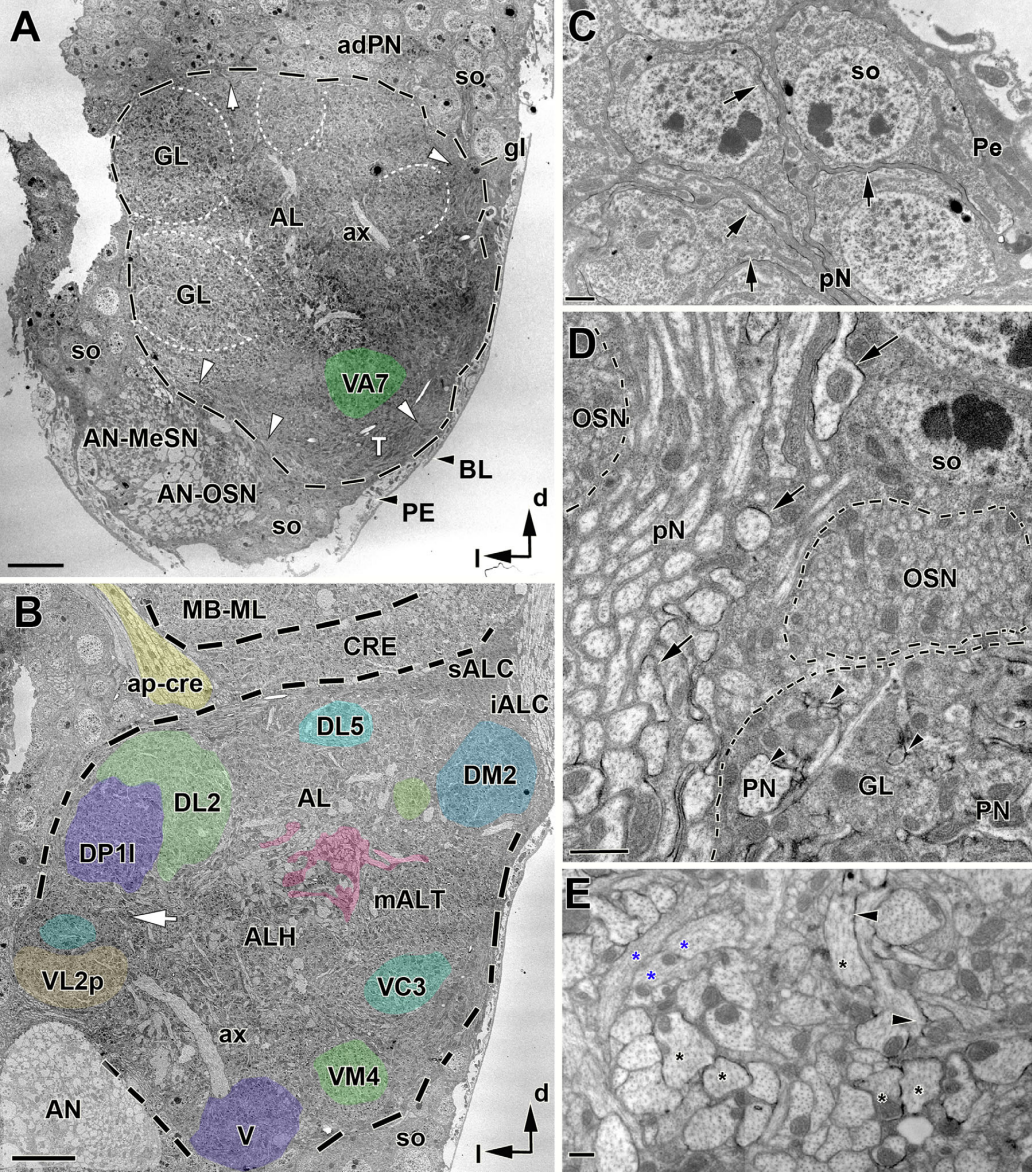
Panoramic view of antennal lobe (AL) organization in the adult brain of *Drosophila melanogaster*. **A**: Transverse TEM section (perpendicular to the body axis) of the right AL at low magnification for general orientation. Layers of neuronal somata (so) surround the central neuropil. The outer border is confined by the perineurium (PE) and an extracellular matrix termed the basal lamina (BL; black arrowheads). The AL neuropil is surrounded by a thin glial layer (black dashed line), and bundles of olfactory sensory fibers emanating from the antennal nerve (white arrowheads in A and OSN in D). Tracheoles (T; respiratory tubes) are found in this zone but not inside the glomeruli (GL). The antennal nerve with sensory fibers originating on the antenna enters the AL ventrolaterally and is composed of mechanosensory (AN–MeSN) and chemosensory (AN–OSN) axonal bundles. Glomerular boundaries are faintly visible, but partly recognized by the arrangement of fiber bundles at their periphery (GL; indicated by white dashed lines). This section is approximately 15 µm from the anterior surface of the AL and one in a series of 50 sections that was used to construct a 3D model of glomeruli and landmark structures (see also Fig. 2D,E). The VA7 glomerulus is colored in green. adPN, anterior–dorsal soma layer; ax, axon; so, soma; gl, glia; GL, glomerulus; d, dorsal; l, lateral. **B**: TEM section of the same AL at a more posterior level, approximately 25 µm from the anterior surface of the AL. The glomeruli are indicated with the color code of the 3D reconstruction presented in Figure 2. Glomeruli VA7 (indicated in 1A), DL5, and DM2 were subjected to a high‐resolution analysis of synaptic connectivity. ALH, central, nonglomerular AL neuropil; AN, antennal nerve; CRE, crepine; ap‐cre, anterior–posterior tract of the crepine; iALC, inferior antennal lobe commissure; MB–ML, medial lobe of the mushroom body; mALT, median antennal lobe tract; sALC, superior antennal lobe commissure. **C–E**: Examples of the black labeling of neuronal membranes of projection neurons used in this study (ectopic expression of a membrane‐bound form of horseradish peroxidase [HRP]; see Materials and Methods). Note the patchy appearance of the labeling. **C**: The arrows indicate HRP labeling of the membrane of PN somata (so) and primary neurites (pN) of the anterior–dorsal somata cluster (ad‐PN in A). **D**: HRP membrane labeling of primary neurites (pN) of the adPN cluster (arrows). Proximal to the border of the AL neuropil (dashed line), an interrupted zone of olfactory sensory neuron (OSN) fibers is followed by the proper synaptic neuropil of the glomerulus (GL) with labeled PN profiles (arrowhead). **E**: In the central AL (ALH in B), labeled axonal processes of PNs (arrowhead and black stars) adjacent to nonlabeled profiles (blue stars) are shown. Scale bar = 10 µm in A; 5 µm in B; 1 µm in C; 2 µm in D; = 0.5 µm in E.

**Figure 2:**
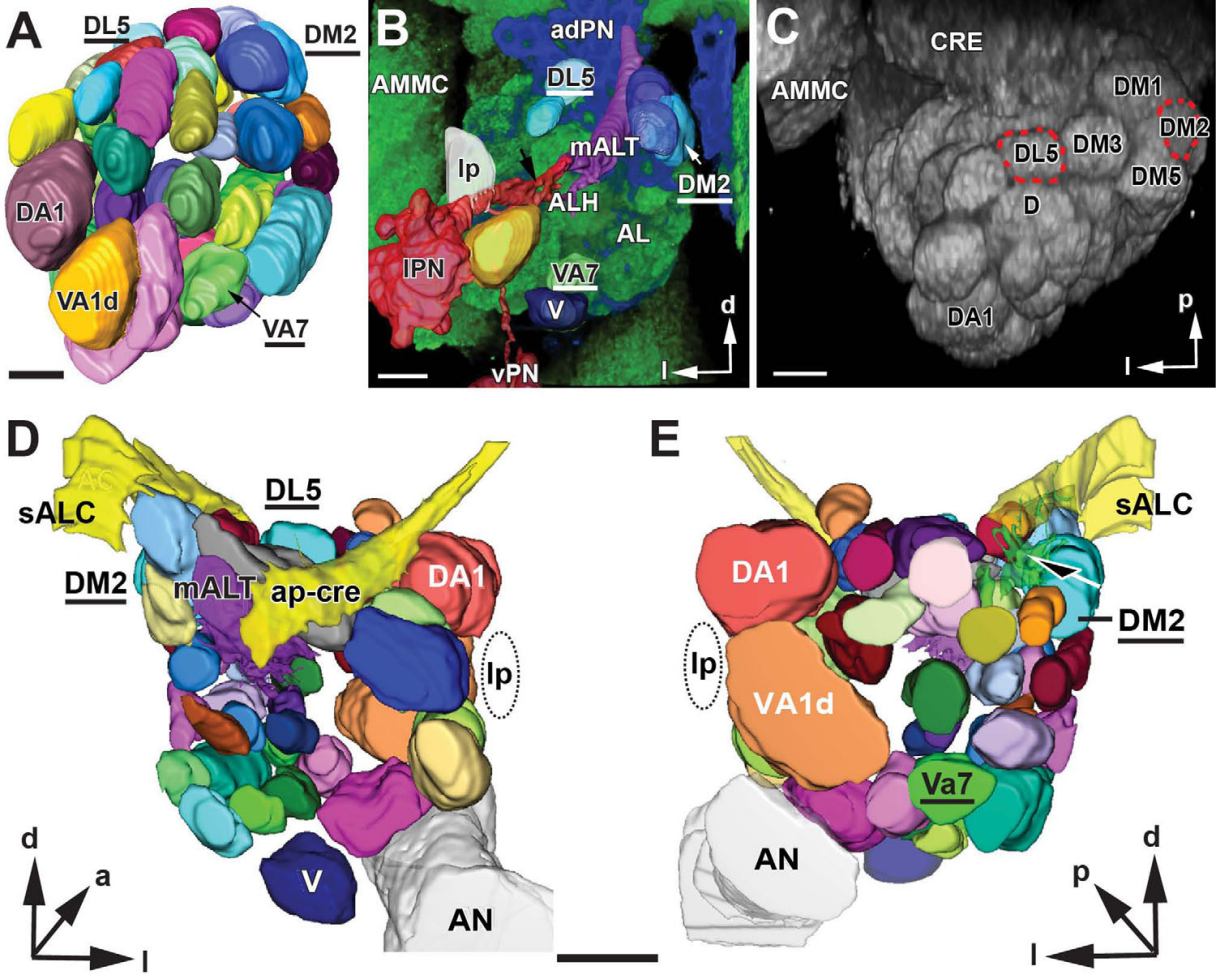
**A**: 3D digital model of the adult antennal lobe (AL) glomeruli in *Drosophila melanogaster* based on confocal microscopy and two fluorescent markers. Glomeruli are colored and named after Grabe et al. (2015). The glomeruli studied here are the VA7, the DL5, and the DM2 (underlined). The VA1d, DA1, and V are landmark glomeruli. The model is available online (Supplementary Fig. S1). **B**: Confocal image showing the AL neuropil stained with a synaptic marker (nc82, green channel) and PN neurons labeled with GFP (blue channel). Superimposed are some segmented and 3D surface‐rendered structures, to illustrate how the 3D model was used during our study. The glomeruli (DM2, DL5, and VA7) and landmark structures are indicated: a lateral somata cluster of projection neurons (lPN), the lateral passage (lp), a bundle of primary neurites emanating from the lPN (black arrow) and a local interneuron somata cluster, and the V glomerulus (V). **C**: Volume rendering of an AL confocal scan in a horizontal view. Identified glomeruli DL5 and DM2 are encircled. **D,E**: A 3D TEM model of the antennal lobe in the adult *Drosophila* male. A posterior (**D**) and anterior (**E**) view of anatomical features generated by the 3D reconstructions of TEM serial sections shown in surface‐rendered mode. The three glomeruli studied here (DL5, DM2, and VA7) are depicted along with landmark structures. Major tracts are the median antennal lobe tract (mALT), the antennal nerve (AN), the primary neurite bundle stemming from the anterior–dorsal somata cluster (black arrow), the lateral passage (lp), the superior antennal lobe commissure (sALC), and the anterior–posterior tract of the crepine (ap‐cre). ALH, antennal hub (a nonglomerular neuropil of the antennal lobe); AMMC, antennal mechanosensory and motor center; adPN, ipsilateral anterior–dorsal somata cluster; CRE, crepine; vPN, ventral PN somata cluster; d, dorsal; p, posterior, l, lateral. The complete 3D TEM model is available online (Supplementary Fig. S2). Scale bar = 20 µm in A–C and D,E.

Our study is based on a series of approximately 800 consecutive ultrathin sections spanning the entire AL of a 3‐day‐old male *Drosophila* fly studied with TEM. Low‐magnification TEM images (Fig. 1) were used together with images made with CLSM (Fig. 2). This correlative approach reinforced the validity of the 3D models of the AL generated with either CLSM or TEM, and provided the basis for the fine‐scale analysis of synaptic structures performed on high‐magnification TEM images at identified glomerular locations. Labeled PNs as well as nonlabeled profiles (N) of AL neurons were reconstructed, and their synaptic configuration, i.e., input (postsynaptic sites) and output (presynaptic sites) in glomeruli VA7, DL5, and DM2, was identified and mapped. In addition, to account for the overall synaptic density, all presynaptic sites were counted in six microvolumes distributed within these glomeruli.

The basic organization of the AL of *D. melanogaster* has been described in detail at the level of fluorescence microscopy (review, see Vosshall and Stocker, 2007). The AL contains approximately 50–54 different and identifiable glomeruli (Couto et al., 2005), innervated by approximately 1,200 receptor neurons (OSNs) (Stocker et al., 1997), the majority of which originate in the ipsilateral third antennal segment and the maxillary palp (Vosshall et al., 2000).

**Figure 3:**
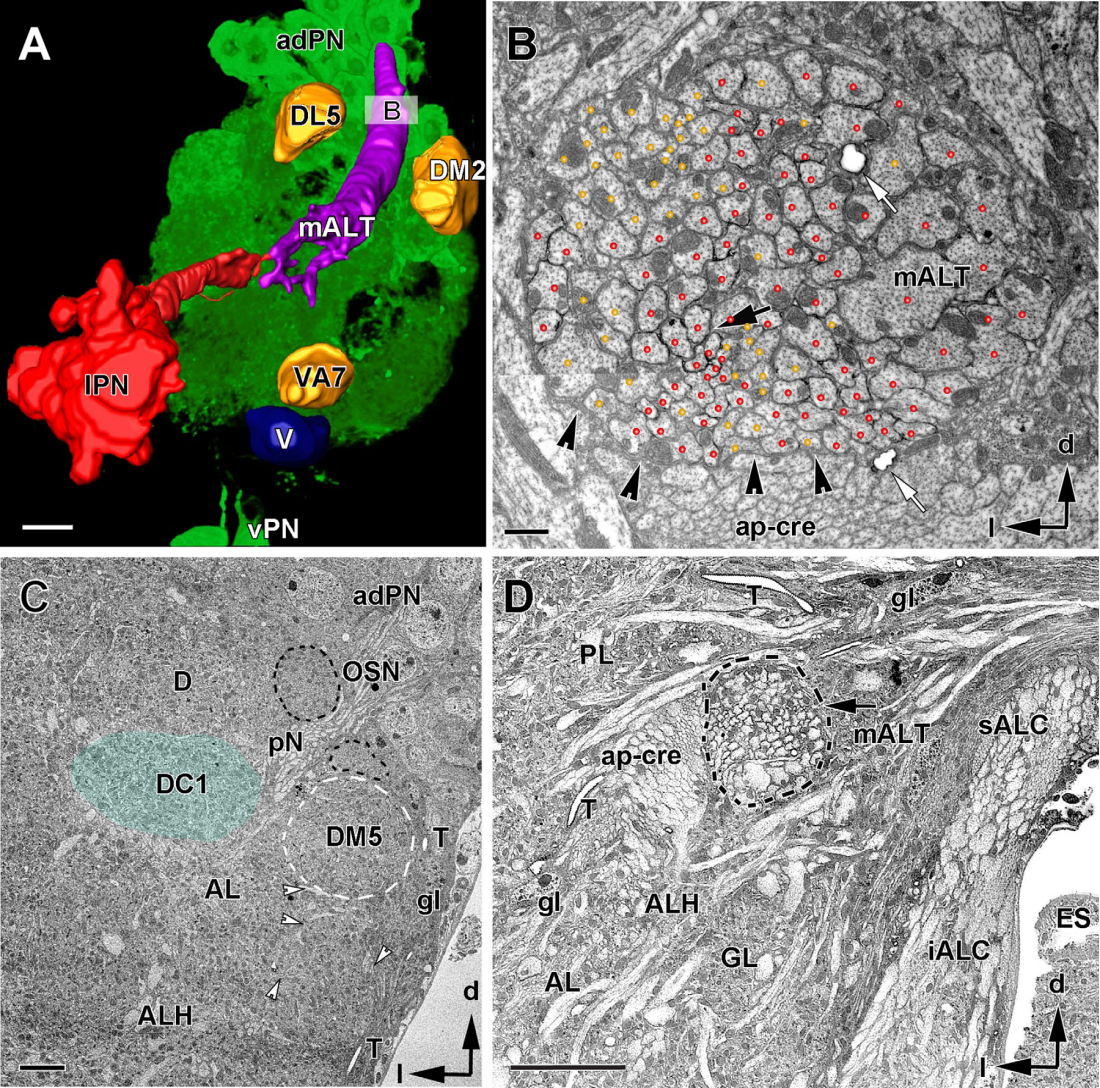
Mapping and ultrastructure of the median antennal lobe tract (mALT). **A**: Confocal image of the right antennal lobe (AL) in a fly expressing GFP driven by GAL4‐GH146 in projection neurons and superimposed onto a 3D image of segmented and surface‐rendered neuronal structures labeled with the same driver. The three studied glomeruli (DM2, DL5, and VA7) and other relevant landmarks are depicted, such as the lateral projection neuron (PN) soma cluster (lPN), the ventral PN soma cluster (vPN), and the median AL tract (mALT). **B**: Ultrathin section across the AL approximately 50 µm parallel to the anterior AL surface (see inset B in A). The axons from a subpopulation of projection neurons running in the mALT are genetically labeled with HRP (black arrow) and are marked with red dots. Non‐HRP–labeled axons are marked in yellow. The mALT contained 146 axons, of which 86 were HRP‐labeled PNs (black arrow). The boundary of the mALT is wrapped by glia processes (darker cytoplasm is indicated by black arrowheads). White arrows indicate tracheoles. **C,D**: TEM images of cross sections at about 25 µm (C) and and 45 µm (D) below the anterior surface of the antennal lobe. **C**: The structure and borders of glomeruli are visible (e.g., dashed white line for the DM5 and shaded blueish area for the DC1 glomerulus). Adjacent structures, such as bundles of thin olfactory sensory cells (OSNs; black dashed line), glial cells (gl), and trachea (T) serving as landmark structures helped to define the glomerular borders. Note also the whirling bundles of neurites and single axons (white arrowheads) marking glomerular boundaries. **D**: TEM image of a cross section of the mALT at the exit of the tract from the AL to the protocerebral lobe (PL) in the posterior AL, and approximately 1 µm posterior to the level depicted in B. At this level, uni‐ and multiglomerular PNs run together in one bundle (arrow, mALT). Note the strong HRP labeling of the mALT axons. AL, antennal lobe; ALH, antennal lobe hub; adPN, anterior–dorsal cluster of GAL4‐GH146 labeled PNs; ap‐cre, anterior‐posterior tract of the crepine; ES, esophagus; g, glia; GL, glomerulus; iALC, inner antennal lobe commissure; mALT, median antennal lobe tract; l, lateral; lPN, lateral PN cluster; PL, protocerebral lobe; T, tracheole; vPN, ventral PN cluster; pN, primary neurite; V, VA7, DL5, DM2, identified glomeruli; sALC, superior antennal lobe commissure; d, dorsal; l, lateral. Scale bar in A and D =10 µm, in B = 1 µm, in C 0.5 µm,.

The overall organization of the AL at the ultrastructural level is shown in Figure 1A and B. The outer surface of the AL (facing the hemocoel, outside the brain) is covered by a glial sheath (the perineurium) that covers the entire brain (Fig. 1A), which itself is covered by an extracellular matrix often termed the basal lamina (Stork et al., 2008). Clusters of neuronal somata are located beneath the perineurium at the lateral, medial, and ventral side of the AL (Fig. 1A,C). They project their primary neurites into a central neuropil made of neuronal fibers of different origin and diameter. Thin axonal profiles from OSNs (AN_OSN and OSN in Fig. 1) reach the AL neuropil along the antennal and palp nerve (Fig. 1A,B; the latter is not shown here). Bundles of primary neurites projecting into the AL neuropil provide useful landmarks (such as the lateral passage). The AL glomeruli enclose a core of nonglomerular neuropil termed “the antennal lobe hub” (ALH in Fig. 1B) (Ito et al., 2014) consisting of primary neurites and axons of other AL neurons. Tracheoles (respiratory tubes) were found in the AL soma layer and could be traced into the subjacent layer of OSN fiber bundles (T and arrowheads, respectively, in Fig. 1A). The AL tracheoles had a diameter ranging from 0.3 to 1 µm and did not penetrate the glomeruli. Some fine tracheoles were also found associated with tracts, such as the mediolateral antennal lobe tract (mALT), and in the soma rind of the AL. At the medial side of the AL (toward the esophagus), the neuropil was close to the perineurium, only separated in parts by a single layer of neuronal soma and glial cells. In the center of the AL, the nonglomerular neuropil, or antennal hub (ALH), we found large axons that were not HRP labeled (diameter from 1.5 up to 3.5 µm) of unknown origin (named ax in Fig. 1A). The remainder of the ALH was filled with bundles of primary neurites of AL neurons (LNs and PNs) and axons of the PN that formed the mALT, which in the AL fused with fibers of the mediolateral antennal lobe tract (mlALT) (Fig. 1B). These axon bundles were traced to the posterior–medial exit of the AL and were counted at high magnification (see below, and Fig. 3). The fiber diameter of these axons ranged between 0.5 and 1.5 µm. The thinnest fiber diameters were measured for the primary neurites: 0.1–0.5 µm.

At higher magnifications the PNs were easily identified by the electron‐dense staining (black in TEM images) of their membrane obtained by the enzymatic reaction of the membrane‐bound HRP, which is exclusively expressed in a subset of projection neurons in flies of the GH146‐GAL4 line (see Materials and Methods, and below). This black staining outlined the somata (Fig. 1C), primary neuritis, and dendritic and axonal parts (Fig. 1D,E). The examples shown in Figure 1C–E belong to the anterior–dorsal GH146 PN cluster (Stocker et al., 1997).

### Correlative light and electron microscopy: the GH146‐nc82 AL model and the ultrastructural 3D model

In *Manduca sexta* and other insects (Oland and Tolbert, 2003), the AL glomeruli are distinctly outlined by glial sheaths, but this is not the case in *Drosophila* (present study). We therefore employed correlative light and electron microscopy for an unambiguous identification of glomeruli. Toward this aim, we compared TEM images in which PN neurons of the GH146‐GAL4 line were labeled with HRP and CLSM images of brains in which the GH146‐PNs expressed GFP and the synaptic sites were labeled with monoclonal antibody nc82 (Fig. 2B,C) (see Materials and Methods for details of genotypes). Reconstructions with confocal microscopy of brains double‐labeled in this way were used to generate a 3D CLSM map (Fig. 2A). The GH146‐nc82 3D model (AL‐LM) (Fig. 2) derived from CLSM sections was compared with current models of the AL by matching landmark structures and the relative position of glomeruli through image registration (data not shown). In parallel, a 3D TEM map was constructed from a series of 50 consecutive TEM sections at intervals of 1 µm throughout the AL (with the exception of the most anterior part of the AL; Fig. 2D,E). These two models are rendered as 3D PDF models and shown in Supplementary Figures S1 and S2.

The AL 3D‐TEM model is shown as surface rendering with annotated landmarks in Figure 2D and E and can be observed from different angles in the interactive 3D model (Supplementary Fig. S2). The three glomeruli under study are shown in posterior (Fig. 2D) and anterior (Fig. 2E) views. A critical and important step was the definition of the border of each glomerulus at the ultrastructural level. Under TEM observation, the borders of *D. melanogaster* glomeruli can be identified by the arrangement of sensory fibers, as they circumscribe and penetrate the glomerulus, as whirling bundles of OSN axons. Most clearly they were seen at the periphery of the AL neuropil, by the entrance of thicker profiles from AL neurons at the glomerular border, and by the glial sheath, which, although extremely thin and relatively patchy, is clearly recognizable in some places. Tracheoles and large axons (>2 µm) were absent in the glomeruli (Figs. 1C, 3C).

The identification of each glomerulus was also aided by its relative position and size in our 3D models (Fig. 2) and by a recently published in vivo brain atlas of the AL based on genetically labeled OSN receptor input used to integrate information, to add newly described glomeruli (Grabe et al., 2015).

The axons of the PNs expressing the GH146‐GAL4 driver were identified at the LM level by their specific expression of GFP (Fig. 3A; see Materials and Methods) and at the TEM level by their black membrane staining (Fig. 3B; see Materials and Methods). In TEM sections, we confirmed that some of the PNs expressing the HRP labeling indeed innervated glomeruli DM2, DL5, and VA7. The primary neurites of uniglomerular projection neurons (uPNs) and multiglomerular projection neurons (mPNs) bundled in the ALH at the center of the AL. Here, they divided, each sending a neurite to a glomerulus and an axon to the posterior–medial exit of the AL (Fig. 3A). The axons merged at about 50 µm from the anterior AL surface as a distinct medial antennal lobe tract (mALT) seen in cross sections at the exit of the AL (Fig. 3D). In a TEM series at higher magnification, 146 PN axons were counted in this tract, of which 86 exhibited black membrane staining (Fig. 3B). This number is very similar to that reported in a study in which PN axons were labeled with GFP using the same driver and counted with light microscopy (Jefferis et al., 2001).

The axons in the mALT were of different diameters, suggesting different classes of neurons, originating in the PN clusters adPN, lPN, or vPN. The thinnest axons had a diameter ranging between 0.2 and 0.5 µm; medium‐sized axons were between 0.5 and 1.5 µm; and large axons were up to 2.5 µm in diameter. The thin axons were found in ventral and lateral positions, and the large axons occupied a medial and dorsal position in the tract. The latter might have their origin in either the lPN or adPN GH146 somata clusters.

### Identification of synapses on labeled PN profiles in three glomeruli

With the aid of the correlative method explained above, synapses were identified and mapped along profiles of labeled PNs and traced and studied with TEM in glomeruli VA7, DL5, and DM2 at three magnifications: in one column (i.e., a collection of serial sections aligned along the z‐axis) at magnification 12,000 × in the VA7 glomerulus and in two columns at magnification 15,000 × in glomeruli DM2 and DL5. Additionally, three columns at magnification 20,000 × were analyzed in the VA7 glomerulus.

The analysis of the VA7 glomerulus derives from a series of 150 sections each 50 nm thick and spanning 7.5 µm of AL tissue in the anterior–posterior direction and 12 µm in the medial–lateral direction. The volume of the sample in which PN tracings and synapses were recorded thus corresponded to approximately 780 µm^3^ and was about two‐thirds of the size of the entire lateral part of the VA7, as measured from confocal scans (approximately 1,340 µm^3^; *n* = 12, for *n* = 6 male AL). In addition, the data for glomeruli DM2 and the DL5 were obtained from a collection of serial sections representing a part of the total volume of each glomerulus (Fig. 14B).

**Figure 4:**
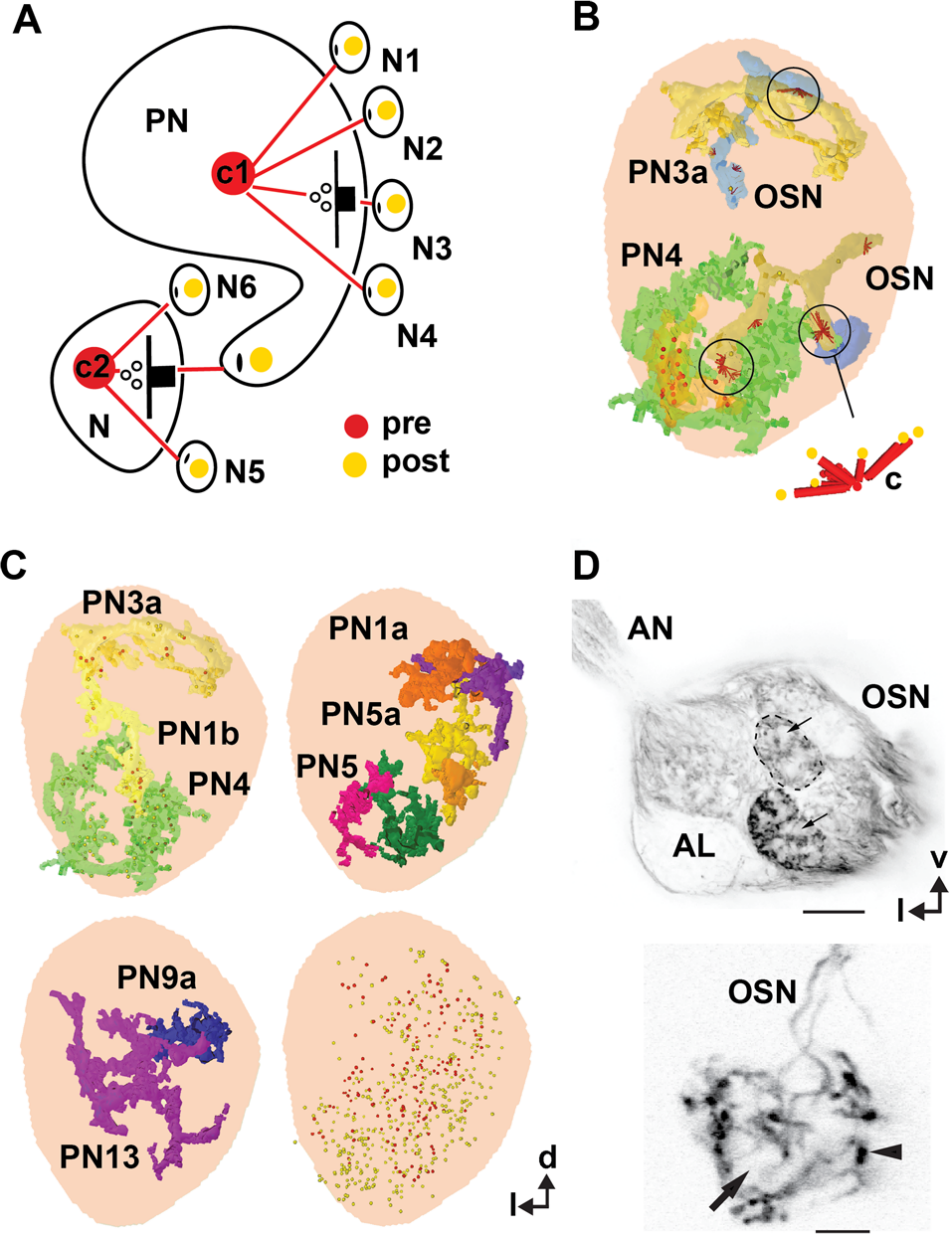
Synaptic structure and distribution of projection neurons (PNs) and olfactory sensory neurons (OSNs) of the antennal lobe. **A**: Definition of synaptic structures that were used in this study to depict synaptic number, synaptic configuration, and connectivity of PNs to unlabeled profiles (N, and N1–N6). PNs are either presynaptic (pre) to their postsynaptic partners (here: PN forms a tetrad to N1–N4) or postsynaptic (here: members of a triad, with N as the presynaptic input to N5, N6, and PN). The red and yellow dots symbolize pre‐ and postsynaptic densities (pre and post, respectively). The connector (c) defines the synaptic configuration (c1: tetrad and c2: triad). **B**: Examples of the distribution of PN (PN3a, PN4) and OSN profiles in the VA7 glomerulus, and their synaptic connectivity. OSN cells form elongated synapses (encircled); also see the inset (c), which symbolizes the OSN connector to PN and other postsynaptic cells (yellow dots). In some areas, OSNs form a high‐density accumulation of output synapses (red dots).**C**: The location of projection neurons (PN3A, PN4, and so on) and the distribution of their synaptic sites within the VA7 glomerulus (frontal view). Depicted are all profiles of PNs segmented in the study that in sum cover the entire diameter of the glomerulus. In the lower right part of the figure the distribution of all pre‐and postsynaptic sites of VA7‐ PNs of this study are shown. **D**: Pattern of glomerular innervation by OSN terminals in the AL. Upper part: high‐resolution confocal scan of OSN fibers forming subdomains within the glomeruli. Lower part: OSN axonal terminals bearing large synaptic boutons (arrowhead). The arrows indicate glomerular zones devoid of OSN innervation. AL, antennal lobe; AN, antennal nerve; v, ventral; l, lateral. Scale bar = 20 µm in D, upper part; 5 µm, lower part.

The borders of glomeruli VA7, DL5, and DM2 were determined principally by spatial alignment of the CLSM model of the AL onto our 3D TEM model (Fig. 2). Then, starting at the glomeruli periphery, labeled and large PN profiles were numbered, traced, and segmented from proximal to distal and along consecutive sections (Fig. 4B,C, Supplementary Fig. S3). It was then generally possible to trace them even to the thinnest ramifications. Accurate PN profile tracing was facilitated by the presence of black membrane staining even in the smallest of the labeled profiles (as thin as 100 nm, and below) and the continuity of mitochondrial profiles along consecutive sections. This method was applied many times in each of the three glomeruli by different investigators, and thereafter the individual tracings were checked against each other for confirmation.

### Synaptic structure

**Figure 5:**
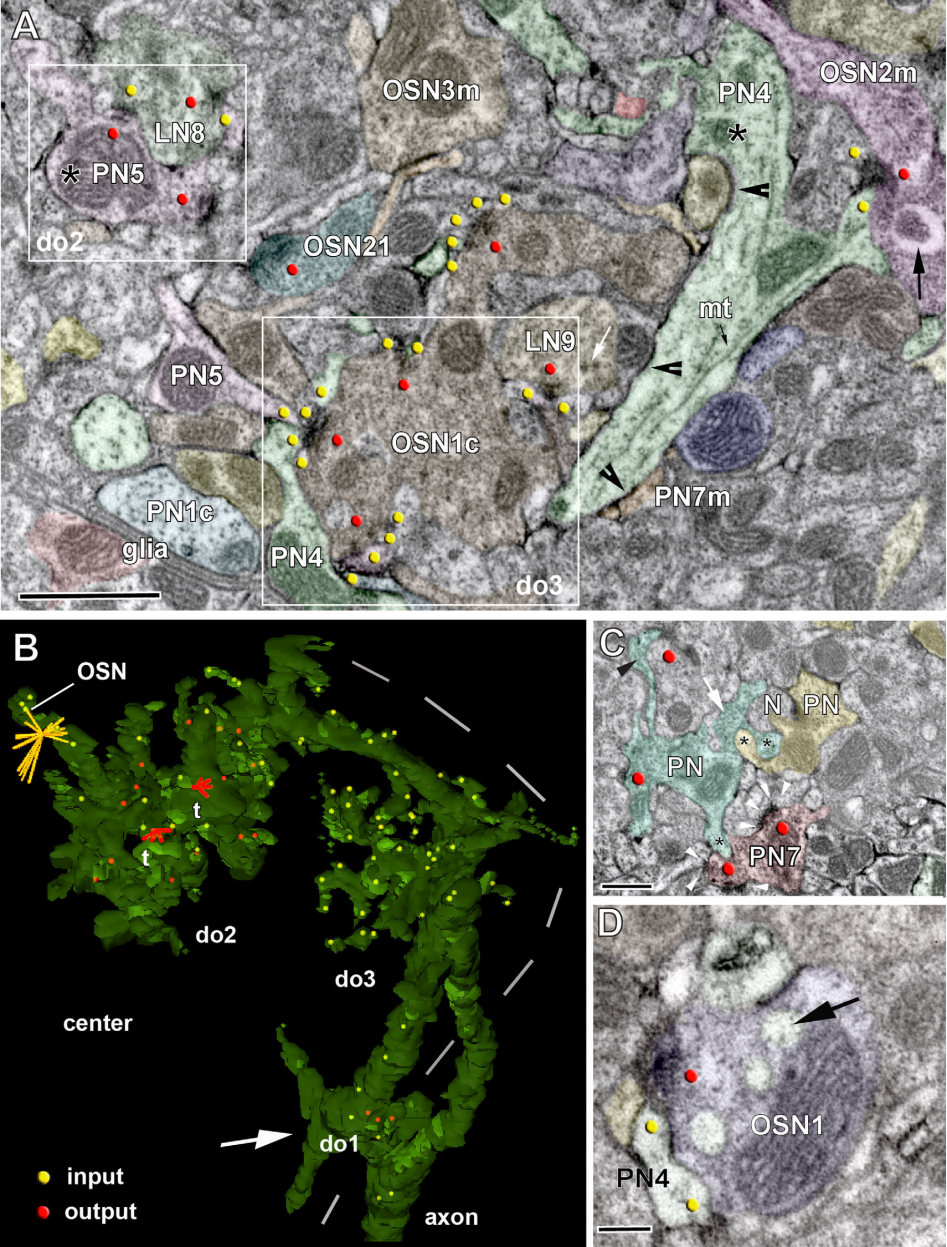
Cell types and synaptic configurations in the VA7 glomerulus. **A**: TEM micrograph of HRP‐labeled projection neurons (PNs) and nonlabeled cell types (putative olfactory sensory neuron [OSN] and local interneuron [LN] profiles). Segmented profiles are colored and were followed throughout a series of about 100 sections. The presence of presynaptic densities or T‐bars and postsynaptic sites is marked by red and yellow dots, respectively. PN profiles are recognized by their stained (black) membrane (arrowheads in profiles PN4) and their cytoplasmic features. They form boutons (swellings, asterisk in PN5, PN4) along their dendrites, bearing presynaptic sites ( = output synapses; red dots) and postsynaptic sites (yellow dots) in their spiny processes. Note the abundance of large mitochondria in PN boutons (e.g., PN5). Large neurites of the PNs are filled with microtubules (mt). Nonlabeled OSN‐type cells (e.g., OSN1c) are characterized by dense, dark cytoplasm filled with small vesicles. They mostly form output synapses onto the spiny processes of PN and on other cell types (indicated by red dots in the OSN1c profile). Presynaptic T‐bars and two types of vesicles, small clear vesicles and larger, dark vesicles (white arrows in LN9), characterize the LN‐type profiles. They form reciprocal synaptic connections to the PN neurons (here: PN5 and LN8). The synaptic connections of the OSN‐ and LN‐type are shown in more detail in Figures 6, 7, 8. **B**: digital reconstruction of a projection neuron profile (PN4) traced from the periphery (dashed line) to the center of glomerulus VA7. The distribution of PN4 input sites and output sites are shown by yellow and red dots, respectively. The output synapses of the PN4 are configured mainly as tetrads (t = red output connector). From proximal (upon their entrance to the glomerulus, white arrow) to distal toward the center, PN4 domains of mixed input/output synapses (do2) and zones with solely input sites (do3) were found. OSN: connector of an OSN‐type cell indicates many postsynaptic targets, including PN4 postsynaptic sites. **C**: A PN7 bouton forms output synapses onto nonlabeled profiles with postsynaptic densities (white arrowheads) and forms mutual invaginations (black stars) onto other projection neurons (PN) and unlabeled profiles (N). **D**: The PN4 is postsynaptic to, and forms invaginations (black arrow) onto a presynaptic putative OSN profile (OSN1). Scale bar = 1 µm in A; 0.5 µm in C; 0.2 µm in D.

The ultrastructure of synapses is defined by active zones, consisting of two membranes with enhanced electron density aligned in parallel and, on the presynaptic site, an electron‐dense structure associated with a cluster of vesicles. In insects, the most common type of presynaptic density is a synaptic ribbon termed the T‐bar (Trujillo‐Cenoz, 1969; Fröhlich, 1985; Meinertzhagen, 1996). The T‐bar is composed of a pedestal and a platform, or table‐top, forming a cytoplasmic matrix with two major molecular components, the proteins Bruchpilot (in the table‐top) and Rim (in the pedestal), which are essential for neurotransmitter release (Wagh et al., 2006; Fouquet et al., 2009; Liu et al., 2011). Interestingly, we also found T‐bars with a very elongated platform (up to 1.5 µm in length) standing on several pedestals. These presynaptic sites were opposed to several postsynaptic profiles (up to 20), and were found in a specific class of nonlabeled profiles (putative OSNs; see the Nonlabeled neuronal profiles section in Results (Figs. 4, 5B, 7). The simple T‐bars were opposed to two, three, four, or five, i.e., dyads, triads, tetrads, and pentads, respectively) or, rarely, even more postsynaptic elements. The tetrad constellation was the most frequently found along PN fibers.

**Figure 6:**
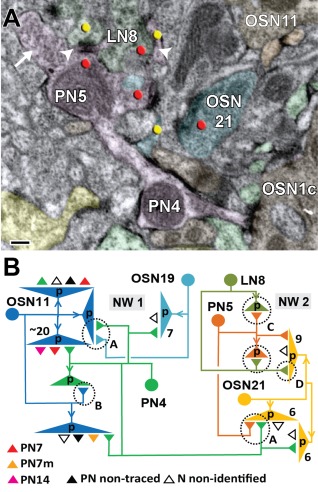
A putative synaptic network among projection neurons (PNs), local interneuron (LN)‐type, and olfactory sensory neuron [OSN]‐type cells. **A**: Synaptic connections between PN profiles and profiles from the other two cell types. In the example, PN5–LN8, input and output PN synapses are in close proximity. The PN5 bouton is presynaptic to the LN8 profile, and receives input from the same LN8 onto a PN5 spiny process (white arrowheads). The PN profile also formed also hair‐like processes around the OSN21 profile. The white arrow in the left upper corner shows a “pocket” in the PN profile filled with vesicles. OSN21 forms an elongated presynaptic density (red dot), as well as a spine, which is postsynaptic to the PN5 (yellow dot). **B**: Microcircuits of neuronal elements in the VA7. The arrows indicate the information flow suggested by the synaptic contacts mapped in our TEM reconstructions. Presynaptic elements are marked with p, and postsynaptic elements are represented by small triangles. In network 1 (NW1) the profiles from OSN‐type cells contain polyadic elongated synapses, contacting between 7 and up to 20 postsynaptic profiles, as indicated by number. OSN‐type profiles thus formed mostly input synapses onto PN profiles (feedforward motif A). PNs were also presynaptic to OSN‐type profiles (feedback motif B: PN4 to OSN11). The right part of the circuit (NW2) shows that OSN‐type and LN‐type profiles are reciprocally interconnected to PN cells (PN5) (reciprocal motif C).Another output synapse from OSN21 targets LN‐type cells (motif D). In summary, the PN profiles receive input from OSN and LN cells, and form feedback (output) synapses onto both cell types. Scale bar = 0.2 µm in A.

**Figure 7:**
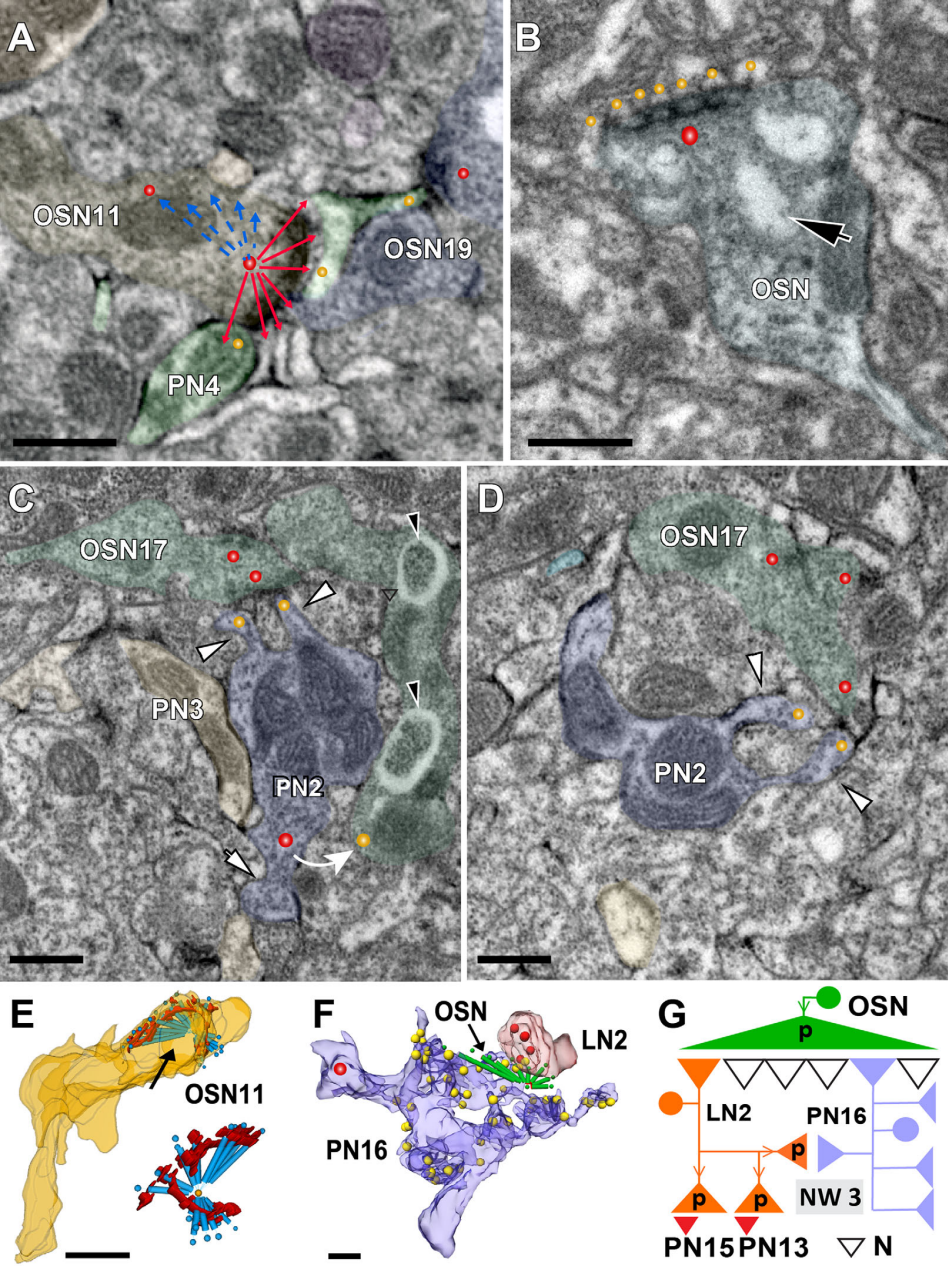
Putative olfactory sensory neuron (OSN) profiles and their synapses. A characteristic feature of OSN‐type profiles is the formation of polyadic synapses with elongated T‐bars consisting of a single platform supported by several pedestals and contacting many postsynaptic profiles. **A**: Example of an elongated, polyadic synapse made by OSN11 in glomerulus VA7. Note the prolonged presynaptic density curving along a distance of approximately 1.5 µm. The connector (red and blue colored lines pointing to postsynaptic elements) indicates the total number of postsynaptic profiles, which in this case is about 20. A labeled projection neuron (PN) profile (PN4) is postsynaptic to OSN11 at two separate sites (yellow dots) The PN4 also formed a spine opposed to another OSN‐type presynaptic site (OSN19) (see also network 1 in Fig. 6B). **B**: Example of a polyadic synapse made by an OSN‐type cell (OSN) in the DM2 glomerulus. This OSN profile contained invaginations (black arrow). The enlarged presynaptic site is equipped with an elongated presynaptic density (red dot) with several pedestals and a large platform opposed to multiple postsynaptic profiles (yellow dots). **C,D**: Typically, OSN‐type profiles were contacted by multiple spines (white arrowheads), which in this example emanated from a PN bouton (PN2), thus contacting and receiving input from polyadic synapses (red dots in OSN17) several times. The PN2 cell formed a feedback synapse to OSN17 (the curved white arrow), which is here outside the plane of the section. Note the gray zones in OSN17 surrounded by a halo (black arrowheads). **E**: 3D reconstruction of the polyadic synapse shown in A. The black arrow indicates the large presynaptic density. **Inset**: The connectors (blue) indicate the number of postsynaptic sites, and the presynaptic density is shown in red. **F**: OSN‐type profiles forming microcircuits with two distinct cell types at the periphery of the VA7. Polyadic synapses of the OSN‐type cells were most frequently encountered at sites with dendritiform PN terminals, which received multiple synaptic inputs (yellow dots in PN16). Here, the elongated polyadic synapse (OSN, green connector, and arrow) is presynaptic to a profile of an LN‐type cell (LN2 bouton, which also contains several output synapses (red dots), and to PN16. **G**: Microcircuitry scheme of synaptic connections of the three cell types shown in F. The OSN‐type cell is directly connected to PN16 (feedforward synapse) and indirectly connected via the LN2 profile forming a serial synapse (network 3 [NW3]). N, nonidentified profiles; PN 13, PN15, PN profiles postsynaptic to LN2. Scale bar = 0.5 µm in A–F.

**Figure 8:**
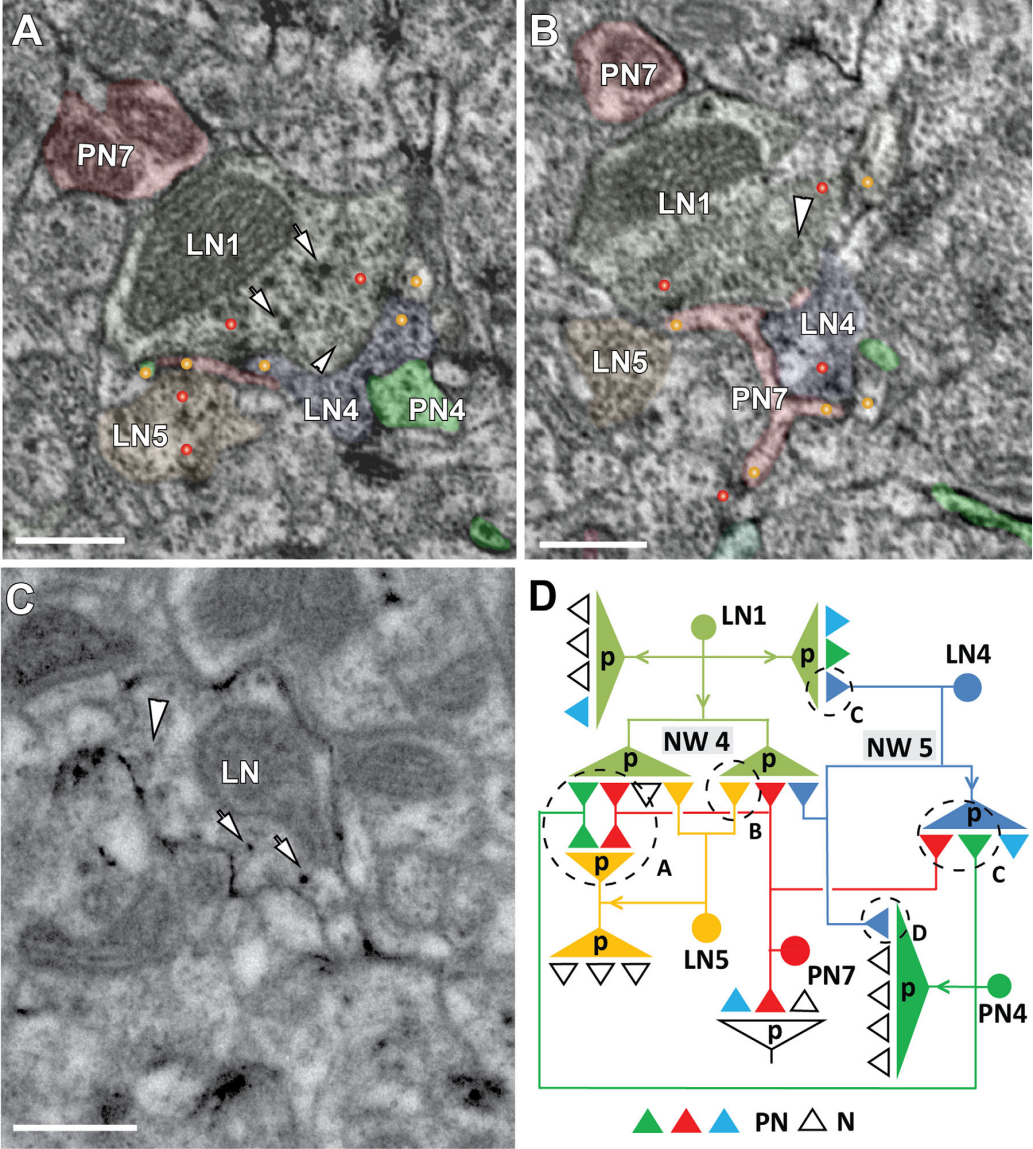
Profiles of putative local interneurons (LNs) and their synapses. **A**: LN‐type profiles were defined by their relatively light cytoplasm and two types of vesicles, small clear vesicles and larger, dark vesicles (arrowhead and arrows, respectively, in LN1). The synaptic configuration of two labeled projection neurons: PN7 (in pink) and PN4 (in green) and three LN‐type profiles (LN1, LN4, and LN5) are shown in **A** and **B**. The LN‐type profiles formed presynaptic T‐shaped densities (red dots). LN1, LN4, and LN5 were presynaptic to fine, hairpin‐shaped and spiny processes formed by PN4 and PN7 (yellow dots; LN1 was also presynaptic to LN‐type profiles LN4 and LN5. **C**: A bouton from a LN neuron labeled with membrane‐bound HRP with a driver specific for local interneurons, Np1227‐GAL4 (Okada et al., 2009), exhibited the two main ultrastructural features used to define LN‐type profiles as those shown in A and C. **D**: Microcircuit diagrams based on the reconstruction from a continuous series of approximately 10 sections showing the full complement of synaptic contacts between PN and LN‐type profiles in a microvolume of about 4 µm^3^. Several network motifs were found: Network 4 (NW4) depicts the convergence of two presynaptic outputs (from LN4 and LN5) onto one postsynaptic site (here: the dendritic spines of PN7 and PN4, motif A [encircled]). Indirect LN synaptic input to the PNs is mediated via a feedforward synapse from LN1 onto LN5 (motif B). Network 5 (NW5): LN1 and LN4 forming a serial synapse onto the PN4 (motif C). Additionally, a local feedback circuit, or reciprocal synapse, was formed between the PN4 and the LN4 (motif D). (The PN4 presynaptic density is not shown in A and B.) LN4 was also presynaptic to PN4 and two other labeled PN profiles. N, nonidentified profiles. Scale bar = 0.5 µm in A–C.

For the description of the synaptic inventory and connectivity of labeled PNs, we annotated the synaptic input and output sites of the PN profiles with red for the presynaptic T‐bar, and yellow for the PN postsynaptic profile. The synaptic configuration of pre‐ and postsynaptic sites for a given synapse was indicated by our segmentations software, TrakEM2, (see Materials and Methods) by a connector, i.e., a graph object, which consists of an origin pointing at the presynaptic site and any number of target points indicating the number of postsynaptic sites. Thus, the connector represents the synaptic configuration of a polyadic synapse (Fig. 4A,B, Tables in Supplementary Figs. S3–S8). The total number of synapses, as well as the number of solely input or output sites, is given per measured neurite volume and per surface of the PN profile (annotated as sy/µm^3^ and sy/µm^2^, respectively).

### PN cells in the VA7 glomerulus

The VA7 glomerulus is located in the ventromedial part of the AL (Figs. 1A, 2A,B). Its boundaries were clearly visible at its medial side flanked by axon bundles of putative OSN axons and more distally by the perineurium of the medial AL. More laterally, adjacent to the central AL neuropil, the boundary was defined by the general course of neuronal profiles and thick axons of PNs entering the glomerulus. A general view of the type of neuronal profiles and neuropil organization is shown in Figures 4 and 5A. The profiles of the PN axons were frequently thicker at the periphery of the glomerulus and became thinner as they ramified inside. Accordingly, we refer to proximal or basal, intermediate and most distal or apical (terminal) parts of the PN dendrite. Along their course through the glomerular neuropil, they formed numerous branches with varicose, bouton‐like swellings, especially in the central region of the glomerulus, and arborizations of very fine branches in zones mostly, but not exclusively, located at the periphery of the glomerulus (dendritiform arborizations type in Figs. 5, 6, 7).

We traced 17 labeled PN profiles within the VA7. These were each continuously segmented over long and short distances in a series of 150 sections. The full assembly of these profiles covered most of the glomerular neuropil in all directions (Fig. 4C). All traced PN cells, including the pre‐ and postsynaptic elements are summarized in an interactive pdf file (Supplementary Figs. S3, S6). We found several large neurites penetrating the VA7 at its ventral, medial, and dorsal border, of which the profile PN4 shown in Figure 5A and B is one example. Their dendritic morphology suggests that these neurites belong to the uniglomerular PNs. Another PN profile with only fine branches within the glomerular neuropil might be a multiglomerular neuron that could only be traced over a short distance (PN7m in Fig. 5A and Supplementary Fig. S3).

### PN input–output sites are spatially segregated within the VA7

Some PN profiles could be traced over relatively long distances, spanning most parts of the VA7 (Fig. 4C). One of these profiles, the PN4, entered the glomerulus at its medial side and bifurcated into two large branches. The PN4 dendrite was traced toward central as well as peripheral regions of the glomerulus (Fig. 5B). We found 30 input (postsynaptic) and 82 output (presynaptic) sites along this profile in a neurite volume (NV) of 15.56 µm^3^. The ratio of the PN4 output‐to‐input sites was 0.37, which is similar to that obtained when this ratio was calculated for the sum of all PN profiles (Table 1, Supplementary Fig. S3). The average synapse density along the completely reconstructed length of the PN4 profile (168 µm) was 0.67 synapses/µm (0.18 synapses /µm for the presynaptic sites and 0.49 synapses/µm for the postsynaptic sites). These values varied in different locations, with the most distal (terminal) arborizations having the highest density of input sites. Close to its entrance into the VA7, a proximal portion of the PN4 profile had a low number of output and input sites (arrow, domain 1 [do1] in Fig. 5B). More distally, side branches emanating from the main neurite, and terminating with fine endings carried input sites only (do3 in Fig. 5B). These terminals were postsynaptic to large globular profiles with output synapses, formed by the putative OSN‐type cells (OSN in Fig. 4B; see also: OSN1c in Fig. 5A). More distally in deeper layers of the glomerulus, we found a domain containing both input and output sites (do2 in Fig. 5B), flanked by another arborizations site with solely input sites and presynaptically contacted by a putative OSN. This spatial segregation of input and mixed input/output sites was also observed in other PN profiles. The PN synaptic sites were thus segregated into different zones of the PN branches, which mostly, but not exclusively, corresponded to peripheral and central regions of the glomerulus (Fig. 5B). However, as is shown here for the PN4 profile, the segregation of synaptic sites was also correlated with corresponding zones of synaptic output by globular putative OSN profiles (see Nonlabeled neuronal profiles section below). PNs also formed nonsynaptic density contacts to each other and to OSN‐type cells, as shown in Figure 5C and D, either by forming mutual PN–PN invaginations or by the PN protruding into the cytoplasm of an OSN‐type cell. The PN–PN invaginations were sometimes filled with vesicles.

**Table 1:** Synaptic Inventory of Cell Types in the VA7 Glomerulus^a^

VA7	vol μm^3^	surf μm^2^	total #	pre #	post #	ratio	sy μm^3^	pre μm^3^	post μm^3^	sy μm^2^	pre μm^2^	post μm^2^
**PN**	95	1002	659	165	494	0.33	6.92	1.73	5.19	0.66	0.16	0.49
**OSN**	25	240	95	80	15	5.33	3.80	3.20	0.60	0.40	0.33	0.06
**LN**	6	60	43	23	20	1.15	7.54	4.04	3.51	0.72	0.39	0.33

a Summary of the quantitative measures derived from all profiles of PN, OSN‐type, and LN‐type cells in the VA7 glomerulus. The data are based on the analysis of 17 PN profiles, 15 OSN‐type profiles, and 9 LN‐type profiles. Pre‐ and postsynaptic sites were counted along each reconstructed profile, and their number and density were calculated per neurite volume and surface. For full statistics, see Supplementary Table S1.

Abbreviations: vol, neurite volume; surf, neurite surface; total, number of all synapses counted per profile; pre, presynaptic site (output synapse); post, postsynaptic site (input synapse); ratio, number of out‐to‐input synapses; sy, synapse; PN, projection neuron; LN, local interneuron; OSN, olfactory sensory neuron.

The larger PN profiles in domain 2 had bouton‐like swellings of different size, reaching as much as 1.5 µm in diameter. Most output PN synapses were found in the larger boutons, but some were located in small boutons or as en‐passant synapses on small‐diameter branches. These synapses had T‐bars, with a platform of about 150–300 nm (i.e., measured in cross section of the synapse) and hence are larger than the platform of T‐bars in LN‐type profiles (see below). Clear and dark vesicles were found close to the T‐bar, or, in the case of large boutons, separated in “pockets” (Fig. 6A).

**Figure 9:**
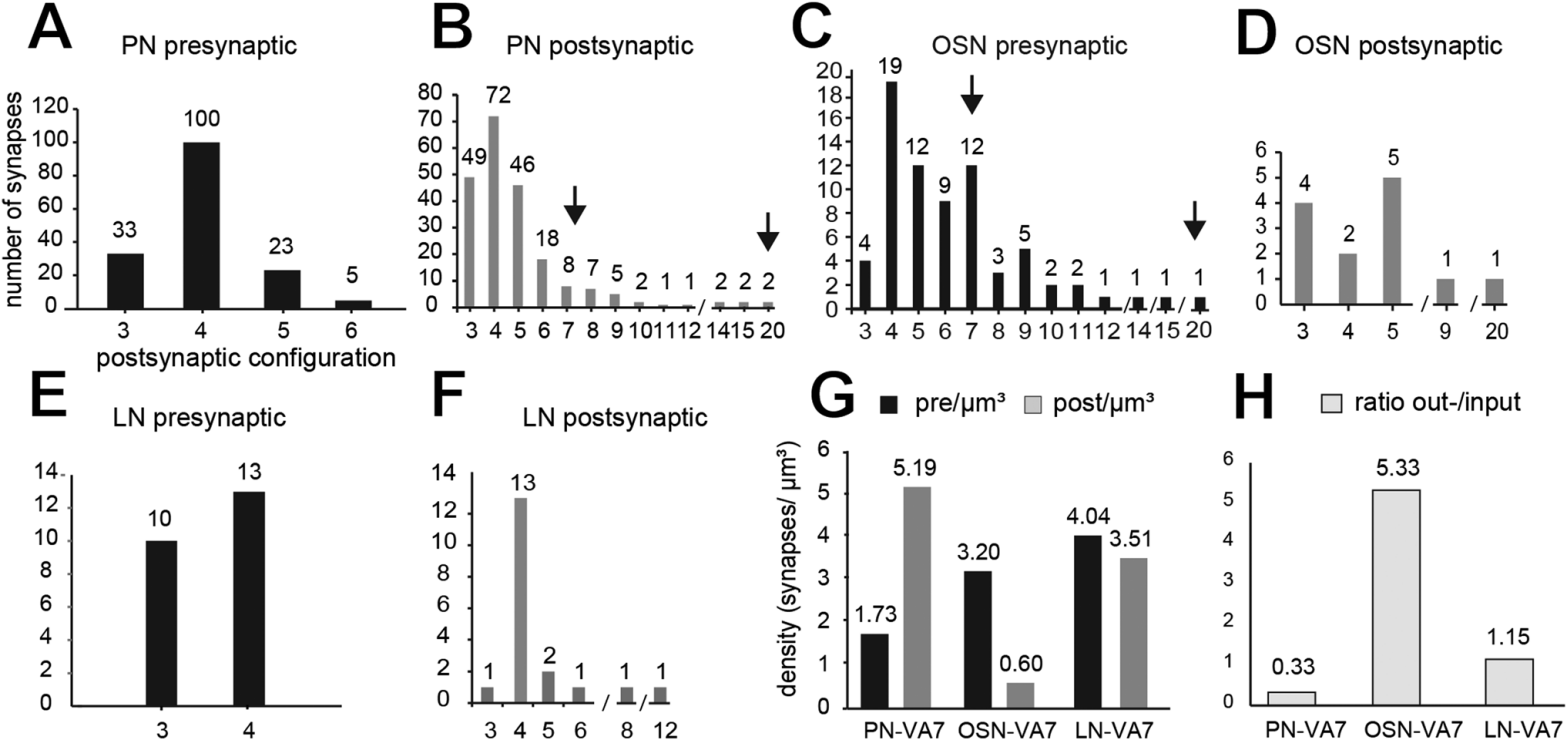
The synaptic configurations of neurons in the VA7 glomerulus. **A,B**: The synaptic configuration of all VA7–PN output synapses (projection neuron [PN] presynaptic) and the configuration of (partly) nonidentified profiles, for which at least one PN profile is the postsynaptic element (PN postsynaptic). Most PNs are tetradic, but additionally are contacted by cells with more than six postsynaptic elements. The arrows indicate the range for polyadic synaptic input from putative presynaptic olfactory sensory neurons (see also corresponding arrows in C). **C**–**F**: Synaptic inventory of OSN‐type cells (**C,D**) and local interneuron (LN)‐type cells (putative local interneurons) (**E,F**). The configuration of synapses formed by OSN‐type and LN‐type cells is different (see OSN and LN presynaptic, respectively). Both cell types most often made synaptic contacts with postsynaptic cells in a tetrad configuration, but OSN‐type profiles had synapses with up to 20 postsynaptic targets (elongated polyadic synapses). The OSN‐type and LN‐type cells (OSN and LN postsynaptic, respectively) received mostly input in a tetrad postsynaptic assembly, but were also postsynaptic to polyadic, elongated presynaptic elements (arrows), indicating that they are postsynaptic to other OSN cells. For full statistics on these cell types, see Supplementary Figs. S3–S5. **G,H**: Synaptic contacts made by all cell types in the VA7 glomerulus. The graph summarizes the quantitative data on synaptic contacts for all cell types (PN–VA7, OSN–VA7, and LN–VA7) in the VA7 glomerulus. **G**: Pre‐ and postsynaptic sites were counted along profiles from each cell type, and their volumetric density was calculated per neurite volume (pre/µm^3^ and post/µm^3^). Each cell type has a characteristic distribution of output and input sites. **H**: Ratio of out‐to‐input synapses for all three cell types. Approximately 30% of PN synapses were output synapses, whereas synapses of the OSN‐type cells formed predominantly output synapses, and LN‐type cells had an almost equal amount of input, and output synapses.

In the majority of these presynaptic sites (approximately 62%), each site was opposed to three, four, or five postsynaptic profiles, the tetrad configuration being the most frequently observed. The density of presynaptic sites was 1.73 /µm^3^, thus being much lower than the density of postsynaptic profiles (5.19/µm^3^) (Fig. 9A,G, Table 1).

The postsynaptic sites on PN profiles were situated in two morphologically distinct arborizations patterns (see the schematic microcircuitry in Fig. 6B) and in different regions of the glomerulus. We found PN profiles with a bouton presynaptic to a putative LN, and a spine emanating from the PN bouton that received at a short distance synaptic input from a putative LN profile (LN8 and PN5 in Fig. 6A,B). This type of reciprocal synaptic connections located at short distances from each other was frequent in the neuropil between PNs and putative LN‐ or OSN‐cell types, and was mostly seen in PN domain 2, i.e., in its intermediate parts. Another synaptic specialization was found at the most distal, terminal arborizations of PN fibers, in the form of very fine, hair‐like extensions spinning around large boutons in OSN‐type profiles and making multiple contacts. In these glomerular areas, we found concentrations of synapses between thin, distal PN fibers and boutons from OSN‐type cells (Figs. 4B, 6, 7C,D).

The networks formed by PN and putative OSN and LN cells were densely reconstructed over a volume of about 1 µm^3^ in the VA7 glomerulus (diagram in Fig. 6B). We found several connectivity motifs, or microcircuits. Network 1 (NW1) comprised OSN‐type profiles with large, elongated presynaptic T‐bars synapsing onto many postsynaptic elements. They were presynaptic onto a set of PN profiles, and onto other OSN‐type profiles (feedforward motifs A of OSN11 in Fig. 6B). The OSN‐type cells received feedback synaptic input from a PN (PN4) (feedback motif B). A second network (NW2) is shown on the right part of Figure 6B. PNs and LN‐type cells reciprocally connected (PN5–LN8), with the PN being presynaptic to the putative LN, and the putative LN being presynaptic to the PN (feedback motif C; see also arrowheads in Fig. 6A). An OSN‐type cell (OSN21) was presynaptic to both an LN‐type cell and to the PN4 (feedforward motif A).

### Nonlabeled neuronal profiles

Although the main focus of this study was to identify and map every single synaptic site along labeled PN profiles, nonlabeled profiles were also taken into account when possible. Some of these additional profiles contained larger, dark vesicles (diameter over 100 nm) and most probably belong to peptidergic or aminergic neurons.

A type of nonlabeled profile exhibiting a number of ultrastructural features that made it possible to distinguish it from other profiles was interpreted here to be putative axon terminals of OSN cells (OSN‐type profiles). These profiles were relatively darker, due to a high density of black dots of about 10–20 nm in diameter (e.g., OSN1c in Fig. 5A and OSN 17 in Fig. 7C), formed “boutons” (swellings along the branches) of the globular type (Cardona et al., 2010a) of a larger size than found in other AL neurons, and received invaginations from other cells, including PNs (Fig. 5C). Dark structures enwrapped by a semitranslucent halo were exclusively found associated with this cell type (Figs. 5, 7C). Putative OSN‐type profiles were found predominantly, but not exclusively, at the periphery of the glomerulus (Fig. 4D). During the segmentation of OSN‐type profiles, it was not possible to trace them over long distances because their axons were of very small diameter and often tightly fasciculate.

No particular, small boutons were formed, as was found for the output synapses in varicose parts of the LN‐ or PN‐type cells (see the bouton of a varicose PN [PN5] compared with a globular OSN profile [OSN1c] in Fig. 5A).

The OSN‐type profiles formed mostly output synapses, which in many cases were polyadic synapses of the tetrad type (OSN‐type 1 synapse). One type of synaptic site observed exclusively in OSN‐type profiles had an elongated presynaptic density, with a single platform resting on several pedestals and opposed to seven or more (up to 20) postsynaptic elements (OSN‐type 2 synapse, shown in Fig. 7A,B,E, Table 1). These large, elongated “polyads” were associated with clear vesicles (diameter approximately 20 nm). The length of the complete presynaptic density shown as example (OSN11 in Fig. 7A,E) measured 1,500 nm and extended over a region of approximately 1,000 nm (18 sections), whereas a “simple” T‐bar of other AL cell‐types (PN and LN) found in this study measured 100–300 nm in cross section. The high number of postsynaptic processes resulted in part from the fact that some profiles from a single cell contacted the enlarged presynaptic density several times (multiple spines). As shown in Figure 7 C and D, PN profiles formed several distinct spiny processes onto a single OSN‐type bouton. We counted these processes as separate PN postsynaptic contacts because each single process had a postsynaptic density and was separated by other processes. In some cases PN postsynaptic profiles formed hair‐like extensions that entangled and looped around an OSN‐like presynaptic element (Fig. 7A,F).

#### OSN network

The OSN‐type cells formed predominantly output synapses onto PN cells and less frequently onto LN‐type cells, but sometimes were also postsynaptic to these two cell types. In addition, OSN‐type profiles made synapses onto other OSN‐type cells (network NW1 in Fig. 6B). A representative overview of a dense reconstruction of labeled PNs and OSN‐type and LN‐type profiles is shown in Figure 6B. In some places where OSN‐type and PN profiles were opposed to each other, we found that the PN profile formed invaginations within the OSN profile (Fig. 5D). At the periphery of the VA7, we found a network motif that formed a triadic configuration of OSN‐type and LN‐type cells with PN cells in the most distal portion of the PN dendritic tree, with a high density of PN input synapses (here: the axonal terminal endings of the PN16 profile) (Fig. 7F; network NW3 in Fig. 7G). A presynaptic OSN‐type cell targeted nine postsynaptic profiles, including an LN‐type profile and a PN cell. This LN‐type cell (LN2 in Fig. 7F,G) was also presynaptic to PN16.

For a group of 15 OSN‐type profiles found in the VA7, we performed a detailed synaptic and network analysis (Fig. 9, Table 1, Supplementary Fig. S4). The number of output synapses along these profiles had a bimodal distribution, with the two most frequent postsynaptic configurations having either four, thus forming a tetrad (OSN‐ type 1, the tetrad type) or more than six postsynaptic elements (OSN type 2, the elongated type) (Figs. 7, 9C). The OSN type‐2 targeted 56% of all postsynaptic profiles versus 44% postsynaptic partners for OSN type 1 (Table in Supplementary Fig. S4). As 10% of OSN‐like profiles were contacted by profiles bearing the OSN type2 synapses, we have indirect, but strong, evidence for synaptic connections between OSNs within the VA7 (Fig. 9D).

In VA7 the number of OSN‐type synaptic output‐to‐input sites, i.e., the ratio of its pre/postsynaptic sites was around 5:1 and is the highest value found among all cell types studied here. For the PNs, this ratio was the opposite. Around 40% of all presynaptic sites of OSN‐type cells with OSN‐type 2 synapses contacted six or more (up to 20) postsynaptic profiles. The total synaptic density (input and output) of OSN‐type cell profiles was low compared with the synaptic density of PN and LN‐type cells: ≈ 4 synapses/µm^3^ versus ≈7 synapses/µm^3^, respectively. The presynaptic density of OSN‐type cells was 3.2/ µm^3^. Lastly, the OSN‐type cells had the lowest number of postsynaptic input sites, ≈0.7 sy/µm3, versus ≈5 sy/µm^3^ and ≈3 sy/µm^3^, respectively for the PN and LN cell type (Table 1). The OSN‐type fibers formed clusters in the periphery and in more central parts of the glomerulus, where the fine, distal endings of PNs were nested and clearly distinguishable because of their light cytoplasm and labeled black membranes. The highest density of postsynaptic profiles of PNs was found in these regions (Figs. 4B,D, 7F).

### The LN‐type cell

A second type of nonlabeled profile had features also observed in profiles labeled with HRP with a driver specific for LNs. We therefore believe that these nonlabeled profiles belong to LN cells and refer to them as LN‐type profiles. These profiles had light cytoplasm and two types of vesicles, clear (approximately 40 nm in diameter) and dark (50 nm or larger) (Figs. 5, 6, 8). LN‐type profiles formed small axonal swellings (boutons) of the varicose type (Cardona et al., 2010b) containing synapses with distinct T‐bars opposed to several postsynaptic elements in either triad or tetrad configuration (Fig. 9E). The T‐bar of the active sites in LN‐type profiles measured between 100 and 200 nm in cross section, which is slightly smaller than the T‐bars measured in PN profiles. These features were also found in a preparation in which LNs were genetically HRP‐labeled with the help of the GAL4‐1227 driver, which is known to label one class of LNs (called LN1) that has pan‐neuronal arborizations in most AL glomeruli (Okada et al., 2009; Seki et al., 2010) (Fig. 8C). LN‐type profiles were densely reconstructed in a portion of the VA7 glomerulus spanning approximately 4 µm^3^. A small network of LN‐type cells with PN cells is shown in Figure 8. The LN1‐cell type profile contained T‐bars, a dense core, and clear vesicles (arrow, arrowhead in Fig. 8A,B), and was presynaptic onto other LN‐type profiles (LN4 and LN5) and onto PN profiles, (PN4 and PN7). The constellation of the LN‐type networks (NW 4 and 5) is depicted in Figure 8D. Presynaptic sites of two different LN‐type profiles contacted two spiny protrusions of the PN4 and PN7 (see also Fig. 8A,B). This synaptic convergence was occasionally also seen at other locations onto fine PN spines. LN1 was also presynaptic to LN5 and LN4, and the latter formed reciprocal synapses with the PN4 (network NW5 in Fig. 8D). Thus, the LN1–LN4–PN4 and LN1–LN5–PN4 profiles formed a serial synapse. Another type of synapses, not shown here, was formed by an LN‐type cell onto OSN‐type cells. LN‐type cells also received presynaptic input from OSN‐type cells.

The volumetric synaptic density along LN‐type profiles (output and input synapses) was approximately 7 synapses/µm^3^. This is similar to the synaptic density measured in PNs in VA7 and DL5, and higher than in OSN‐type cells. The output‐to‐input ratio for LN‐type cells was 1.15, which is intermediate between the ratios for the PN and OSN‐type profiles (Fig. 9G, Table 1). These numbers must be considered with care, as they represent synapse counts in a relative small neuronal volume of about 6 µm^3^ of LN‐like neurite volume (for comparison: PN about 100 µm^3^, and OSN about 25 µm^3^) and because most counts were done in a region with LN‐boutons.

### Glomerulus DL5

**Figure 10:**
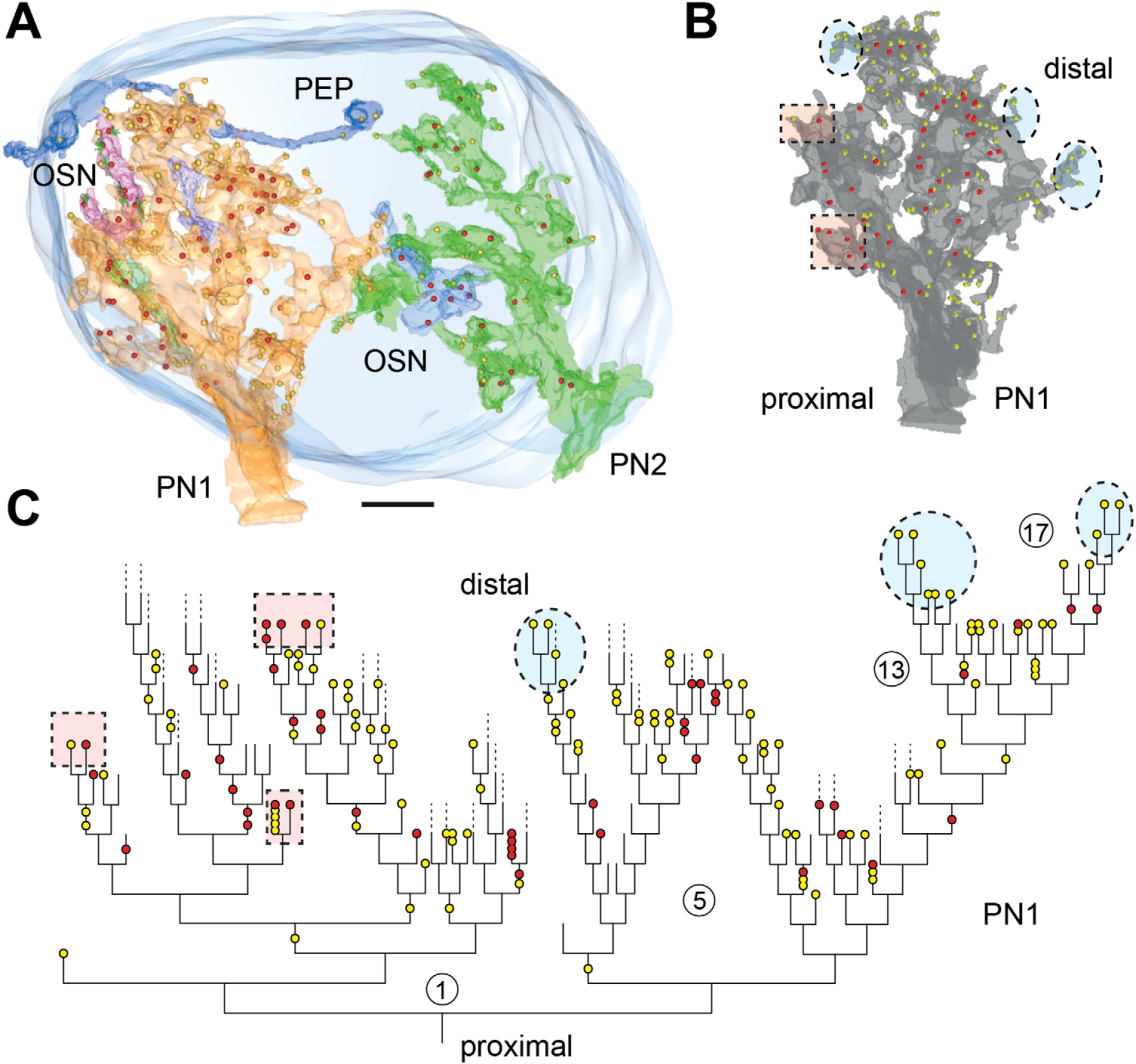
Synapse mapping in glomerulus DL5. **A**: In this reconstruction the glomerulus DL5 is shaded in blue. Two projection neuron (PN) profiles (PN1 and PN2) occupied separate regions of the glomerulus and probably belong to different PN cells because their proximal shafts enter the glomerulus at different sites. The neurite volume of PN profiles in which presynaptic (red dots) and postsynaptic (yellow dots) sites were identified is 35 µm^3^ for PN1 (in yellow) and 15 µm^3^ for PN2 (in green). OSN, olfactory sensory neuron (OSN)‐type profiles; PEP, a putative peptidergic neuron. See also the interactive 3D pdf in Supplementary Fig. S7). **B**: A reconstruction of the PN1 dendritic tree. Shaded and encircled areas refer to the location of synapses as shown in C. **C**: Dendrogram depicting the distribution of input (yellow) and output (red) synapses along different branching levels of the PN dendritic tree from level 1 (the entry neurite, proximal) to level 17 (the PN terminal branches, distal). Dashed lines indicate that the respective arbor could not be followed. Note that individual branches might terminate at any arborizations level starting at level 2. The number of synapses increases with the degree of arborization. At levels 0–4 and 15–17, solely input synapses were found. Levels 5–15 are a mixed zone of input and output synapses. Output synapses were found most frequently at levels 6–12. At terminal branches solely input synapses (blue, encircled), or terminals with input/output synapses (pink, encircled) were found. The number of terminals without synapses is 16 ( = approximately 20%). Scale bar = 3 µm in A.

In the DL5 glomerulus we investigated two large PN profiles that most probably belonged to different neurons because they had different entry points (the PN1 entered the glomerulus ventrolateral and the PN2 ventromedial; Fig. 10A) and represented a neurite volume of approximately 35 µm^3^ and 15 µm^3^, respectively. The ratio of output‐to‐input synapses was on average 0.36 for both profiles. We found synapses between PN profiles, between PN and OSN‐type cells, between OSN‐type cells, and a rare case of an output synapse from a PN profile onto profiles bearing dense‐cored vesicles that were probably of peptidergic nature (PEP in Fig. 10A, Supplementary Fig. S7).

The analysis of the PN1 profile comprised a dendritic cable length of approximately 219 µm, with 121 branching points, i.e., a branch density of one every 2 µm of dendritic cable. On average we found 0.77 synapses/µm, with 0.22 presynaptic sites/µm, and 0.55 postsynaptic sites/µm. PN1 zones bearing either output or mixed output/input synapses were partially segregated along the proximal–distal gradient, as observed before in the VA7 (Fig. 5B), i.e., from the thicker profiles close to the entry into the glomerulus, across the center, and toward the most distal branches. Terminal dendrites with solely input or mixed input–output synapses were found (blue and red dashed regions in Fig. 10B). A dendrogram analysis shows that the most proximal parts (branching level 1–5) contained only input synapses. The highest degree of arborizations for the PN1 was branching level 17 (Fig. 10C).

The overall PN volumetric synaptic density was 5.21 synapses/µm^3^ (Table 2). The most common synaptic output configuration of the DL5–PNs was the tetrad, but we also observed up to seven postsynaptic targets contacted by DL5–PNs. Postsynaptically, DL5–PNs were contacted mostly by cells in a tetradic configuration (Fig. 12A,B). Statistical parameters such as the number of output and input synapses of PNs in the DL5 were in between what was found in the VA7 and DM2 glomerulus, whereas the ratio of output‐to‐input synapses, 0.36, was highest in the DL5 (Fig. 13, Supplementary Fig. S7).

**Table 2:** Synaptic Inventory of Projection Neurons (PNs) in the VA7, DL5, and DM2 Glomeruli^a^

glo	vol μm^3^	surf μm^2^	total #	pre #	post #	ratio	sy μm^3^	pre μm^3^	post μm^3^	sy μm^2^	pre μm^2^	post μm^2^
VA7	95	1002	659	165	494	0.33	6.92	1.73	5.19	0.66	0.16	0.49
DL5	49	390	256	68	188	0.36	5.21	1.38	3.82	0.66	0.17	0.48
DM2	48	423	176	37	139	0.27	3.68	0.77	2.91	0.42	0.09	0.33
					ø	0.32	5.27	1.30	3.97	0.58	0.14	0.43

a A: In all glomeruli, the volumetric density of presynaptic sites on PN dendrites is lower compared with that of postsynaptic sites. The overall synaptic density in the DM2 glomerulus is considerable lower compared with the DL5 and VA7. B: Although the synaptic density fluctuates in the three glomeruli, the ratio of pre‐to‐postsynaptic sites is invariant at a value of about 0.30, i.e., about 30% of PN synapses are output, or feedback synapses. Volumetric synaptic density is represented in sy/µm^3^ and synapse density per surface in sy/µm^2^. For full statistics, see Figures S3–S8.

Abbreviations: vol, neurite volume; surf, neurite surface; total, number of all synapses counted per cell type; pre, presynaptic site (output synapse); post, postsynaptic site (input synapse); ratio, number of output‐to‐input synapses; sy, synapse.

**Table 3:** Synaptic Configuration of Projection Neuron (PN) Profiles Across Glomeruli^a^

A PN presynaptic						
	VA7 %	total	DL5 %	total	DM2 %	total
dyad	‐	‐	‐	‐	12	4
triad	20.5	33	27.3	18	27	9
tetrad	62.1	100	33.3	22	52	17
pentad	14.3	23	25.4	17	3	1
sextad	3.1	5	7.5	5	3	1
> 6			6	4	3	1

B PN postsynaptic						
	VA7 %	total	DL5 %	total	DM2 %	total
dyad	‐	‐	8.2	13	6	7
triad	22.8	49	17.6	28	29	36
tetrad	33.5	72	25.2	40	36	44
pentad	21.4	46	28.3	45	18	22
sextad	8.4	18	11.3	18	7	9
> 6	14	30	9.4	15	4	5

a **A**: The percentage of synaptic configurations (dyad, triad, tetrad, etc.) of all synapses in which the presynaptic site was located in the PN profile is shown. In all, 17 PN profiles from the VA7, 2 PN profiles from the DL5, and 5 PN profiles from the DM2 were evaluated. Triad and tetrad were the most frequent configuration among these synapses (62% tetrads in VA7). **B**: The percentage of synaptic configurations of all synapses in which one or more of the postsynaptic site belonged to a PN profile. Here, more frequently sextads and higher configurations were found. Total, absolute number of synapses found for each type of synaptic configuration.

### Glomerulus DM2

**Figure 11:**
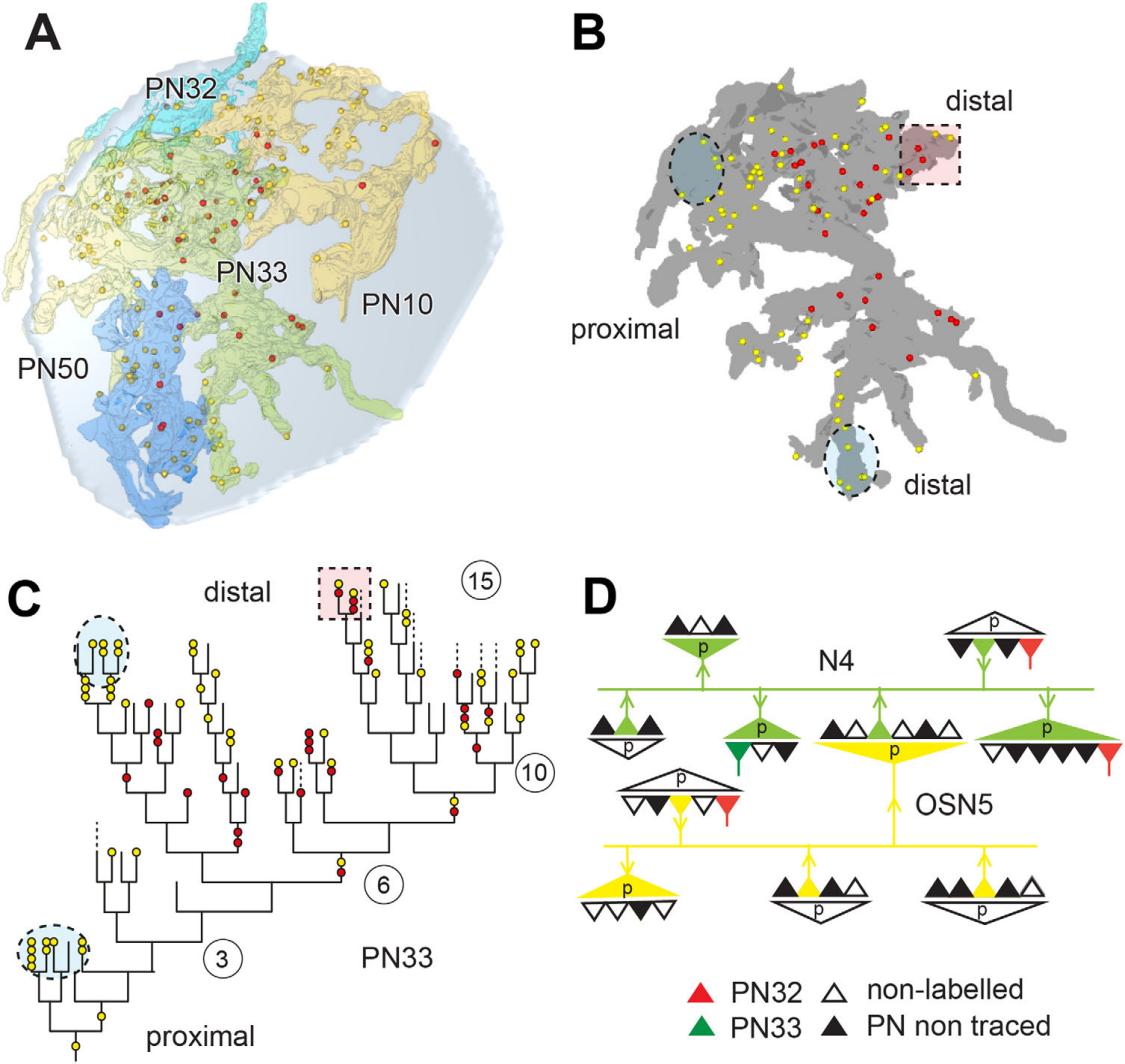
Synapse mapping in the DM2 glomerulus. **A,B**: Reconstructions of projection neurons (PNs) with their presynaptic sites indicated by red dots and their postsynaptic sites by yellow dots. See also the interactive 3D pdf in Supplementary Fig. S8. **A**: Here the PN profiles, situated at the periphery of the DM2 glomerulus are postsynaptic. **B**: Synaptic composition of the PN33 profile. Note that the PN33 profile receives mostly input at the periphery (yellow dots), whereas mixed input–output zones are more toward the center (blue and pink shaded areas. respectively). **C**: Dendrogram depicting the distribution of input (yellow) and output (red) synapses along different levels of the PN dendritic tree from level 0 (the entry neurite, proximal) to 15 (distal). Upon branching, level 6 input synapses are only at terminal positions. Note that at higher branching levels, terminal branches had either solely input (blue shaded, encircled) or mixed input–output (pink shaded, encircled) synapses. **D**: Connectivity scheme of labeled PNs, olfactory sensory neuron (OSN)‐type cell (OSN5), and nonidentified cells (N4): The nonidentified N cells and the OSN‐type cell formed synapses mostly onto PNs, but received feedback synapses from PNs.

Six PN profiles were traced in the DM2 glomerulus, two of which most probably belonged to different neurons. In addition, this glomerulus showed the cellular composition described above for the VA7 and DL5 glomeruli (for example, see the OSN‐type cell in Fig. 7B). Two OSN‐type profiles were densely reconstructed and, as described above for the VA7, these profiles had synapses with elongated presynaptic densities. In this case, the elongated OSN‐type presynaptic sites were found opposed to PN profiles PN32 and PN33 (Fig. 11D). As observed in the other glomeruli, output and input synapses along PN profiles in the DM2 were partially segregated, with a higher percentage of input synapses in the most distal branches, located in peripheral zones (Fig. 11A,B). The dendrogram of the PN33 profile revealed that the most proximal portion of the dendritic tree carried solely input synapses up to branching level 6. Zones with mixed input–output synapses were found at level 10. In the most distal parts of this tree, we found mostly input synapses, but some terminal zones also contained mixed input–output synapses (Fig. 11C). This type of segregation was similar to our findings for the PNs in the VA7 and DL5 glomeruli. The highest degree of branching in this reconstruction was level 15.

**Figure 12:**
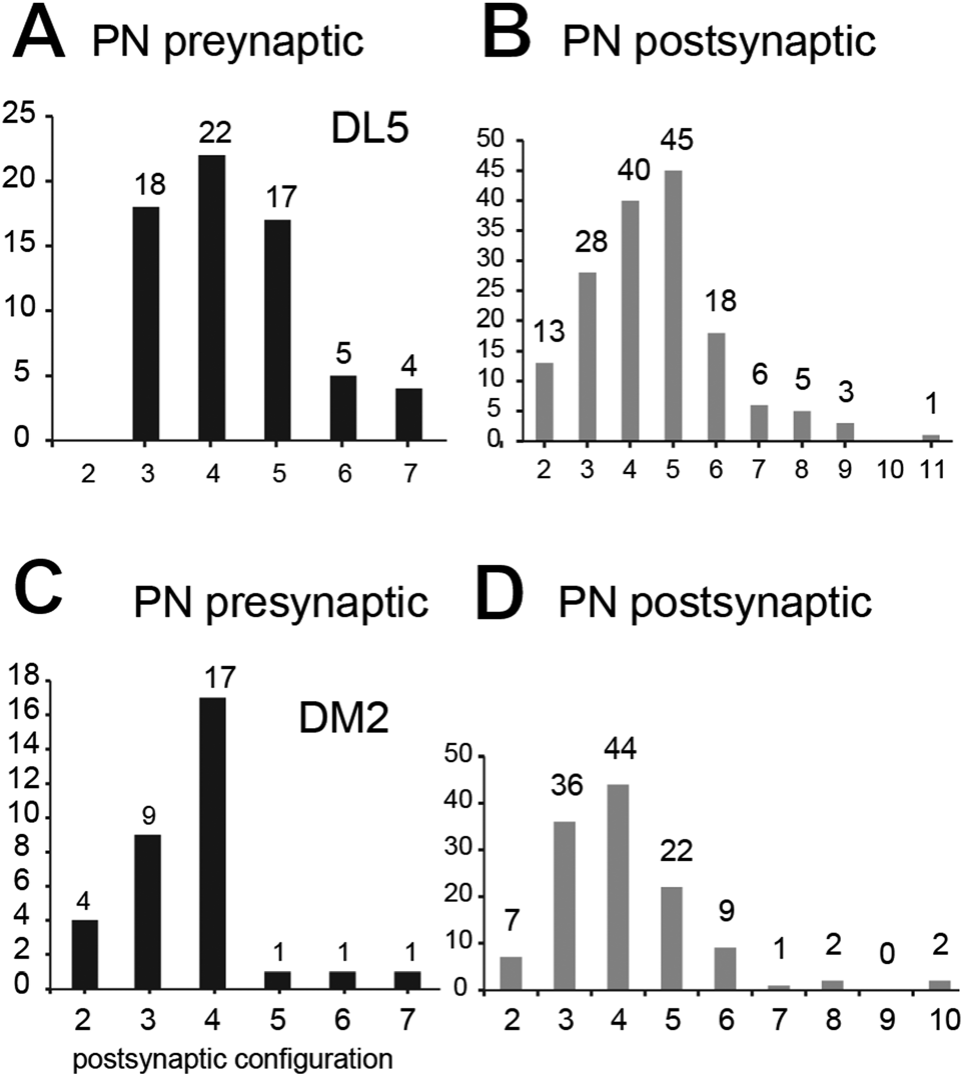
Quantitative data of the synaptic configuration of projection neurons (PNs) in glomeruli DL5 and DM2. **A,B**: For the DL5–PN profiles, the synaptic configuration of 66 presynaptic sites (PN presynaptic) were evaluated. Presynaptic sites were configured mostly as triads, tetrads, and pentads. Polyadic synapses with more than seven postsynaptic sites for a given presynaptic site were exceptions: only four cases were found with seven partners. **C,D**: Quantitative measurements for PN profiles in the DM2 glomerulus. Comparison of synaptic configurations of DM2–PN output sites (PN presynaptic) and DM2–PN input sites (PN postsynaptic). The most frequent configuration of presynaptic sites of DM2–PNs was triads and tetrads. Between 18% and 9% of DL5–PNs and DM2–PNs, respectively, are postsynaptic to profiles with elongated synapses (contacting more than seven postsynaptic sites). Numbers indicate the amount of synapses of each synaptic configuration.

**Figure 13:**
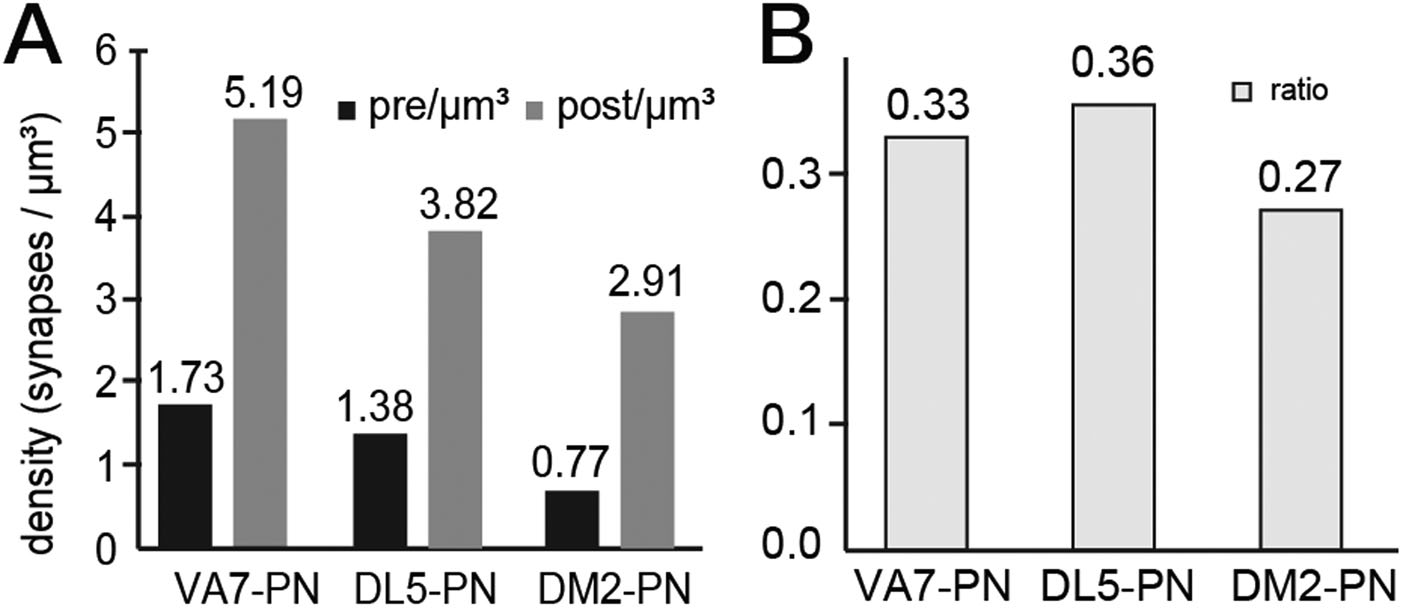
Summary of synapse numbers and synaptic configurations among projection neuron (PN) profiles in glomeruli VA7, DL5, and DM2. **A**: In all glomeruli, the neuron volumetric density of PN presynaptic sites was lower relative to that of postsynaptic sites. The overall synaptic density in the PN profiles reconstructed in glomerulus DM2 is considerably lower than that measured among PN profiles in glomeruli DL5 and VA7. **B**: Although different synaptic densities were found in the three glomeruli, the ratio of pre‐to postsynaptic sites was the same in the VA7 and DL5 glomeruli (about 0.35) and was slightly lower in the DM2 (0.27). On average, about 30% of PN synapses are feedback (or output) synapses.

Along the length of the PN33 profile, we measured an average synapse density of 0.42 synapses/µm (0.13 sy/µm for presynaptic sites and 0.29 sy/µm for postsynaptic sites). The overall volumetric density of synapses in PN profiles of this glomerulus (3.68 /µm^3^) was lower compared with that in the VA7 and DL5 (Table 2). The calculated synaptic density value in relation to the volume or surface of the PN profiles was close to two‐thirds of the corresponding values measured in the other two glomeruli (Table 2). In addition, the amount of recurrency (i.e., number of PN output, feedback synapses) was lower in the DM2 in comparison with the VA7 and DL5 glomeruli (ratio of output to input was 0.27:1). Most PN output synapses in this glomerulus were tetrads. Only 4% of all analyzed PN postsynaptic sites were postsynaptic to OSN‐type 2 presynaptic sites housed by OSN‐type cells (Fig. 12, Table 3). Ninety‐three percent of the input synapses onto the PN profiles were postsynaptic to unlabeled neurons that were not traced, and 7% were postsynaptic to other PN profiles.

### PN connectivity

The statistical data for PN synapses in the three studied glomeruli are summarized in Figure 13. We found invariant features of the PN microcircuitry, such as a high proportion of PN postsynaptic sites (post/µm^3^ in Fig. 13A), and an average PN recurrency, i.e., number of PN output synapses on average of about 30%. Although these data indicate a common tendency toward PN connectivity across all three glomeruli, it is noteworthy that the number of PN postsynaptic sites and the ratio of input and output synapses (i.e., a relatively low recurrency) were lower in the DM2 relative to the other two glomeruli (Fig. 13).

Our statistical data indicate that PNs and LN‐type cells were mostly postsynaptic to OSN‐type cells (Fig. 9). To reveal specific cell‐type connectivity in more detail, we performed a numerical analysis restricted to selected profiles of PN, OSN‐type, and LN‐type cells in the VA7 glomerulus, where we also performed dense reconstructions (Figs. 5, 6, 7, 8). To this end, we counted the number of postsynaptic elements or targets (Supplementary Fig. S6) as either pre‐ or postsynaptic partners of a specified cell type. We found that the output synapse from OSN‐type cells was the major synapse type (“pre” in Table S6–2 of Supplementary Fig. S6), which contacted about 66% of the identified PN profiles, followed by the LN‐type‐to‐PN synapse, which contacted about 40% of PN profiles. Surprisingly, we found a strong PN–PN connection: For example, about 17% of the synaptic sites found along the PN4 profile were presynaptic to other PN profiles. The connection between OSN‐type profiles or between OSN‐type and LN‐type profiles was relatively weak (below 5%; Supplementary Fig. S6). Because most postsynaptic profiles could not be traced, it was difficult to get a high number of contacts for the source of input onto cell profiles (90% of these contacts were nonidentified cells). The PNs received their strongest input from OSN‐type cells, and less from other PN profiles (7% vs. 2.3%), whereas LN‐type cells received the strongest input from PN cells (Supplementary Fig. S6).

### Microvolume measurements

**Figure 14:**
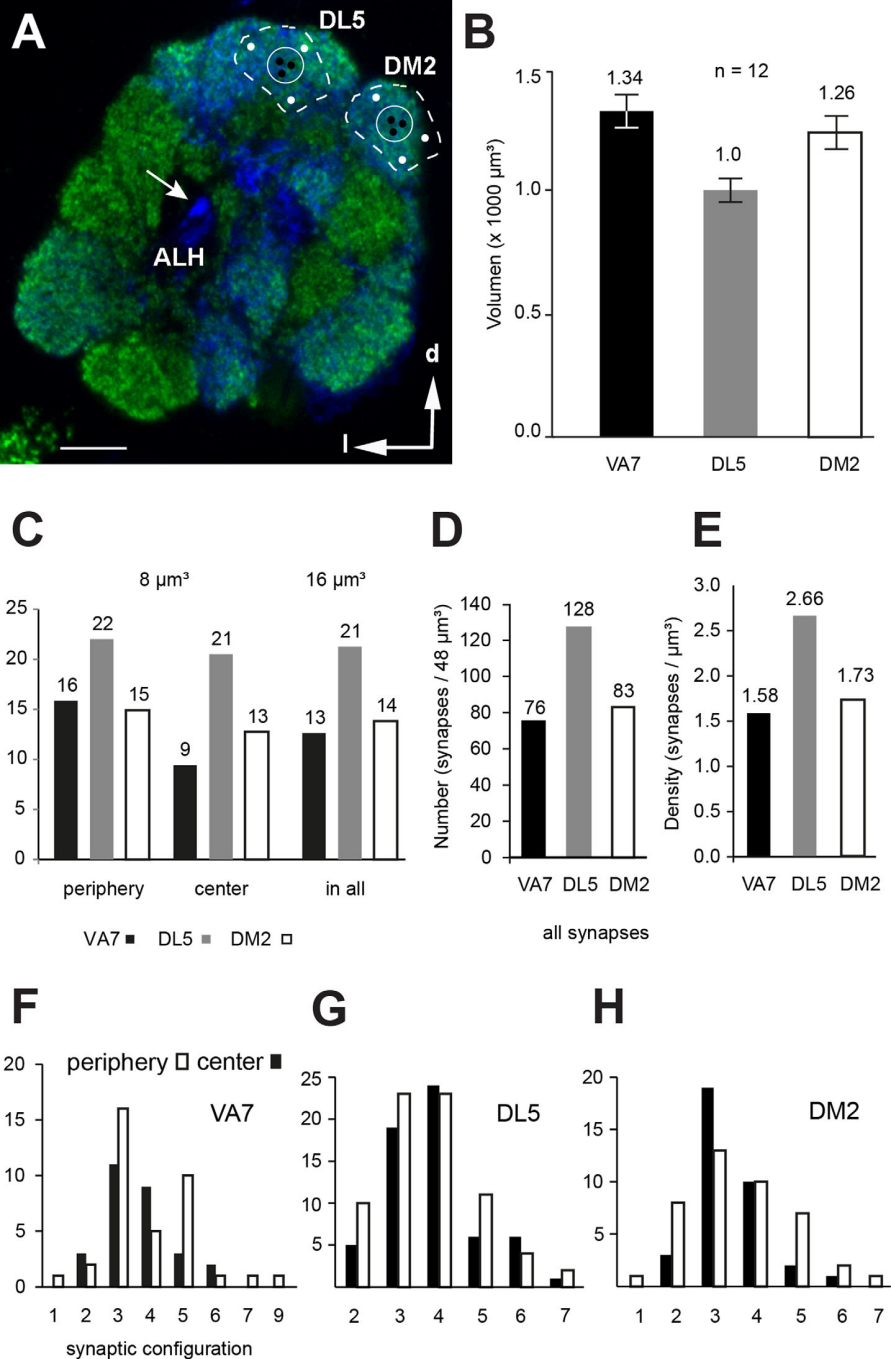
Quantitative analysis of synapse number within glomerular microvolumes. **A**: Synaptic counts of presynaptic sites (output synapses) in microvolumes (MV) located in either the center or the periphery of the three studied glomeruli (here shown for the DL5 and DM2 glomerulus, encircled). In each glomerulus six MV were chosen for the center (black dots) and the periphery (white dots). The total counting volume is 48 µm^3^ for each glomerulus, which is approximately 4–5% of the total volume of the given glomerulus. All neuronal profiles, labeled and nonlabeled, were analyzed. Arrow indicates the origin of the median antennal lobe tract, ALH, antennal hub; d, dorsal; l, lateral. Image courtesy of Y.Seki. **B**: Volume measurements of glomeruli VA7, DL5, and DM2 in males from *Drosophila melanogaster* of the left and right antennal lobe derived from confocal scans (*n* = 12 glomeruli from the left and right hemisphere in six animals). **C**–**E**: Synaptic number of presynaptic sites, for the VA7, DL5, and DM2 glomerulus, averaged from three MV counts in the periphery (each 8 µm^3^) and in the center of each glomerulus (each 8 µm^3^), respectively; “in all” indicates the average counts summed from periphery and center (volume of 16 µm^3^). **D**: Synaptic configuration for all presynapses found in all 12 microvolumes, i.e., the absolute number of synapses for all three glomeruli (each volume is 48 µm^3^). **E**: Density of synapses per cubic micrometer. **F**–**H**: Synaptic configuration of synapses in the periphery and in the center the VA7, DL5, and DM2 glomerulus; most are triad and tetrad constellations. Scale bar = 10 µm in A.

To determine the global volumetric synaptic density in a glomerulus, synaptic counts were performed in microvolumes of neuropil tissue (MV; measuring 8 µm^3^ each) sampled at three places in the periphery and three in the center in each of the three studied glomeruli, as shown for the DL5 and DM2 in Figure 14A. We thus analyzed a total volume of 48 µm^3^ in each glomerulus. Each presynaptic density was considered to represent a synapse regardless of the number of postsynaptic profiles directly opposed to it. Synapse density was lower in the center than in the periphery in all three glomeruli (Fig. 14C). Overall, the highest synapse density was found in the DL5 glomerulus (2.66 synapses/µm^3^), whereas in the DM2 and VA7 glomeruli the values were 1.73/µm^3^ and 1.58/µm^3^, respectively (Fig. 14E). Among all synapses counted within these microvolumes, triads and tetrads were the most common synaptic configuration (Fig. 14F–H).

### AL volume measurements and synaptic counts in identified glomeruli

The relative size of the olfactory system in *D. melanogaster*, including both ALs and the MBs, represents approximately 3% and 2.8%, respectively of the whole brain volume, compared with the optic lobes, which account for approximately 36% of total brain volume (Rein et al., 2002; Rybak, 2013; Ito et al., 2014). Measured as neuropil volume, the male *Drosophila* brain is smaller compared with its female counterpart: 7.11 × 10^6^/µm^3^ versus 8.11 × 10^6^/µm^3^, respectively (Shao et al., 2014).

Accordingly, we found that this relationship also holds true for AL volume, which in males was 1.19 × 10^5^ µm^3^ (±4.55 × 10^3^ µm^3^, *n* = 12 single ALs from six males; data not shown) versus 1.36 × 10^5^ µm^3^ in females. Also, the volumes of glomeruli DL5, DM2, and VA7 were slightly smaller in the male, ranging from 1.34 × 10^3^/µm^3^ for the VA7, to 1.0 × 10^3^/µm^3^ for the DL5 and 1.26 × 10^3^ µm^3^ for the DM2, i.e., on average 1.20 × 10^3^ µm^3^ (Fig. 14B). Thus, we performed our synaptic analysis within a region of the *Drosophila* brain as large as approximately 3.6 × 10^3^ µm^3^ (the volume of all three glomeruli), which corresponds to approximately 3% of a single AL and 0.042% of total brain volume.

Starting with the number of PN presynaptic sites per cubic micrometer in our microvolume (MV) measurements (1.58 sy/µm^3^ for the VA7, 2.66 sy/µm^3^ for the DL5, and 1.73 sy/µm^3^ for the DM2; Fig. 14E), and accounting for the volume of the respective male glomerulus (Fig. 14B), the total number of synapses (i.e., synaptic active sites) for each glomerulus in the male brain may be estimated, by extrapolation, to be 2660 in the DL5, 2177 in the DM2, and 2119 in the VA7.

At the TEM level, we reconstructed in the VA7 glomerulus approximately 100 µm^3^ of neurite volume for PN cells, 25 µm^3^ for OSN‐type cells, and 6 µm^3^ for LN‐type cells (Table 1). This is approximately 10% of the complete volume of a male VA7 glomerulus. In relation to the total number of synapses, we traced the VA7 glomerulus most intensively, counting 830 synapses (including pre‐ and postsynaptic sites) for all cell types, approximately 650 synapses for PN cells and considerable fewer for OSN‐type and LN‐type cells.

An estimation of the volume occupied by single PN and LN‐type arbors, or by bundle of OSN axons terminals in single glomerulus, was done after reconstruction of dendritic trees of single‐stained PN and LN cells (Seki et al., 2010), or genetically labeled OSN‐type cells (data not shown). For PN dendrites we measured approximately 180–200 µm^3^ (*n* = 5), with a maximum branching degree ranging from 13 (for the DM2–PNs) up to 23 (for DL5–PNs). In contrast, LN‐type profiles had fewer branches, and depending of the LN cell class, they had a dendritic volume of 15–20 µm^3^ for the NP 1227‐GAL4 and NP2426‐GAL lines and approximately 50 µm^3^ for the Krasavietz GAL4 lines (*n* = 15) (nomenclature of LN types according to Okada et al. [2009] and Seki et al. [2010]). For the OSN‐type cells, we estimated a volume of more than 500 µm^3^ (*n* = 2). Thus, for PN cells the synaptic count value was relatively high, and therefore constituted a good representation of PN connectivity in the VA7 glomerulus. On average, we measured the volume for a single PN profile at the TEM level as PN33–DM2 26 µm^3^, PN4–VA7 16 µm^3^, and PN1–DL5 34 µm^3^ (Supplementary Figs. S3, S7, S8; synaptic inventories).

**Figure 15:**
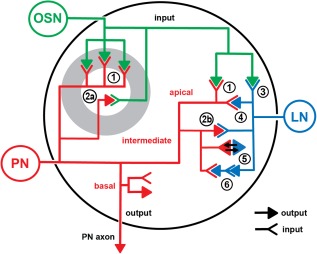
Schematic synaptic circuit of a *Drosophila* glomerulus with emphasis on the projection neuron (PN) circuit. Along the PN dendritic arborizations (PN, in red), synapses are segregated along proximal (basal), intermediate, and distal (apical) zones, according to the olfactory input and synaptic connections with other cell types: olfactory sensory neurons (OSNs) and local interneurons (LNs) within a glomerulus. The OSN bundles form several zones throughout the glomerulus, leaving OSN free zones as a core, surrounded by OSN axonal terminals (gray‐shaded area). Two of these subdomains are depicted, here, to summarize the synaptic network motifs of PN circuitry we found in our study. Predominant is the large feedforward OSN synapse onto PN with multiple spines at the PN most distal terminal endings (motif 1). PNs also form output (feedback) synapses onto OSN terminals and to LN (motif 2a and motif 2b, respectively). Further synaptic constellations are the OSN–LN synapse (motif 3), a triad configuration of OSN–LN–PN (motif 4), reciprocal PN–LN connections (motif 5), and the serial synapse LN–LN–PN (motif 6). In the most proximal (basal) portions of the PN dendritic tree, both input and output PN synapses were found.

A generic model for the connectivity of the glomeruli studied here is proposed in the following text and schematically illustrated in Figure 15. For simplicity, we refer here to the OSN‐type and LN‐type cells as being truly OSN and LN cells. In the selected volumes in which the study was done, five major connectivity modes were found: The feedforward OSN–PN synapse, the feedback PN–OSN and PN–LN synapses, and serial synapses from OSN–LN–PN and LN–LN–PN, as well as reciprocal synapses between PN and OSN and LNs, respectively.

As inferred from observations at the light microscopic level (Hummel and Zipursky, 2004), in *Drosophila* the OSN fibers penetrate the glomerulus and arborize throughout, forming spherical zones that are themselves further subdivided into dense OSN innervation and OSN‐free zones (Fig. 4D). The most prominent synapse, the OSN–PN feedforward synapse, and the PN–OSN feedback connection was found in the peripheral zones of the subglomerular zone, whereas other synaptic motifs, including the feedback PN–OSN, and PN–LN synapses, were found in more central regions of the glomerulus.

PN dendrites were organized into a proximal (or basal) zone with mostly input synapses, after their entrance to the glomerulus, an intermediate zone with mixed input and output synapses, whereas the most distal (or apical) part of the arborizations contained two domains with either mixed input/output synapses or solely input synapses (Fig. 10, 11, 15).

## DISCUSSION

A major question in understanding olfactory processing in insects is how the olfactory map established by the sensory input of OSNs is transformed, forwarded, and mapped to higher brain centers, such as the MBs bodies and LH (Heisenberg, 1998). In the model organism *D. melanogaster*, the neural network in the AL comprises the terminals of about 1,200 OSNs converging onto approximately 50 glomeruli, and two major classes of interneurons: the LNs interconnecting glomeruli, and the PNs targeting higher brain centers. The focus of this study was to map the synaptic connectivity of uniglomerular projection neurons (uPN), a class of interneurons that receive their input from OSN terminals in single AL glomeruli and convey sensory information to the central brain (Stocker, 1994; Jefferis et al., 2007; Tanaka et al., 2012).

### Genetic labeling of projection neurons

With the fly strains and methods employed here, the TEM staining mediated by the HRP transgene was restricted to the cell membranes of PNs and was interrupted at intervals, producing a “patchy” appearance. This uneven distribution of the black labeling did not impede unambiguous identification of the thinnest fibers, which would be difficult to follow in unlabeled tissue. As also reported in other studies (Schikorski et al., 2007; Li et al., 2010), HRP histochemistry leaves the ultrastructure of the labeled and nonlabeled cells relatively intact, and thus allows for the identification of cell‐specific pre‐ and postsynaptic densities, different types of vesicles, and cytoplasmic appearance.

### Ultrastructural properties of the AL and the generation of a 3D TEM model

Our observations of the AL with low‐magnification TEM confirmed previous descriptions of the general topography of this brain center in *Drosophila* based on light microscopy. In addition, we found that the tracheoles (respiratory tubes) are abundant at the AL surface, where they intermingle with axonal bundles and cell bodies, but do not enter the glomeruli. As it is well known that tracheoles do penetrate the neuropil in other regions of the nervous system, it will be important to understand whether their exclusion from olfactory synaptic neuropil has functional relevance.

Our TEM atlas will help in the recognition of the relative position and identity of tract clusters of somata and neuropil regions in future ultrastructural studies. The correlative approach, based on the combination of neuroanatomical and topographical data obtained with TEM and LSM, allowed us to identify tracts such as the anterior–posterior tract of the crepine (ap‐cre), the anterior commissure (AC), the lateral passage (LP), and the course of primary neurites of labeled PN neurons for the first time at the TEM level. Glomeruli identity could then be determined by depth in the AL and location relative to these prominent landmark structures. In conjunction with features of synaptic populations (see below), such a TEM atlas aids in the identification of distinct neuronal types purely by ultrastructural details, as has been done in two brain optic centers, the lamina ganglionaris (Meinertzhagen, 2010) and the medulla (Takemura et al., 2011, 2013; Meinertzhagen and Lee, 2012).

### Cellular adaptations of tiny brains

*Drosophila melanogaster* show features of extremely miniaturized brains, as described for parasitoid wasps (van der Woude et al., 2013). Some ultrastructural features found in this study are perhaps adaptations to accommodate functional neuronal circuits in a tiny space: sparse glial layers, extraordinarily small diameter of neurites, the aggregation of cell bodies in a rind, and polyadic synapses, the two latter being typical for insects in general (Meinertzhagen, 2010). Cellular features of the *Drosophila* AL are thus adaptations to small‐sized brains of invertebrates (Armstrong and van Hemert, 2009). From this point of view, the most prominent feature found here is perhaps the existence of a type of polyadic synapses consisting of a very elongated presynaptic density, which, in combination with the extraordinary small diameter of some fibers, made it possible for a single presynaptic density to make contact with up to 20 postsynaptic profiles. The diameter measured in PN postsynaptic profiles was very often 0.1 µm or even less, which is the lower limit for the propagation of electrical signals along a neuronal fiber (Faisal et al., 2005, cited in van der Woude et al., 2013), a dimension also reported for the optic lobes (Meinertzhagen and O'Neil, 1991). A benefit of smaller neurons over larger ones is that they are energetically less expensive both at rest and while signaling and may be packed more densely (Beutel et al., 2005; Nawroth et al., 2007; Niven and Farris, 2012).

### Identification of glomeruli

The number, size, and relative position of the approximate 50 olfactory glomeruli of each AL found in *Drosophila* are invariant and can be recognized from fly to fly (Stocker, 1994; Vosshall and Stocker, 2007; Grabe et al., 2015). In *Drosophila* the brain is approximately 10% smaller in males compared with females (Shao et al., 2014), but in spite of the existence of sexual dimorphisms among certain glomeruli, e.g., the enlarged DA1 with numerous OSNs in males (Stockinger et al., 2005), the location and shape of most glomeruli, including the ones studied here, are similar in both sexes. Indeed, the largest differences in the sex‐specific fruitless (fru+) brain morphology is found in the lateral horn (Cachero et al., 2010). Thus, no significant differences have been reported for the three glomeruli studied here. Moreover, the expression pattern of the driver used here to label PN neurons is not sexually dimorphic in the AL (Jefferis et al., 2002; Gordon and Scott, 2009).

### PNs labeled with the GH146‐Gal4 line

The AL is connected to the central brain via two major tracts carrying axons from two classes of PNs: cholinergic uniglomerular uPNs running in the mALT and GABAergic multiglomerular mPNs running in the mlALT (Tanaka et al., 2008, 2012; Ito et al., 2014). The PNs labeled with the driver used here (GH146‐GAL4) are predominantly of the uPNs class. Three major PN somata groups were labeled in our study: the anterior–dorsal cluster (adPN), the lateral cluster (lPN), and the ventral cluster (vPN), containing approximately 50, 35, and 6 neurons each. Glomerular sets, comprising 30 glomeruli, including the VA7, DM2, and DL5, are innervated by uPNs of either the adPN or lPN soma groups in a distinct and non‐overlapping manner (Jefferis et al., 2001; Marin et al., 2002; present study).

The GH146‐GAL4 line also drives expression in multiglomerular mPNs belonging to the vPN cluster. The uPNs and mPNs also differ in the arrangement of their dendritic trees in AL glomeruli. The uPNs form a thickened dendritic shaft at the entrance to the glomerulus, whereas mPN innervates the glomeruli more sparsely with thin fibers, without presynaptic specializations, and they are postsynaptic in the AL, as shown by LM studies (Okada et al., 2009; Strutz et al., 2014; Wang et al., 2014). We analyzed exclusively PNs of the uPN type and found that they have both pre‐ and postsynaptic sites.

### The median antennal lobe tract

The mALT tract constitutes the major route of AL output neurons to higher brain centers (the LH and MB), carries uPN and mPN axons, and might carry also centrifugal neurons. Shortly after exiting the AL, the uniglomerular mALT and the multiglomerular mALT axons separate and run via two pathways, the median and the mediolateral tract toward the central brain (Stocker et al., 1990; Tanaka et al., 2012; Ito et al., 2014; Strutz et al., 2014). The number of uPN axons that we identified in this study is similar to what was reported by others with other methods (Stocker et al., 1997; Marin et al., 2002). We found bundles of axons of different calibers, indicating that they belong to uPNs deriving from different cell clusters (adPN, lPN) and suggesting that they have different signal propagation features.

### Structural organization of the glomerulus

In compliance with the innervation pattern of the major cell types of the AL in insects, the glomeruli are subdivided into characteristic zones. For example, in Hymenoptera, such as honeybees, solitary bees (C. Kelber, Technical University of Darmstadt, Darmstadt, Germany, personal communication), and ants (Kelber et al., 2010; Stieb et al., 2011), the glomeruli are divided into a cap at the periphery and a core region, with sensory receptor fibers (OSNs) restricted to the cap region. In contrast, PN branches and some LN branches do not respect theses boundaries, arborizing throughout the glomerulus, whereas certain LNs ramify only in the core of the glomerulus (Flanagan and Mercer, 1989b; Abel et al., 2001; Meyer et al., 2013)

In the present study we observed that putative OSN terminals primarily invade peripheral zones, but also arborize in central areas of the glomeruli, here, still enclosing zones of noninnervated glomerular neuropil. Hence, our data confirm observations done by others with fluorescent markers and light microscopy (Hummel and Zipursky, 2004; Mosca and Luo, 2014).

LNs exhibit a high variability of morphological types with diverse, but mostly sparse branching patterns (Chou et al., 2010; Seki et al., 2010). Among them, two major morphological classes with invariant features are described. Type LN1 innervates the core of the glomerulus, and does not overlap with OSN terminals, whereas the more widely branched type LN2 innervates the periphery and the core (Sachse et al., 2007; Silbering et al. 2008; Okada et al., 2009). PNs usually innervate the entire glomerulus, in a dense and uniform fashion, in both *Drosophila* and other insects (Stocker et al., 1990; Malun, 1991a; Sun et al., 1997; Abel et al., 2001; Namiki and Kanzaki, 2008).

### Synaptic density of major cell types in the AL

We measured volumetric density of synaptic sites as a function of synapse number either per neurite volume (NV) or as the number of presynaptic sites within a microvolumen (MV) of glomerular neuropil. The advantage of the first method is that it allowed us to determine the existence of different synapse densities among different neuronal types. The second method, in contrast, made it possible to search for differences in synapse density between the periphery and the core of the glomerulus.

We found that synapse density along PN fibers, calculated per NV, was 6.9, 5.2, and 3.7 synapses/µm^3^ in the VA7, DL5, and the DM2 glomerulus, respectively. These relatively high values reflect in part the high number of PN postsynaptic sites, which to a high degree belong to the OSN–PN connection.

Using GAL4 lines for the three classes of AL neurons and presynaptic (for OSN) and postsynaptic (for PN) fluorescent markers, Mosca and Luo (2014) showed that the number of synapses made by OSN terminals, using Bruchpilot‐short (brp‐sh) as a presynaptic density marker, is constant across glomeruli at about 0.6 synapses/µm^3^. This value is much lower than what we found (3.2 OSN presynapses/µm^3^). The difference might be explained by different factors, including the fact that different glomeruli were studied, with different methods. and also perhaps possible sex‐specific differences were present (male in our study vs. female in Mosca and Luo, 2014). However, it is undoubtedly a great advance that two different approaches resulted in values of the same order of magnitude. One methodological difference is that we sampled in selected, globular parts of OSN within the glomerulus, and hence perhaps did not capture the full complement of neurite OSN volume innervation. Another aspect to be considered is the assumption that each fluorescent spot detected with confocal microscopy of brp‐sh‐fluorescent preparations (a “brp‐sh punctum”; Mosca and Luo, 2014) is always equivalent to a single active zone. This is probably correct for the visual system (Chen et al., 2014) if all or most active zones had a single “T‐bar” but might not be true for the type of elongated presynaptic densities with multiple “T‐bar” pedestals observed here with TEM. Nevertheless, the most probable explanation for our finding of higher synapse density is the much greater resolution of electron microscopy, which probably increases the chances of detecting more active synapses than with light microscopy.

A direct comparison between our estimations of total synapse numbers per glomerulus (our microvolume data [MV]) and those reported by Mosca and Luo (2014) is not possible because their study did not include the three glomeruli studied here, but their report of about 3800 synapses in the DA glomerulus is at the same order of magnitude with our estimations (between 2100 and 2600 synapses per glomerulus on average). The relative synapse load of the three major neuronal AL cell types appears to be confirmed because the OSN were responsible for the largest number of the synapses detected in DA1 (Mosca and Luo, 2014) and the three glomeruli studied here, followed by PN synapses, whereas the LN cells appear to contribute even fewer synapses to the AL circuitry (Mosca and Luo, 2014; present study).

The calculation of synapse density using data from microvolumes gave lower values because the neuropil also contain glia, which has no synapses. However, our synapse density values obtained with this method (approximately 2 synapses/µm^3^, averaged for the three glomeruli analyzed here) are well within the range reported for other neuropils across species: 1.8. synapses/µm^3^ in the *Drosophila* larval MB calyx (Cardona et al., 2010b), 2.74 in the adult calyx (Butcher et al., 2012), and 1.4 in the lamina ganglionaris, but only 0.6 in the medulla (Takemura et al., 2008; Meinertzhagen, 2010). These values are similar to those reported for vertebrates. For example, a mean density of 1.85 synapses/µm^3^ was reported for the rat hippocampus (Mishchenko, 2010) and 1–2 synapses/µm^3^ for the neocortex in mice and humans (Braitenberg and Schüz, 1991; Alonso‐Nanclares et al., 2008).

Observations from photoactivation experiments indicate that glomeruli DL5 and DM2 are innervated by two, and the VA7 by three uPNs (A. Baschwitz and V. Grabe, Max Planck Institute for Chemical Ecology, Jena, Germany, personal communication). Accordingly, we think that our analysis of PNs of the VA7 and DM2 most probably comprises profiles from more than one PN, although we cannot demonstrate this because it was not technically possible to trace all reconstructed branches to their corresponding cell bodies.

### Variation in presynaptic densities

An interesting finding of our study is that of cell‐specific features among presynaptic densities. These include the size of the platform and the number of pedestals. All presynaptic sites in PN and LN profiles, and many in OSN profiles, consisted of a platform on a pedestal but of smaller relative size in LNs relative to PNs. The largest densities were found exclusively in OSN profiles and comprised an elongated platform standing on several pedestals. These OSN‐specific elongated T‐bars are reminiscent of presynaptic sites present in the MB calyx formed by axon terminals of uPNs, which contact up to a dozen postsynaptic partners (Butcher et al., 2012). Synapses with a complex arrangement of postsynaptic elements were also reported in other insects, e.g., in the antennal lobe of the honeybee, *Apis mellifera* (Brown et al., 2002). Interestingly, a significant increment in the number of this type of elongated, multipedestal T‐bars was reported to result from a loss‐of‐function mutation in the gene *teneurin‐A* (Mosca and Luo, 2014), which encodes a protein necessary for the correct formation of synapses also in motor terminals (Hong et al., 2012; Mosca et al., 2012). Unfortunately, the method used by these authors could not discern whether this mutant phenotype reflects an increment in the number of elongated T‐bars normally occurring in OSN terminals (as shown in our study) or their appearance also in PN and/or LN neuronal branches, which normally do not form this type of presynaptic density.

### Spatial segregation of input and output synapses of PNs

All the PN profiles studied here showed differential synapse distribution along the proximal–distal axis (from thicker to thinner branches) of the dendritic tree. Spatial separation of input and output synapses along PN dendrites has been reported in two other insect species and hence appears to be a conserved feature of insect AL synaptic circuitry. In the cockroach *Periplaneta americana*, the proximal portion of the arborizations of uniglomerular PNs bears only output synapses, followed more distally by a zone with both output and input synapses and further distally, the thinnest branches bear only input synapses (Malun, 1991a, 1991b). The same distribution was reported along PN dendrites of the moth *Manduca sexta* (Sun et al., 1997; Lei et al., 2010). This appears to be somehow different in *Drosophila*, because we found instead mixed input and output synapses proximally, followed by an intermediate zone bearing only input synapses, and finally a more distal zone with mostly output synapses intermixed with input synapses or a zone of solely input synapses. Little is known about the molecular and cellular mechanisms directing the formation of different types of synapses along a single neuronal arborization, but we believe that interactions between pre‐ and postsynaptic partners, including cell adhesion proteins, cell‐to‐cell signaling, and synaptic activity are most probably involved in this interesting feature of the glomerular synaptic circuitry (Hummel and Zipursky, 2004; Hong and Luo, 2014).

Another aspect of the spatial distribution of active sites discovered here relates to their uneven distribution across the glomerulus. In addition to the accumulation of synapses at distal regions of the PN profiles associated with OSN‐type terminals, we also found uneven distribution of active sites along PN branches. In this last case, foci of many and closely located output synapses were always found in large PN boutons. This feature, combined with the existence of spatial segregation of input and output synapses, will help to define a subglomerular synaptic architecture for which evidence has already been provided by LM studies in vertebrates and invertebrates (Kasowski et al., 1999; Mosca and Luo, 2014; Pech et al., 2015).

### Synaptic circuits in identified glomeruli

We found that PN branches in *Drosophila* glomeruli are highly interconnected and make synaptic connections to all major cell types, including axodendritic connections between OSNs and PNs and reciprocal dendrodendritic connections to LNs and other PNs. Furthermore, we also found a segregation of axodendritic versus dendrodendritic zones, as reported for other insects (Boeckh and Tolbert, 1993; Sun et al., 1997; Ng et al., 2002; Lei et al., 2010) as well as for the vertebrate olfactory bulb (Didier et al., 2001; Chen and Shepherd, 2005; Linster and Cleland, 2009).

### Input processing and features of OSN synapses

The OSN–PN synapse constitutes the major input and contributes to first‐order odor processing in the AL of insects, as well as in the vertebrate olfactory bulb (Chen and Shepherd, 2005; Kazama and Wilson, 2008; Shepherd, 2011). A wiring specificity and stability between the OSN–PN synapse for PNs as well as stereotypies of the OSN–PN synapse during development have been shown (Berdnik et al., 2006; Brochtrup and Hummel, 2011). The OSN–PN synaptic input influences odor tuning by sharpening the PN response and enhancing odor discrimination, and allows for fast behavioral responses (Bhandawat et al., 2007).

Thereby, a strong convergence of sensory input onto the second‐order olfactory neuropil is characteristic for olfaction and reduces the signal‐to‐noise ratio even for weak signals (Chen and Shepherd, 2005; Galizia, 2014), allowing for detection of stimuli in high dimension odor space even at low concentrations. In the rabbit the convergence of OSN axon terminals onto each glomerulus has a ratio of 50,000:1 (Shepherd, 2011). Lower ratios have been reported for insects. In honeybees this ratio is 400:1 (Esslen and Kaissling, 1976; Flanagan and Mercer, 1989a), and in *Drosophila* it is 30:1 on average (Stocker, 2001), ranging from 8 to 60 OSN terminals per glomerulus (V. Grabe, personal communication). In *Periplaneta* and *Manduca*, the convergence ratios are quite high (1,000:1), and the most frequent synapse is a dyad (Malun, 1991a; Sun et al., 1997). In this context, it is appealing to consider that the morphology of presynaptic sites characteristic of OSN‐type 2 profiles observed here could represent an adaptation that might compensate, at least in part, for the relatively low number of OSN axons converging onto each glomerulus. PN terminals, wrapping around these OSN‐like profiles, and bearing very thin, and numerous postsynaptic spiny protrusions have high input impedance and strong postsynaptic potentials and could thus contribute to enhanced sensitivity upon odor stimuli.

A potentiation of odor‐induced responses via OSN muscarinic acetylcholine (AcH) receptors has been reported in vertebrates (Jiang et al., 2015). In *Drosophila*, muscarinic mRNAs are present in the antennal transcriptome (T. Chertemps, Pierre and Marie Curie University, Paris, France, personal communication) and have been indicated immunocytochemically (Salvaterra and Kitamoto, 2001), pointing to the existence of cholinergic receptors in *Drosophila* OSNs. In this respect, our finding of PN output contacts onto putative peptidergic cells is interesting in the context of peptidergic modulation of food‐related behavior (Winther and Ignell, 2010), and, for example, short neuropeptide F (sNPF) is colocalized in specific OSN–Ach terminals, including two of the glomeruli studied here (VA7 and DM2) (Nässel, 2014). So far, LN‐mediated presynaptic gain control of OSN (Olsen and Wilson, 2008; Ignell et al., 2009) and insulin signaling enhancing the AcH release in OSN have been reported, a circuit that could be directly controlled by PN–OSN recurrent synapses. The PN–OSN feedback synapse might provide such an excitatory, recurrent OSN input.

A further contribution for enhanced odor discrimination is chemical synapses between OSN terminals within the glomeruli. These connections might contribute to the lateral inhibition of OSNs at the sensillum level by ephaptic interactions between receptor cells targeting different glomeruli, thus enhancing contrast between opposing stimuli (Su et al., 2012). The enlarged area of the presynaptic element, including its T‐bar, will make it possible not only to signal to a larger number of postsynaptic fibers simultaneously but also to keep larger clusters of synaptic vesicles.

In summary, the OSN‐to‐PN connection represents a feedforward synapse, which would be expected for sensory receptor cells (OSN) to a PN connection (Distler and Boeckh, 1997). Its enlarged size probably gives it the ability to sustain a great deal of divergence through its many postsynaptic partners. This divergence is exceptionally high compared with other AL cell types and might serve as an amplification of sensory signal, as reported from physiological studies (Wilson, 2011). Secondly, the OSN output is also directed to nonlabeled profiles (i.e., other than PNs), suggesting simultaneous transmitter release to different cell types, and thus a possible synchrony onto the glomerular network might occur. Such synchrony of polyadic synapses could be a distinct mechanism for synaptic divergence and for synchronizing activities of postsynaptic cells, as proposed in *Caenorhabditis elegans* (Liu et al., 2007). In contrast, synchronization of PN activity for optimized odor discrimination as an interaction between PN and LN activity has been reported (Ng et al., 2002).

### PN recurrency (AL output control)

A unique property in all PN profiles examined in detail in this study is their relatively high ratio of output/input synapses. Because uPNs are cholinergic, their PN output synapses are probably excitatory. Thus, there are four sources of excitatory input into the glomerular circuitry: the OSNs, the uPNs, the multiglomerular eLNs (electrical synapses), and the PNs of the lateral antennal lobe tract (lALT) (Okada et al., 2009; Yaksi and Wilson, 2010). The ultrastructural demonstration reported here of the existence of PN recurrent synapses had been shown in *Periplaneta* (Distler et al., 1998) and *Manduca* (Sun et al., 1997) but was yet not confirmed in *Drosophila*, although it had been indicated by optical imaging (Ng et al., 2002; Pech et al., 2015) and genetic labeling (Mosca and Luo, 2014).

In *Periplaneta*, PN output synapses onto GABAergic LNs are part of an interglomerular lateral inhibitory circuitry, initiated by PN output synapses and mediated by LN GABA‐immunoreactive profiles onto other glomeruli, allowing modulation of PN output by signal contrast enhancement (Sachse and Galizia, 2002). PN odor responses have even been reported, whereby an odor excited an OSN class not targeting the PN‐specific glomerulus; these responses were very likely mediated by excitatory lateral interactions via excitatory PNs (Yaksi and Wilson, 2010).

Recurrency is also a prerequisite for the generation of oscillations within the network, which allow for the synchronization of several neurons to enhance detection of low‐concentration stimuli, for example (Stopfer et al., 1997; Tanaka et al., 2011). Within the same glomerulus, we found OSN afferent and PN recurrent input synapses onto putative LN neurons. Such a recurrent coupling between PNs and LNs is the basis for synchronization of action potentials of LN and PNs, which in turn can enhance the impact of PN discharges onto detectors, such as LNs, attuned to temporal coincidences of synaptic input (Ng et al., 2002; Tanaka et al., 2009).

The PN‐to‐PN chemical synapses found here might also contribute to the excitatory network of electrical and chemical synapses that counterbalances the inhibitory network of iLNs, thus regulating odor discrimination (Yaksi and Wilson, 2010).

In summary, the synaptic features of PNs found in this study, the existence of feedback circuitry, and the segregation of input and output synapses for a given PN appear to be general features of PN synaptic organization.

### Modulatory network of the AL (AL output control)

Electrophysiological, optical imaging, and structural studies indicate that the AL output is not simply a feedforward process arising directly from the activity pattern of OSN input. Instead, it includes modulation by inhibitory and excitatory LNs promoting presynaptic inhibition and facilitation (Shang et al., 2007; Olsen and Wilson, 2008; Root et al., 2008; Ignell et al., 2009; Seki et al., 2010), by input from higher brain centers (Hu et al., 2010) as well as feedback loops within the glomeruli (Boeckh and Tolbert, 1993; Distler and Boeckh, 1997).

The LN–OSN feedback synapse is thus part of an inhibitory network of lateral LNs (Distler et al., 1998; Olsen and Wilson, 2008; Root et al., 2008), which modulate the OSN output by presynaptic gain control, expanding the dynamic range of the PN odor response and allowing for contrast enhancement (Linster and Cleland, 2009), thus counterbalancing the excitatory network described above.

The OSN–LN–PN triad configuration is a conserved feature because it is also found in vertebrates (Kasowski et al., 1999), and reciprocal synapses between LN and PN in our study, emphasizing the participation of the LN type in reducing PN responses during habituation in an odorant‐selective manner (Das et al., 2011). Here, synaptic potentiation of the LN–PN synapse is discussed as the mechanism to decrease the PN odor response mediated by a PN recurrent‐to‐ LN pathway, i.e., retrograde signaling habituating an odor response (Das et al., 2011; Sudhakaran et al., 2012).

### Differences in glomerular microcircuitry across glomeruli

Because glomeruli are anatomically distinct and functionally specialized concerning their odor specificity as well as their contribution to the control of behavior, an interesting question is to what degree they also show particular features at the level of their synaptic microcircuitry as it can be studied with TEM serial sections.

The neural representation of odor valence has been suggested to be formed at the PN output level of the AL (Knaden et al., 2012). In particular, two clusters of glomeruli, topographically separated in the AL, show specific activation of PNs depending on stimulation with attractive or aversive odors, respectively. Weaker differences were observed when the OSN activity to the same odorant was measured in different glomeruli.

In this respect, our finding of quantitative differences between the DM2 (which responds to attractive odors) and the DL5 (which responds to aversive odors) is interesting. The DM2 has a lower absolute number of synapses relative to the DL5, and in addition has a lower ratio of output‐to‐input synapses, which is mainly due to its reduced number of recurrent output synapses. Functional studies might now address whether these structural differences are relevant for the codification of odor valence.

Support for a modulation of olfactory input at the PN level comes from Mosca and Luo (2014), who reported different number of postsynaptic densities of PNs across glomeruli. This stands in contrast to the invariant number of synapses formed by OSN types within the glomerular circuitry. To further study the differential activity of PNs, high‐ resolution optical imaging studies using pre‐ and postsynaptic markers in identified neurons (Pech et al., 2015) will help to resolve such an intrinsic PN dynamic processing.

### Outlook

Whereas this study demonstrates that serial TEM sections provide accurate data of great value for the understanding of the synaptic circuitry of the *Drosophila* AL, this approach has the disadvantage of being extremely time demanding. High‐throughput scanning EM such as focused ion beam (Knott et al., 2008) and serial block‐face scanning EM (Denk and Horstmann, 2004) have been proposed as less time‐demanding alternative methods. However, these techniques, if performed on nonspecifically labeled brain tissue, will pose a problem in identification of cellular identity, and thus of identifying circuits.

Only in restricted and well‐known circuits of the fly visual system, with a layered, homogeneous, and stereotyped organization has it been possible to employ serial TEM sections for studies of synaptic plasticity (Rybak and Meinertzhagen, 1997) and synaptic circuitry (Takemura et al., 2013) on nonlabeled material. How can we proceed to analyze larger portions of the brain in a reasonable time and with reasonable effort on the synaptic level, simultaneously taking into account the rich context of the microcircuits (neuromodulators, diverse neurotransmitter, electrical synapses) with such high complexity as found even in small invertebrate nervous systems, an analysis beyond the static analysis of the “pure” connectome (Marder, 2015)?

An alternative is genetic labeling in combination with correlative EM–LM, such as array tomography (Micheva and Smith, 2007), which allows retrospective EM (Li et al., 2010) and thus a quantification of identified synaptic populations in larger volumes of neuropil. Synaptic markers (Christiansen et al., 2011; Mosca and Luo, 2014) in combination with methods such as DenMark (Nicolaï et al., 2010) and GRASP (Feinberg et al., 2008) could be used to detect putative synaptic polarity and connections. Disadvantages of this approach, such as overexpression and accumulation of synaptic markers at nonsynaptic sites, can be overcome by targeted expression using recombinant markers (Chen et al., 2014; Frank et al., 2015).

Even though classical TEM will be necessary for verification of synaptic connections, in vivo studies on the EM level using genetic markers offer more promise in the future to study experience‐dependent plasticity (Acebes et al., 2011). Finally, functional imaging in conjunction with noninvasive in vivo tomography such as lightsheet microscopy (Ahrens et al., 2012) allows for scanning on a large scale and parallel processing in the brain.

## CONFLICT OF INTEREST STATEMENT

No authors have any known or potential conflict of interest including any financial, personal, or other relationships with other people or organizations within the years of beginning the submitted work that could inappropriately influence or be perceived to influence the work.

## ROLE OF AUTHORS

All authors had full access to all the data in the study and take responsibility for the integrity of the data and the accuracy of the data analysis. Study concept and design: RC, GT, JR, BSH. Acquisition of data: GT, SR, RC, JR. Analysis and interpretation of data: JR, GT, CA, RC, SR. Drafting of the manuscript: JR, RC. Obtained funding: RC, BSH. Critical revision of the manuscript for important intellectual content: all authors. Study supervision: RC, JR.

## Supporting information

Additional Supporting Information may be found in the online version of this article

Supporting Information Figure 1.

Supporting Information Figure 2.

Supporting Information Figure 3.

Supporting Information Figure 4.

Supporting Information Figure 5.

Supporting Information Figure 6.

Supporting Information Figure 7.

Supporting Information Figure 8.

## References

[cne23966-bib-0001] Abel R , Rybak J , Menzel R . 2001 Structure and response patterns of olfactory interneurons in the honeybee, Apis mellifera. J Comp Neurol 437:363–383. 11494262 10.1002/cne.1289

[cne23966-bib-0002] Acebes A , Martin‐Pena A , Chevalier V , Ferrus A . 2011 Synapse loss in olfactory local interneurons modifies perception. J Neurosci 31:2734–2745. 21414896 10.1523/JNEUROSCI.5046-10.2011 PMC6623785

[cne23966-bib-0003] Ache BW , Young JM . 2005 Olfaction: diverse species, conserved principles. Neuron 48:417–430. 16269360 10.1016/j.neuron.2005.10.022

[cne23966-bib-0004] Agarwal G , Isacoff E . 2011 Specializations of a pheromonal glomerulus in the Drosophila olfactory system. J Neurophysiol 105:1711–1721. 21289134 10.1152/jn.00591.2010 PMC3075298

[cne23966-bib-0005] Ahrens MB , Li JM , Orger MB , Robson DN , Schier AF , Engert F , Portugues R . 2012 Brain‐wide neuronal dynamics during motor adaptation in zebrafish. Nature 485:473–477. 10.1038/nature11057 PMC3618960 22622571

[cne23966-bib-0006] Alonso‐Nanclares L , Gonzalez‐Soriano J , Rodriguez JR , DeFelipe J . 2008 Gender differences in human cortical synaptic density. Proc Natl Acad Sci U S A 105:14615–14619. 18779570 10.1073/pnas.0803652105 PMC2567215

[cne23966-bib-0007] Armstrong JD , van Hemert JI . 2009 Towards a virtual fly brain. Philos Trans R Soc Lond B 367:2387–2397. 10.1098/rsta.2008.0308 19414461

[cne23966-bib-0008] Berdnik D , Chihara T , Couto A , Luo L . 2006 Wiring stability of the adult *Drosophila* olfactory circuit after lesion. J Neurosci 26:3367–3376. 16571743 10.1523/JNEUROSCI.4941-05.2006 PMC6673868

[cne23966-bib-0009] Beutel RG , Pohl H , Hünefeld F . 2005 Strepsipteran brains and effects of miniaturization (Insecta). Arthropod Struct Dev 34:301–313.

[cne23966-bib-0010] Bhandawat V , Olsen SR , Gouwens NW , Schlief ML , Wilson RI . 2007 Sensory processing in the *Drosophila* antennal lobe increases reliability and separability of ensemble odor representations. Nat Neurosci 10:1474–1482. 17922008 10.1038/nn1976 PMC2838615

[cne23966-bib-0011] Boeckh J , Tolbert LP . 1993 Synaptic organization and development of the antennal lobe in insects. Microsc Res Tech 24:260–280. 8431606 10.1002/jemt.1070240305

[cne23966-bib-0012] Braitenberg V , Schüz A . 1991 Anatomy of the cortex. Berlin: Springer.

[cne23966-bib-0013] Brand A , Perrimon N . 1993 Targeted gene expression as a means of altering cell fates and generating dominant phenotypes. Development 118:401–415. 8223268 10.1242/dev.118.2.401

[cne23966-bib-0014] Brochtrup A , Hummel T . 2011 Olfactory map formation in the *Drosophila* brain: genetic specificity and neuronal variability. Curr Opin Neurobiol 21:85–92. 21112768 10.1016/j.conb.2010.11.001

[cne23966-bib-0015] Brown SM , Napper RM , Thompson CM , Mercer AR . 2002 Stereological analysis reveals striking differences in the structural plasticity of two readily identifiable glomeruli in the antennal lobes of the adult worker honeybee. J Neurosci 22:8514–8522. 12351725 10.1523/JNEUROSCI.22-19-08514.2002 PMC6757800

[cne23966-bib-0016] Butcher NJ , Friedrich AB , Lu Z , Tanimoto H , Meinertzhagen IA . 2012 Different classes of input and output neurons reveal new features in microglomeruli of the adult *Drosophila* mushroom body calyx. J Comp Neurol 520:2185–2201. 22237598 10.1002/cne.23037

[cne23966-bib-0017] Cachero S , Ostrovsky AD , Yu JY , Dickson BJ , Jefferis GSXE . 2010 Sexual dimorphism in the fly brain. Curr Biol 20:1589–1601. 20832311 10.1016/j.cub.2010.07.045 PMC2957842

[cne23966-bib-0018] Cardona A , Saalfeld S , Arganda I , Pereanu W , Schindelin J , Hartenstein V . 2010a Identifying neuronal lineages of *Drosophila* by sequence analysis of axon tracts. J Neurosci 30:7538–7553. 20519528 10.1523/JNEUROSCI.0186-10.2010 PMC2905806

[cne23966-bib-0019] Cardona A , Saalfeld S , Preibisch S , Schmid B , Cheng A , Pulokas J , Tomancak P , Hartenstein V . 2010b An integrated micro‐ and macroarchitectural analysis of the *Drosophila* brain by computer‐assisted serial section electron microscopy. PLoS Biol 8:1–17. 10.1371/journal.pbio.1000502 PMC2950124 20957184

[cne23966-bib-0020] Cardona A , Saalfeld S , Schindelin J , Arganda‐Carreras I , Preibisch S , Longair M , Tomancak P , Hartenstein V , Douglas RJ . 2012 TrakEM2 Software for neural circuit reconstruction. PLoS One 7:e38011. 22723842 10.1371/journal.pone.0038011 PMC3378562

[cne23966-bib-0021] Chen WR , Shepherd GM . 2005 The olfactory glomerulus: a cortical module with specific functions. J Neurocytol 34:353–360. 16841172 10.1007/s11068-005-8362-0

[cne23966-bib-0022] Chen Y , Akin O , Nern A , Tsui CYK , Pecot Matthew Y , Zipursky SL . 2014 Cell‐type‐specific labeling of synapses in vivo through synaptic tagging with recombination. Neuron 81:280–293. 24462095 10.1016/j.neuron.2013.12.021 PMC4025979

[cne23966-bib-0023] Chiang AS , Lin CY , Chuang CC , Chang HM , Hsieh CH , Yeh CW , Shih CT , Wu JJ , Wang GT , Chen YC , Wu CC , Chen GY , Ching YT , Lee PC , Lin HH , Hsu HW , Huang YA , Chen JY , Chiang HJ , Lu CF , Ni RF , Yeh CY , Hwang JK . 2011 Three‐dimensional reconstruction of brain‐wide wiring networks in *Drosophila* at single‐cell resolution. Curr Biol 21:1–11. 21129968 10.1016/j.cub.2010.11.056

[cne23966-bib-0024] Chou YH , Spletter ML , Yaksi E , Leong JC , Wilson RI , Luo L . 2010 Diversity and wiring variability of olfactory local interneurons in the *Drosophila* antennal lobe. Nat Neurosci 13:439–449. 20139975 10.1038/nn.2489 PMC2847188

[cne23966-bib-0025] Christiansen F , Zube C , Andlauer TF , Wichmann C , Fouquet W , Owald D , Mertel S , Leiss F , Tavosanis G , Luna AJ , Fiala A , Sigrist SJ . 2011 Presynapses in Kenyon cell dendrites in the mushroom body calyx of *Drosophila* . J Neurosci 31:9696–9707. 21715635 10.1523/JNEUROSCI.6542-10.2011 PMC6623142

[cne23966-bib-0026] Couto A , Alenius M , Dickson BJ . 2005 Molecular, anatomical, and functional organization of the *Drosophila* olfactory system. Curr Biol 15:1535–1547. 16139208 10.1016/j.cub.2005.07.034

[cne23966-bib-0027] Das S , Sadanandappa MK , Dervan A , Larkin A , Lee JA , Sudhakaran IP , Priya R , Heidari Rh , Holohan EE , Pimentel A , Gandhi A , Ito K , Sanyal S , Wang JW , Rodrigues V , Ramaswami M . 2011 Plasticity of local GABAergic interneurons drives olfactory habituation. Proc Natl Acad Sci U S A 108:E646–654. 21795607 10.1073/pnas.1106411108 PMC3169145

[cne23966-bib-0028] de Bruyne M , Clyne PJ , Carlson JR . 1999 Odor coding in a model olfactory organ: the *Drosophila* maxillary palp. J Neurosci 19:4520–4532. 10341252 10.1523/JNEUROSCI.19-11-04520.1999 PMC6782632

[cne23966-bib-0029] de Bruyne M , Foster K , Carlson JR . 2001 Odor coding in the *Drosophila* antenna. Neuron 30:537–552. 11395013 10.1016/s0896-6273(01)00289-6

[cne23966-bib-0030] Denk W , Horstmann H . 2004 Scanning electron microscopy to reconstruct three‐dimensional tissue nanostructure. PLoS Biol 2:10. 10.1371/journal.pbio.0020329 PMC524270 15514700

[cne23966-bib-0031] Didier A , Carleton A , Bjaalie JG , Vincent J‐D , Ottersen OP , Storm‐Mathisen J , Lledo P‐M . 2001 A dendrodendritic reciprocal synapse provides a recurrent excitatory connection in the olfactory bulb. Proc Natl Acad Sci U S A 98:6441–6446. 11353824 10.1073/pnas.101126398 PMC33487

[cne23966-bib-0032] Distler PG , Boeckh J . 1997 Synaptic connections between identified neuron types in the antennal lobe glomeruli of the cockroach, *Periplaneta americana*: I. Uniglomerular projection neurons. J Comp Neurol 378:307–319. 9034893

[cne23966-bib-0033] Distler PG , Gruber C , Boeckh J . 1998 Synaptic connections between GABA‐immunoreactive neurons and uniglomerular projection neurons within the antennal lobe of the cockroach, *Periplaneta americana* . Synapse 29:1–13. 9552171 10.1002/(SICI)1098-2396(199805)29:1<1::AID-SYN1>3.0.CO;2-C

[cne23966-bib-0034] el Jundi B , Huetteroth W , Kurylas AE , Schachtner J . 2009 Anisometric brain dimorphism revisited: implementation of a volumetric 3D standard brain in *Manduca sexta* . J Comp Neurol 517:210–225. 19731336 10.1002/cne.22150

[cne23966-bib-0035] Esslen J , Kaissling K‐E . 1976 Zahl und Verteilung antennaler Sensillen bei der Honigbiene (*Apis mellifera* L.). Zoomorphologie 83:227–251.

[cne23966-bib-0036] Evers JF , Schmitt S , Sibila M , Duch C . 2005 Progress in functional neuroanatomy: precise automatic geometric reconstruction of neuronal morphology from confocal image stacks. J Neurophysiol 93:2331–2342. 15537815 10.1152/jn.00761.2004

[cne23966-bib-0037] Faisal AA , White JA , Laughlin SB . 2005 Ion‐channel noise places limits on the miniaturization of the brain's wiring. Curr Biol 15:1143–1149. 15964281 10.1016/j.cub.2005.05.056

[cne23966-bib-0038] Feinberg EH , VanHoven MK , Bendesky A , Wang G , Fetter RD , Shen K , Bargmann CI . 2008 GFP reconstitution across synaptic partners (GRASP) defines cell contacts and synapses in living nervous systems. Neuron 57:353–363. 18255029 10.1016/j.neuron.2007.11.030

[cne23966-bib-0039] Fishilevich E , Vosshall LB . 2005 Genetic and functional subdivision of the *Drosophila* antennal lobe. Curr Biol 15:1548–1553. 16139209 10.1016/j.cub.2005.07.066

[cne23966-bib-0040] Flanagan D , Mercer AR . 1989a An atlas and 3‐D reconstruction of the antennal lobes in the worker honey bee, *Apis mellifera* L. (Hymenoptera: Apidae). Int J Insect Morphol Embryol 18:145–159.

[cne23966-bib-0041] Flanagan D , Mercer AR . 1989b Morphology and response characteristics of neurons in the deutocerebrum of the brain in the honeybee *Apis mellifera* . J Comp Physiol A 164:483–494.

[cne23966-bib-0042] Fonta C , Sun XJ , Masson C . 1993 Morphology and spatial distribution of bee antennal lobe interneurones responsive to odours. Chem Senses 18:101–119.

[cne23966-bib-0043] Fouquet W , Owald D , Wichmann C , Mertel S , Depner H , Dyba M , Hallermann S , Kittel RJ , Eimer S , Sigrist SJ . 2009 Maturation of active zone assembly by *Drosophila* Bruchpilot. J Cell Biol 186:129–145. 19596851 10.1083/jcb.200812150 PMC2712991

[cne23966-bib-0044] Frank DD , Jouandet GC , Kearney PJ , Macpherson LJ , Gallio M . 2015 Temperature representation in the *Drosophila* brain. Nature 519:358–361. 25739506 10.1038/nature14284 PMC4554763

[cne23966-bib-0045] Fröhlich . 1985 Freeze‐Fracture Study of an Invertebrate Multiple‐Contact Synapse: The Fly Photoreceptor Tetrad. J Comp Neurol 241:311–326. 4086659 10.1002/cne.902410306

[cne23966-bib-0046] Galizia CG . 2014 Olfactory coding in the insect brain: data and conjectures. Eur J Neurosci 39:1784–1795. 24698302 10.1111/ejn.12558 PMC4237541

[cne23966-bib-0047] Galizia CG , Sachse S . 2010 Odor coding in insects In: Menini A, editor. Neurobiology of olfaction. Boca Raton, FL: CRC Press p 35–70. 21882428

[cne23966-bib-0048] Galizia CG , McIlwrath SL , Menzel R . 1999 A digital three‐dimensional atlas of the honeybee antennal lobe based on optical sections acquired by confocal microscopy. Cell Tissue Res 295:383–394. 10022959 10.1007/s004410051245

[cne23966-bib-0049] Gascuel J , Masson C . 1991 A quantitative ultrastructural‐study of the honeybee antennal lobe. Tissue Cell 23:341–355. 18621165 10.1016/0040-8166(91)90052-u

[cne23966-bib-0050] Gordon MD , Scott K . 2009 Motor control in a Drosophila taste circuit. Neuron 61:373–384. 19217375 10.1016/j.neuron.2008.12.033 PMC2650400

[cne23966-bib-0051] Grabe V , Strutz A , Baschwitz A , Hansson BS , Sachse S . 2015 Digital in vivo 3D atlas of the antennal lobe of *Drosophila melanogaster* . J Comp Neurol 523:530–544. 25327641 10.1002/cne.23697

[cne23966-bib-0052] Grosjean Y , Rytz R , Farine J‐P , Abuin L , Cortot J , Jefferis GSXE , Benton R . 2011 An olfactory receptor for food‐derived odours promotes male courtship in Drosophila. Nature 478:236–240. 21964331 10.1038/nature10428

[cne23966-bib-0053] Grosse‐Wilde E , Kuebler LS , Bucks S , Vogel H , Wicher D , Hansson BS . 2011 Antennal transcriptome of *Manduca sexta* . Proc Natl Acad Sci U S A 108:7449–7454. 21498690 10.1073/pnas.1017963108 PMC3088587

[cne23966-bib-0054] Hansson BS , Stensmyr MC . 2011 Evolution of insect olfaction. Neuron 72:698–711. 22153368 10.1016/j.neuron.2011.11.003

[cne23966-bib-0055] Hansson BS , Christensen TA , Hildebrand JG . 1991 Functionally distinct subdivisions of the macroglomerular complex in the antennal lobe of the male sphinx moth *Manduca sexta* . J Comp Neurol 312:264–278. 1748732 10.1002/cne.903120209

[cne23966-bib-0056] Hansson BS , Ljungberg H , Hallberg E , Löfstedt C . 1992 Functional specialization of olfactory glomeruli in a moth. Science 256:1313–1315. 1598574 10.1126/science.1598574

[cne23966-bib-0057] Heisenberg M . 1998 What do the mushroom bodies do for the insect brain? An introduction. Learn Mem 5:1–10. 10454369 PMC311238

[cne23966-bib-0058] Hildebrand JG , Shepherd GM . 1997 Mechanisms of olfactory discrimination: converging evidence for common principles across phyla. Annu Rev Neurosci 20:595–631. 9056726 10.1146/annurev.neuro.20.1.595

[cne23966-bib-0059] Hofbauer A , Ebel T , Waltenspiel B , Oswald P , Chen Y‐c , Halder P , Biskup S , Lewandrowski U , Winkler C , Sickmann A , Buchner S , Buchner E . 2009 The Wuerzburg Hybridoma Library against *Drosophila* Brain. J Neurogenet 23:78–91. 19132598 10.1080/01677060802471627

[cne23966-bib-0060] Hong W , Luo L . 2014 Genetic control of wiring specificity in the fly olfactory system. Genetics 196:17–29. 24395823 10.1534/genetics.113.154336 PMC3872183

[cne23966-bib-0061] Hong W , Mosca TJ , Luo L . 2012 Teneurins instruct synaptic partner matching in an olfactory map. Nature 484:201–207. 22425994 10.1038/nature10926 PMC3345284

[cne23966-bib-0062] Hu A , Zhang W , Wang Z . 2010 Functional feedback from mushroom bodies to antennal lobes in the *Drosophila* olfactory pathway. Proc Natl Acad Sci U S A 107:10262–10267. 20479249 10.1073/pnas.0914912107 PMC2890443

[cne23966-bib-0063] Hummel T , Zipursky SL . 2004 Afferent induction of olfactory glomeruli requires N‐cadherin. Neuron 42:77–88. 15066266 10.1016/s0896-6273(04)00158-8

[cne23966-bib-0064] Ibba I , Angioy A , Hansson BS , Dekker T . 2010 Macroglomeruli for fruit odors change blend preference in *Drosophila* . Naturwissenschaften 97:1059–1066. 20972770 10.1007/s00114-010-0727-2

[cne23966-bib-0065] Ignell R , Root CM , Birse RT , Wang JW , Nassel DR , Winther AM . 2009 Presynaptic peptidergic modulation of olfactory receptor neurons in *Drosophila* . Proc Natl Acad Sci U S A 106:13070–13075. 19625621 10.1073/pnas.0813004106 PMC2722350

[cne23966-bib-0066] Ito K , Shinomiya K , Ito M , Armstrong JD , Boyan G , Hartenstein V , Harzsch S , Heisenberg M , Homberg U , Jenett A , Keshishian H , Restifo Linda L , Rössler W , Simpson Julie H , Strausfeld Nicholas J , Strauss R , Vosshall Leslie B . 2014 A systematic nomenclature for the insect brain. Neuron 81:755–765. 24559671 10.1016/j.neuron.2013.12.017

[cne23966-bib-0067] Jefferis GS , Marin EC , Stocker RF , Luo L . 2001 Target neuron prespecification in the olfactory map of Drosophila. Nature 414:204–208. 11719930 10.1038/35102574

[cne23966-bib-0068] Jefferis G , Marin EC , Watts RJ , Luo LQ . 2002 Development of neuronal connectivity in Drosophila antennal lobes and mushroom bodies. Curr Opin Neurobiol 12:80–86. 11861168 10.1016/s0959-4388(02)00293-3

[cne23966-bib-0069] Jefferis GS , Potter CJ , Chan AM , Marin EC , Rohlfing T , Maurer CR Jr , Luo L . 2007 Comprehensive maps of *Drosophila* higher olfactory centers: spatially segregated fruit and pheromone representation. Cell 128:1187–1203. 17382886 10.1016/j.cell.2007.01.040 PMC1885945

[cne23966-bib-0070] Jenett A , Rubin GM , Ngo TT , Shepherd D , Murphy C , Dionne H , Pfeiffer BD , Cavallaro A , Hall D , Jeter J , Iyer N , Fetter D , Hausenfluck JH , Peng H , Trautman ET , Svirskas RR , Myers EW , Iwinski ZR , Aso Y , DePasquale GM , Enos A , Hulamm P , Lam SC , Li HH , Laverty TR , Long F , Qu L , Murphy SD , Rokicki K , Safford T , Shaw K , Simpson JH , Sowell A , Tae S , Yu Y , Zugates CT . 2012 A GAL4‐driver line resource for Drosophila neurobiology. Cell Rep 2:991–1001. 23063364 10.1016/j.celrep.2012.09.011 PMC3515021

[cne23966-bib-0071] Jiang Y , Li YR , Tian H , Ma M , Matsunami H . 2015 Muscarinic acetylcholine receptor M3 modulates odorant receptor activity via inhibition of β‐arrestin‐2 recruitment. Nat Commun 6:6448. 25800153 10.1038/ncomms7448 PMC4372811

[cne23966-bib-0072] Kasowski HJ , Kim H , Greer CA . 1999 Compartmental organization of the olfactory bulb glomerulus. J Comp Neurol 407:261–274. 10213094

[cne23966-bib-0073] Kazama H , Wilson RI . 2008 Homeostatic matching and nonlinear amplification at identified central synapses. Neuron 58:401–413. 18466750 10.1016/j.neuron.2008.02.030 PMC2429849

[cne23966-bib-0074] Kelber C , Rossler W , Kleineidam CJ . 2006 Multiple olfactory receptor neurons and their axonal projections in the antennal lobe of the honeybee *Apis mellifera* . J Comp Neurol 496:395–405. 16566001 10.1002/cne.20930

[cne23966-bib-0075] Kelber C , Rossler W , Kleineidam CJ . 2010 Phenotypic plasticity in number of glomeruli and sensory innervation of the antennal lobe in leaf‐cutting ant workers (*A. vollenweideri*). Dev Neurobiol 70:222–234. 20029932 10.1002/dneu.20782

[cne23966-bib-0076] Knaden M , Strutz A , Ahsan J , Sachse S , Hansson BS . 2012 Spatial representation of odorant valence in an insect brain. Cell Rep 1:392–399. 22832228 10.1016/j.celrep.2012.03.002

[cne23966-bib-0077] Knott G , Marchman H , Lich B . 2008 Serial section scanning electron microscopy of adult brain tissue using focused ion beam milling. J Neurosci 28:2964–2959. 10.1523/JNEUROSCI.3189-07.2008 PMC6670719 18353998

[cne23966-bib-0078] Kwon H‐W , Lu T , Rützler M , Zwiebel LJ . 2006 Olfactory responses in a gustatory organ of the malaria vector mosquito *Anopheles gambiae* . Proc Natl Acad Sci U S A 103:13526–13531. 16938890 10.1073/pnas.0601107103 PMC1569196

[cne23966-bib-0079] Laissue PP , Vosshall LB . 2008 The olfactory sensory map in *Drosophila* In: Technau GM, editor. Brain development in *Drosophila melanogaster*. New York: Springer p 102–114 10.1007/978-0-387-78261-4_7 18683641

[cne23966-bib-0080] Larsson MC , Domingos AI , Jones WD , Chiappe ME , Amrein H , Vosshall LB . 2004 Or83b encodes a broadly expressed odorant receptor essential for Drosophila olfaction. Neuron 43:703–714. 15339651 10.1016/j.neuron.2004.08.019

[cne23966-bib-0081] Lei H , Oland LA , Riffell JA , Beyerlein A , Hildebrand JG . 2010 Microcircuits for olfactory information processing in the antennal lobe of *Manduca sexta* In: Shepherd GM, Grillner S, editors. Handbook of brain microcircuits. New York: Oxford University Press p 417–426.

[cne23966-bib-0082] Leitch B , Laurent G . 1996 GABAergic synapses in the antennal lobe and mushroom body of the locust olfactory system. J Comp Neurol 372:487–514. 8876449 10.1002/(SICI)1096-9861(19960902)372:4<487::AID-CNE1>3.0.CO;2-0

[cne23966-bib-0084] Li J , Wang Y , Chiu SL , Cline HT . 2010 Membrane targeted horseradish peroxidase as a marker for correlative fluorescence and electron microscopy studies. Front Neural Circuits 4:6. 20204144 10.3389/neuro.04.006.2010 PMC2831632

[cne23966-bib-0085] Linster C , Cleland TA . 2009 Glomerular microcircuits in the olfactory bulb. Neural Netw 22:1169–1173. 19646847 10.1016/j.neunet.2009.07.013 PMC2771633

[cne23966-bib-0086] Liu KSY , Siebert M , Mertel S , Knoche E , Wegener S , Wichmann C , Matkovic T , Muhammad K , Depner H , Mettke C , Bückers J , Hell SW , Müller M , Davis GW , Schmitz D , Sigrist SJ . 2011 RIM‐binding protein, a central part of the active zone, is essential for neurotransmitter release. Science 334:1565–1569. 22174254 10.1126/science.1212991

[cne23966-bib-0087] Liu Q , Chen B , Hall DH , Wang Z‐W . 2007 A quantum of neurotransmitter causes minis in multiple postsynaptic cells at the *Caenorhabditis elegans* neuromuscular junction. Dev Neurobiol 67:123–128. 17443777 10.1002/dneu.20307

[cne23966-bib-0088] Liu WW , Wilson RI . 2013 Glutamate is an inhibitory neurotransmitter in the *Drosophila* olfactory system. Proc Natl Acad Sci U S A 110:10294–10299. 23729809 10.1073/pnas.1220560110 PMC3690841

[cne23966-bib-0089] Lofaldli BB , Kvello P , Mustaparta H . 2010 Integration of the antennal lobe glomeruli and three projection neurons in the standard brain atlas of the moth *Heliothis virescens* . Front Syst Neurosci 4:5. 20179785 10.3389/neuro.06.005.2010 PMC2826183

[cne23966-bib-0090] Malun D . 1991a Inventory and distribution of synapses of identified uniglomerular projection neurons in the antennal lobe of *Periplaneta americana* . J Comp Neurol 305:348–360. 1709183 10.1002/cne.903050215

[cne23966-bib-0091] Malun D . 1991b Synaptic relationships between GABA‐immunoreactive neurons and an identified uniglomerular projection neuron in the antennal lobe of *Periplaneta americana*—a double‐labeling electron‐microscopic study. Histochemistry 96:197–207. 1917576 10.1007/BF00271538

[cne23966-bib-0092] Marder E . 2015 Understanding brains: details, intuition, and big data. PLoS Biol 13:e1002147. 25965068 10.1371/journal.pbio.1002147 PMC4428625

[cne23966-bib-0093] Marin EC , Jefferis GS , Komiyama T , Zhu H , Luo L . 2002 Representation of the glomerular olfactory map in the Drosophila brain. Cell 109:243–255. 12007410 10.1016/s0092-8674(02)00700-6

[cne23966-bib-0094] Martin JP , Beyerlein A , Dacks AM , Reisenman CE , Riffell JA , Lei H , Hildebrand JG . 2011 The neurobiology of insect olfaction: sensory processing in a comparative context. Prog Neurobiol 95:427–447. 21963552 10.1016/j.pneurobio.2011.09.007

[cne23966-bib-0095] Meinertzhagen IA . 1996 Ultrastructure and quantification of synapses in the insect nervous system. J Neurosci Meth 69:59–73. 10.1016/S0165-0270(96)00021-0 8912936

[cne23966-bib-0096] Meinertzhagen IA . 2010 The organisation of invertebrate brains: cells, synapses and circuits. Acta Zool 91:64–71.

[cne23966-bib-0097] Meinertzhagen IA , Lee C‐H . 2012 The genetic analysis of functional connectomics in *Drosophila* . Adv Genet 80:99–151. 23084874 10.1016/B978-0-12-404742-6.00003-X PMC4251806

[cne23966-bib-0098] Meinertzhagen IA , O'Neil SD . 1991 Synaptic organization of columnar elements in the lamina of the wild type in Drosophila melanogaster. J Comp Neurol 305:232–263. 1902848 10.1002/cne.903050206

[cne23966-bib-0099] Meyer A , Galizia CG , Nawrot MP . 2013 Local interneurons and projection neurons in the antennal lobe from a spiking point of view. J Neurophysiol 110:2465–2474. 24004530 10.1152/jn.00260.2013

[cne23966-bib-0100] Micheva KD , Smith SJ . 2007 Array tomography: a new tool for imaging the molecular architecture and ultrastructure of neural circuits. Neuron 55:25–36. 17610815 10.1016/j.neuron.2007.06.014 PMC2080672

[cne23966-bib-0101] Mishchenko Y . 2010 On optical detection of densely labeled synapses in neuropil and mapping connectivity with combinatorially multiplexed fluorescent synaptic markers. PLoS One 5:e8853. 20107507 10.1371/journal.pone.0008853 PMC2809746

[cne23966-bib-0102] Mosca TJ , Luo L . 2014 Synaptic organization of the *Drosophila* antennal lobe and its regulation by the Teneurins. eLife 3:e03726. 25310239 10.7554/eLife.03726 PMC4194450

[cne23966-bib-0103] Mosca TJ , Hong W , Dani VS , Favaloro V , Luo L . 2012 Trans‐synaptic Teneurin signalling in neuromuscular synapse organization and target choice. Nature 484:237–241. 22426000 10.1038/nature10923 PMC3326183

[cne23966-bib-0104] Namiki S , Kanzaki R . 2008 Reconstructing the population activity of olfactory output neurons that innervate identifi able processing units. Front Neural Circuits 2:1. 18946541 10.3389/neuro.04.001.2008 PMC2526276

[cne23966-bib-0105] Namiki S , Haupt SS , Kazawa T , Takashima A , Ikeno H , Kanzaki R . 2009 Reconstruction of virtual neural circuits in an insect brain. Front Neurosci 3:206–213. 20011143 10.3389/neuro.01.028.2009 PMC2752316

[cne23966-bib-0106] Nässel D . 2009 Neuropeptide signaling near and far: how localized and timed is the action of neuropeptides in brain circuits? Invertebr Neurosci 9:57–75. 10.1007/s10158-009-0090-1 19756790

[cne23966-bib-0107] Nässel D . 2014 Neuropeptides regulating Drosophila behavior In: Dubnau J, editor. Behavioral genetics of the fly (*Drosophila melanogaster*). Cambridge: Cambridge University Press p 20–36.

[cne23966-bib-0108] Nawroth JC , Greer CA , Chen WR , Laughlin SB , Shepherd GM . 2007 An energy budget for the olfactory glomerulus. J Neurosci 27:9790–9800. 17804639 10.1523/JNEUROSCI.1415-07.2007 PMC6672954

[cne23966-bib-0109] Ng M , Roorda RD , Lima SQ , Zemelman BV , Morcillo P , Miesenböck G . 2002 Transmission of olfactory information between three populations of neurons in the antennal lobe of the fly. Neuron 36:463–474. 12408848 10.1016/s0896-6273(02)00975-3

[cne23966-bib-0110] Nicolaï LJJ , Ramaekers A , Raemaekers T , Drozdzecki A , Mauss AS , Yan J , Landgraf M , Annaert W , Hassan BA . 2010 Genetically encoded dendritic marker sheds light on neuronal connectivity in Drosophila. Proc Natl Acad Sci U S A 107:20553–20558. 21059961 10.1073/pnas.1010198107 PMC2996714

[cne23966-bib-0111] Nishino H , Nishikawa M , Mizunami M , Yokohari F . 2009 Functional and topographic segregation of glomeruli revealed by local staining of antennal sensory neurons in the honeybee *Apis mellifera* . J Comp Neurol 515:161–180. 19412930 10.1002/cne.22064

[cne23966-bib-0112] Niven JE , Farris SM . 2012 Miniaturization of nervous systems and neurons. Curr Biol 22:R323–R329. 22575474 10.1016/j.cub.2012.04.002

[cne23966-bib-0113] Okada R , Awasaki T , Ito K . 2009 Gamma‐aminobutyric acid (GABA)‐mediated neural connections in the *Drosophila* antennal lobe. J Comp Neurol 514:74–91. 19260068 10.1002/cne.21971

[cne23966-bib-0083] Oland LA , Tolbert LP . 2003 Key interactions between neurons and glial cells during neuronal development in insects. Annu Rev Entomol 48:89–110. 12194908 10.1146/annurev.ento.48.091801.112654

[cne23966-bib-0114] Olsen SR , Wilson RI . 2008 Lateral presynaptic inhibition mediates gain control in an olfactory circuit. Nature 452:956–960. 18344978 10.1038/nature06864 PMC2824883

[cne23966-bib-0115] Pech U , Revelo Natalia H , Seitz Katharina J , Rizzoli Silvio O , Fiala A . 2015 Optical dissection of experience‐dependent pre‐ and postsynaptic plasticity in the Drosophila brain. Cell Rep 10:2083–2095. 25818295 10.1016/j.celrep.2015.02.065

[cne23966-bib-0116] Pelz D , Roeske T , Syed Z , Bruyne Md , Galizia CG . 2006 The molecular receptive range of an olfactory receptor in vivo (*Drosophila melanogaste*r Or22a). J Neurobiol 66:1544–1563. 17103386 10.1002/neu.20333

[cne23966-bib-0117] Rein K , Zockler M , Mader MT , Grübel C , Heisenberg M . 2002 The *Drosophila* standard brain. Curr Biol 12:227–231. 11839276 10.1016/s0960-9822(02)00656-5

[cne23966-bib-0118] Root CM , Masuyama K , Green DS , Enell LE , Nassel DR , Lee CH , Wang JW . 2008 A presynaptic gain control mechanism fine‐tunes olfactory behavior. Neuron 59:311–321. 18667158 10.1016/j.neuron.2008.07.003 PMC2539065

[cne23966-bib-0119] Rospars JP . 1988 Structure and development of the insect antennodeutocerebral system. Int J Insect Morphol Embryol 17:243–294.

[cne23966-bib-0120] Ruthensteiner B , Hess M . 2008 Embedding 3D models of biological specimens in PDF publications. Microsc Res Tech 71:778–786. 18785246 10.1002/jemt.20618

[cne23966-bib-0121] Rybak J . 2013 Exploring brain connectivity in insect model systems of learning and memory In: Menzel R, Benjamin P, editors. Invertebrate learning and memory. San Diego, CA: Academic Press p 26–40.

[cne23966-bib-0122] Rybak J , Meinertzhagen IA . 1997 The effects of light reversals on photoreceptor synaptogenesis in the fly *Musca domestica* . Eur J Neurosci 9:319–333. 9058052 10.1111/j.1460-9568.1997.tb01402.x

[cne23966-bib-0123] Rybak J , Kuss A , Lamecker H , Zachow S , Hege HC , Lienhard M , Singer J , Neubert K , Menzel R . 2010 The digital bee brain: integrating and managing neurons in a common 3D reference system. Front Syst Neurosci 4:30. 20827403 10.3389/fnsys.2010.00030 PMC2935790

[cne23966-bib-0124] Sachse S , Galizia CG . 2002 Role of inhibition for temporal and spatial odor representation in olfactory output neurons: a calcium imaging study. J Neurophysiol 87:1106–1117. 11826074 10.1152/jn.00325.2001

[cne23966-bib-0125] Sachse S , Rueckert E , Keller A , Okada R , Tanaka NK , Ito K , Voshall LB . 2007 Activity‐dependent plasticity in an olfactory circuit. Neuron 56:838–850. 18054860 10.1016/j.neuron.2007.10.035

[cne23966-bib-0126] Salecker I , Distler P . 1990 Serotonin‐immunoreactive neurons in the antennal lobes of the american cockroach *Periplaneta americana*—light‐microscopic and electron‐microscopic observations. Histochemistry 94:463–473. 2283309 10.1007/BF00272608

[cne23966-bib-0127] Salvaterra PM , Kitamoto T . 2001 Drosophila cholinergic neurons and processes visualized with Gal4/UAS–GFP?. Gene Expr Patterns 1:73–82. 10.1016/s1567-133x(01)00011-4 15018821

[cne23966-bib-0128] Schikorski T , Young Jr SM , Hu Y . 2007 Horseradish peroxidase cDNA as a marker for electron microscopy in neurons. J Neurosci Methods 165:210–215. 17631969 10.1016/j.jneumeth.2007.06.004

[cne23966-bib-0129] Seki Y , Rybak J , Wicher D , Sachse S , Hansson BS . 2010 Physiological and morphological characterization of local interneurons in the *Drosophila* antennal lobe. J Neurophysiol 104:1007–1019. 20505124 10.1152/jn.00249.2010

[cne23966-bib-0130] Shang Y , Claridge‐Chang A , Sjulson L , Pypaert M , Miesenböck G . 2007 Excitatory local circuits and their implications for olfactory processing in the fly antennal lobe. Cell 128:601–612. 17289577 10.1016/j.cell.2006.12.034 PMC2866183

[cne23966-bib-0131] Shao HC , Wu CC , Chen GY , Chang HM , Chiang AS , Chen YC . 2014 Developing a stereotypical Drosophila brain atlas. IEEE Trans Biomed Eng 61:2848–2858. 24960421 10.1109/TBME.2014.2332175

[cne23966-bib-0132] Shepherd GM . 2011 The olfactory bulb: a simple system in the mammalian brain. comprehensive physiology. Hoboken, NJ: John Wiley & Sons.

[cne23966-bib-0133] Shinomiya K , Matsuda K , Oishi T , Otsuna H , Ito K . 2011 Flybrain neuron database: a comprehensive database system of the Drosophila brain neurons. J Comp Neurol 519:807–833. 21280038 10.1002/cne.22540

[cne23966-bib-0134] Silbering AF , Okada R , Ito K , Galizia CG . 2008 Olfactory information processing in the *Drosophila* antennal lobe: anything goes? J Neurosci 28:13075–13087. 19052198 10.1523/JNEUROSCI.2973-08.2008 PMC6671615

[cne23966-bib-0135] Silbering AF , Rytz R , Grosjean Y , Abuin L , Ramdya P , Jefferis GS , Benton R . 2011 Complementary function and integrated wiring of the evolutionarily distinct *Drosophila* olfactory subsystems. J Neurosci 31:13357–13375. 21940430 10.1523/JNEUROSCI.2360-11.2011 PMC6623294

[cne23966-bib-0136] Stensmyr MC , Giordano E , Balloi A , Angioy A‐M , Hansson BS . 2003 Novel natural ligands for *Drosophila* olfactory receptor neurones. J Exp Biol 206:715–724. 12517989 10.1242/jeb.00143

[cne23966-bib-0137] Stensmyr MC , Dweck HK , Farhan A , Ibba I , Strutz A , Mukunda L , Linz J , Grabe V , Steck K , Lavista‐Llanos S , Wicher D , Sachse S , Knaden M , Becher PG , Seki Y , Hansson BS . 2012 A conserved dedicated olfactory circuit for detecting harmful microbes in *Drosophila* . Cell 151:1345–1357. 23217715 10.1016/j.cell.2012.09.046

[cne23966-bib-0138] Stieb SM , Kelber C , Wehner R , Rössler W . 2011 Antennal‐lobe organization in desert ants of the genus *Cataglyphis* . Brain Behav Evol 77:136–146. 21502750 10.1159/000326211

[cne23966-bib-0139] Stocker RF . 1994 The organization of the chemosensory system in *Drosophila melanogaster*: a review. Cell Tissue Res 275:3–26. 8118845 10.1007/BF00305372

[cne23966-bib-0140] Stocker RF . 2001 *Drosophila* as a focus in olfactory research: mapping of olfactory sensilla by fine structure, odor specificity, odorant receptor expression, and central connectivity. Microsc Res Tech 55:284–296. 11754508 10.1002/jemt.1178

[cne23966-bib-0141] Stocker RF , Lienhard MC , Borst A , Fischbach KF . 1990 Neuronal architecture of the antennal lobe in *Drosophila melanogaster* . Cell Tissue Res 262:9–34. 2124174 10.1007/BF00327741

[cne23966-bib-0142] Stocker RF , Heimbeck G , Gendre N , De Belle JS . 1997 Neuroblast ablation in Drosophila P(GAL4) lines reveals origins of olfactory interneurons. J Neurobiol 32:443–456. 9110257 10.1002/(sici)1097-4695(199705)32:5<443::aid-neu1>3.0.co;2-5

[cne23966-bib-0143] Stockinger P , Kvitsiani D , Rotkopf S , Tirian L , Dickson BJ . 2005 Neural circuitry that governs *Drosophila* male courtship behavior. Cell 121:795–807. 15935765 10.1016/j.cell.2005.04.026

[cne23966-bib-0144] Stopfer M , Bhagavan S , Smith BH , Laurent G . 1997 Impaired odour discrimination on desynchronization of odour‐encoding neural assemblies. Nature 390:70–74. 9363891 10.1038/36335

[cne23966-bib-0145] Stork T , Engelen D , Krudewig A , Silies M , Bainton RJ , Klämbt C . 2008 Organization and function of the blood–brain barrier in *Drosophila* . J Neurosci 28:587–597. 18199760 10.1523/JNEUROSCI.4367-07.2008 PMC6670337

[cne23966-bib-0146] Strutz A , Soelter J , Baschwitz A , Farhan A , Grabe V , Rybak J , Knaden M , Schmuker M , Hansson BS , Sachse S . 2014 Decoding odor quality and intensity in the Drosophila brain. eLife 3:e04147. 25512254 10.7554/eLife.04147 PMC4270039

[cne23966-bib-0147] Su C‐Y , Menuz K , Reisert J , Carlson JR . 2012 Non‐synaptic inhibition between grouped neurons in an olfactory circuit. Nature 492:66–71. 23172146 10.1038/nature11712 PMC3518700

[cne23966-bib-0148] Sudhakaran IP , Holohan EE , Osman S , Rodrigues V , Vijayraghavan K , Ramaswami M . 2012 Plasticity of recurrent inhibition in the *Drosophila* antennal lobe. J Neurosci 32:7225–7231. 22623667 10.1523/JNEUROSCI.1099-12.2012 PMC6622292

[cne23966-bib-0149] Suh GSB , Wong AM , Hergarden AC , Wang JW , Simon AF , Benzer S , Axel R , Anderson DJ . 2004 A single population of olfactory sensory neurons mediates an innate avoidance behaviour in *Drosophila* . Nature 431:854–859. 15372051 10.1038/nature02980

[cne23966-bib-0150] Sun XJ , Tolbert LP , Hildebrand JG . 1997 Synaptic organization of the uniglomerular projection neurons of the antennal lobe of the moth *Manduca sexta*: a laser scanning confocal and electron microscopic study. J Comp Neurol 379:2–20. 9057110 10.1002/(sici)1096-9861(19970303)379:1<2::aid-cne2>3.0.co;2-8

[cne23966-bib-0151] Takemura S‐Y , Lu Z , Meinertzhagen IA . 2008 Synaptic circuits of the Drosophila optic lobe: the input terminals to the medulla. J Comp Neurol 509:493–513. 18537121 10.1002/cne.21757 PMC2481516

[cne23966-bib-0152] Takemura S‐Y , Karuppudurai T , Ting C‐Y , Lu Z , Lee C‐H , Meinertzhagen Ian A . 2011 Cholinergic circuits integrate neighboring visual signals in a Drosophila motion detection pathway. Curr Biol 21:2077–2084. 22137471 10.1016/j.cub.2011.10.053 PMC3265035

[cne23966-bib-0153] Takemura S‐Y , Bharioke A , Lu Z , Nern A , Vitaladevuni S , Rivlin PK , Katz WT , Olbris DJ , Plaza SM , Winston P , Zhao T , Horne JA , Fetter RD , Takemura S , Blazek K , Chang L‐A , Ogundeyi O , Saunders MA , Shapiro V , Sigmund C , Rubin GM , Scheffer LK , Meinertzhagen IA , Chklovskii DB . 2013 A visual motion detection circuit suggested by *Drosophila* connectomics. Nature 500:175–181. 23925240 10.1038/nature12450 PMC3799980

[cne23966-bib-0154] Tanaka NK , Tanimoto H , Ito K . 2008 Neuronal assemblies of the Drosophila mushroom body. J Comp Neurol 508:711–755. 18395827 10.1002/cne.21692

[cne23966-bib-0155] Tanaka NK , Ito K , Stopfer M . 2009 Odor‐evoked neural oscillations in *Drosophila* are mediated by widely branching interneurons. J Neurosci 29:8595–8603. 19571150 10.1523/JNEUROSCI.1455-09.2009 PMC2753235

[cne23966-bib-0156] Tanaka NK , Dye L , Stopfer M . 2011 Dual‐labeling method for electron microscopy to characterize synaptic connectivity using genetically encoded fluorescent reporters in *Drosophila* . J Neurosci Methods 194:312–315. 21074556 10.1016/j.jneumeth.2010.10.020 PMC3062934

[cne23966-bib-0157] Tanaka NK , Endo K , Ito K . 2012 The organization of antennal lobe‐associated neurons in the adult *Drosophila* melanogaster brain. J Comp Neurol 520:4067–4130. 22592945 10.1002/cne.23142

[cne23966-bib-0158] Tian L , Hires SA , Mao T , Huber D , Chiappe ME , Chalasani SH , Petreanu L , Akerboom J , McKinney SA , Schreiter ER , Bargmann CI , Jayaraman V , Svoboda K , Looger LL . 2009 Imaging neural activity in worms, flies and mice with improved GCaMP calcium indicators. Nat Methods 6:875–881. 19898485 10.1038/nmeth.1398 PMC2858873

[cne23966-bib-0159] Trujillo‐Cenoz O. 1969 Some Aspects of the Structural Organization of the Medulla in Muscoid Flies l. J Ultrastruc Res 27:533–553. 10.1016/s0022-5320(69)80048-1 5803345

[cne23966-bib-0160] van der Woude E , Smid HM , Chittka L , Huigens ME . 2013 Breaking Haller's rule: brain‐body size isometry in a minute parasitic wasp. Brain Behav Evol 81:86–92. 23363733 10.1159/000345945

[cne23966-bib-0161] Vosshall LB , Stocker RF . 2007 Molecular architecture of smell and taste in *Drosophila* . Annu Rev Neurosci 30:505–533. 17506643 10.1146/annurev.neuro.30.051606.094306

[cne23966-bib-0162] Vosshall LB , Wong AM , Axel R . 2000 An olfactory sensory map in the fly brain. Cell 102:147–159. 10943836 10.1016/s0092-8674(00)00021-0

[cne23966-bib-0163] Wagh D , Rasse T , Asan E , Hofbauer A , Schwenkert I , Durrbeck H , Buchner S , Dabauvalle M , Schmidt M , Qin G , Wichmann C , Kittel R , Sigrist S , Buchner E . 2006 Bruchpilot, a protein with homology to ELKS/CAST, is required for structural integrity and function of synaptic active zones in Drosophila. Neuron 49:833–844. 16543132 10.1016/j.neuron.2006.02.008

[cne23966-bib-0164] Wang K , Gong J , Wang Q , Li H , Cheng Q , Liu Y , Zeng S , Wang Z . 2014 Parallel pathways convey olfactory information with opposite polarities in Drosophila. Proc Natl Acad Sci U S A 111:3164–3169. 24516124 10.1073/pnas.1317911111 PMC3939862

[cne23966-bib-0165] Watts RJ , Schuldiner O , Perrino J , Larsen C , Luo L . 2004 Glia engulf degenerating axons during developmental axon pruning. Curr Biol 14:678–684. 15084282 10.1016/j.cub.2004.03.035

[cne23966-bib-0166] Wilson RI . 2011 Understanding the functional consequences of synaptic specialization: insight from the Drosophila antennal lobe. Curr Opin Neurobiol 21:254–260. 21441021 10.1016/j.conb.2011.03.002 PMC3092845

[cne23966-bib-0167] Wilson RI , Laurent G . 2005 Role of GABAergic inhibition in shaping odor‐evoked spatiotemporal patterns in the Drosophila antennal lobe. J Neurosci 25:9069–9079. 16207866 10.1523/JNEUROSCI.2070-05.2005 PMC6725763

[cne23966-bib-0168] Winther ÅME , Ignell R . 2010 Local peptidergic signaling in the antennal lobe shapes olfactory behavior. Fly 4:167–171. 20224300 10.4161/fly.4.2.11467

[cne23966-bib-0169] Yaksi E , Wilson RI . 2010 Electrical coupling between olfactory glomeruli. Neuron 67:1034–1047. 20869599 10.1016/j.neuron.2010.08.041 PMC2954501

[cne23966-bib-0170] Yuste R , Tank DW . 1996 Dendritic integration in mammalian neurons, a century after Cajal. Neuron 16:701–716. 8607989 10.1016/s0896-6273(00)80091-4

