# Supplementary figures and images for: Synaptic circuitry of identified neurons in the antennal lobe of Drosophila melanogaster
Source: J Comp Neurol. 2016 Mar 9;524(9):1920–56. doi: 10.1002/cne.23966 (PMC6680330; doi:10.1002/cne.23966)

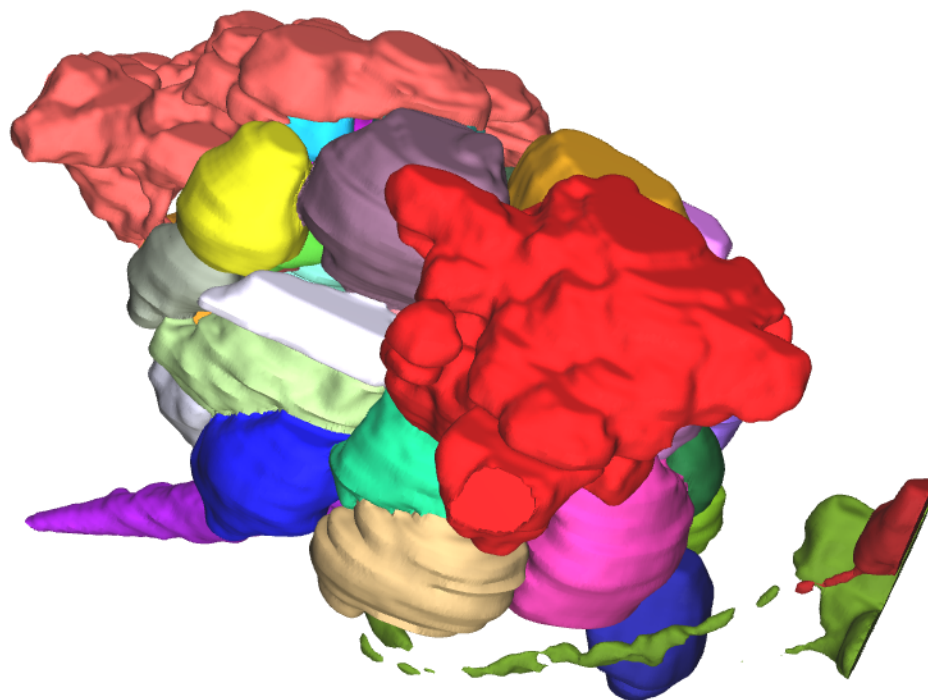

Supplement: Supplementary file 1 — Supporting Information Figure 1. [file CNE-524-1920-s001.pdf]

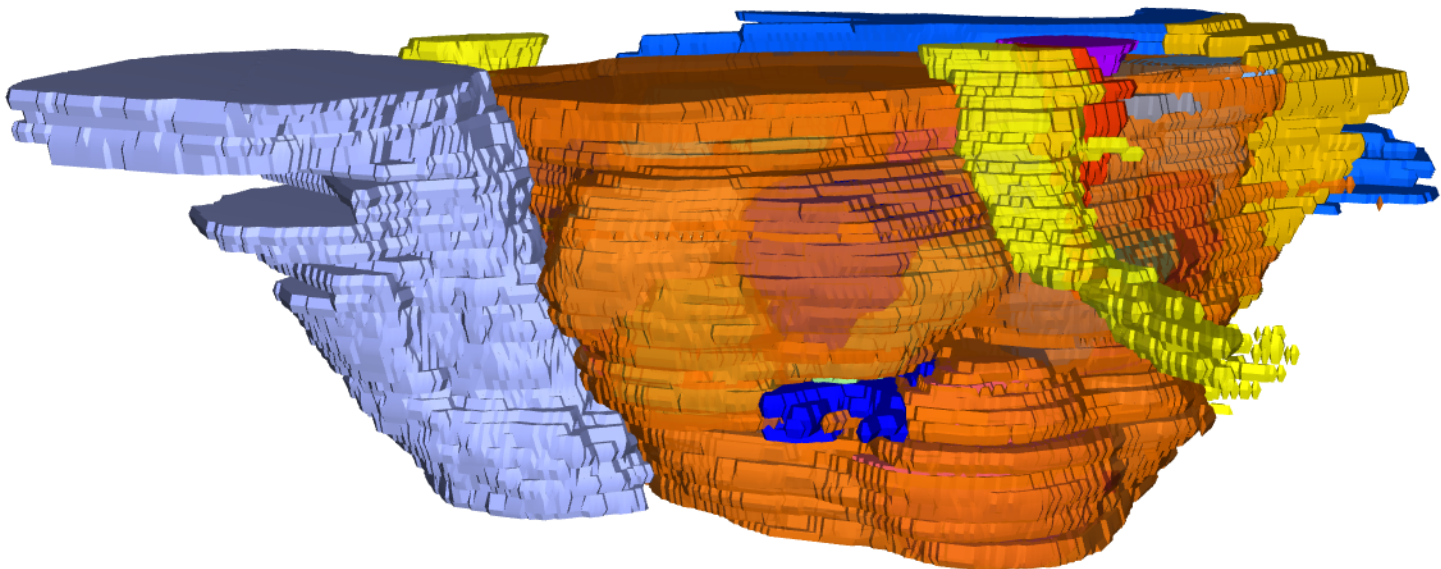

Supplement: Supplementary file 2 — Supporting Information Figure 2. [file CNE-524-1920-s002.pdf]
