# Supplementary material for: Synaptic circuitry of identified neurons in the antennal lobe of Drosophila melanogaster
Source: J Comp Neurol. 2016 Mar 9;524(9):1920–56. doi: 10.1002/cne.23966 (PMC6680330; doi:10.1002/cne.23966)
Supplement: Supplementary file 3 — Supporting Information Figure 3. [file CNE-524-1920-s003.pdf]

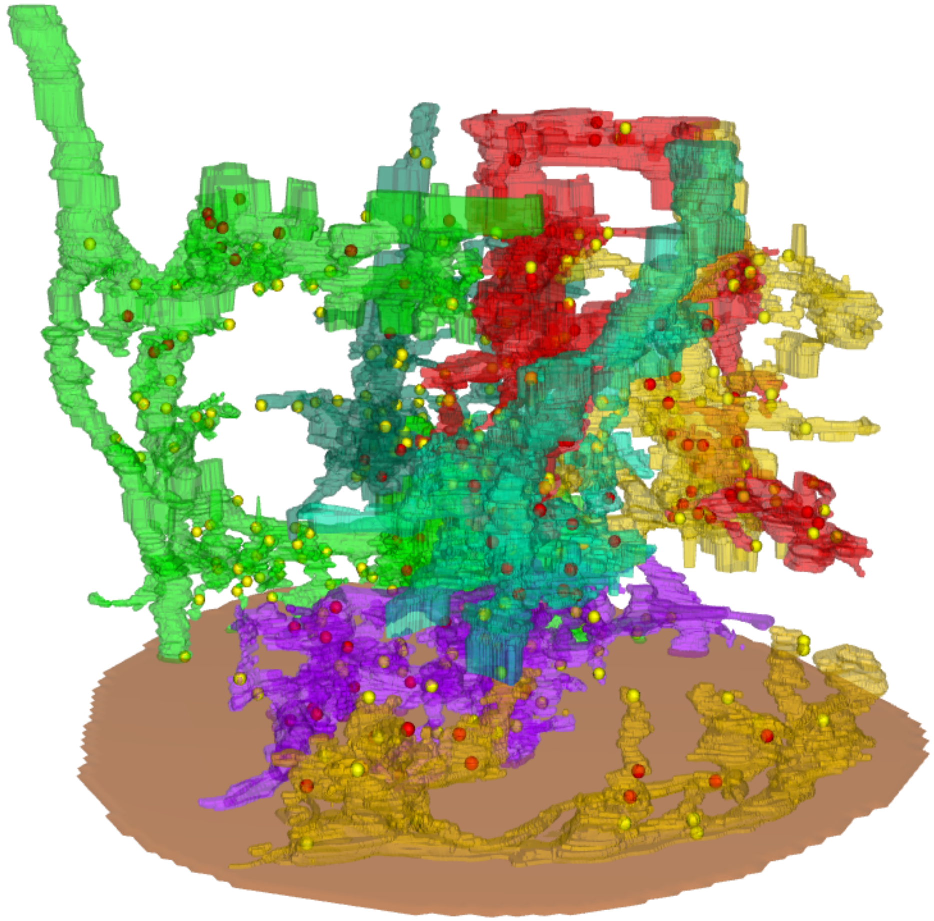

Table S3-1 VA7-PN synaptic inventory

	PN	vol (μm^3)	surf (μm^2)	pre	post	total	ratio	sy/μm^3	pre	post	sy/μm^2	pre	post	id
1	PN1a	4.63	44.90	9	14	23	0.64	4.75	1.94	3.02	0.51	0.20	0.31	535
2	PN1b	3.29	38.88	9	14	23	0.64	6.99	2.74	4.26	0.59	0.23	0.36	541
3	PN1c	7.29	56.30	4	10	14	0.40	1.92	0.55	1.37	0.25	0.07	0.18	495
4	PN2a	4.23	44.61	4	16	20	0.25	4.73	0.95	3.78	0.45	0.09	0.36	537
5	PN3	4.85	59.12	11	20	31	0.55	6.39	2.27	4.12	0.52	0.19	0.34	492
6	PN4	15.56	161.89	30	82	112	0.37	7.20	1.93	5.27	0.69	0.19	0.51	496
7	PN4a	6.27	61.26	15	17	32	0.88	5.10	2.39	2.71	0.52	0.24	0.28	1642
8	PN5	5.04	63.26	9	48	57	0.19	11.31	1.79	9.52	0.90	0.14	0.76	2220
9	PN5a	6.69	65.31	16	28	44	0.57	6.58	2.39	4.19	0.67	0.24	0.43	1132
10	PN5(c)	2.02	27.18	5	30	35	0.17	17.33	2.48	14.85	1.29	0.18	1.10	494
11	PN7	9.40	104.37	16	59	75	0.27	7.98	1.70	6.28	0.72	0.15	0.57	1650
12	PN7c	7.56	64.81	2	15	17	0.13	2.25	0.26	1.98	0.26	0.03	0.23	497
13	PN9	3.84	41.14	13	20	33	0.65	8.59	3.39	5.21	0.80	0.32	0.49	558
14	PN13	8.07	93.97	16	48	64	0.33	7.93	1.98	5.95	0.68	0.17	0.51	1506
15	PN14	1.27	16.04	1	20	21	0.05	16.54	0.79	15.75	1.31	0.06	1.25	1978
16	PN16	3.13	36.60	1	43	44	0.02	14.06	0.32	13.74	1.20	0.03	1.17	2480
17	PN17	2.07	22.05	4	10	14	0.40	1.93	4.83	0.18	0.63	0.18	2.21	644
	**total**	95.21	1001.69	165	494	659	0.33	6.92	1.73	5.19	0.66	0.16	0.49	

vol: neurite volume; surf: neurite surface; total: number of all synapses counted per profile; pre: presynaptic site (output synapse) post: postsynaptic site (input synapse); ratio: number of out-to-input synapses; sy: synapse; sy/μm^3: volumetric density ;sy/μm^2: surface density

Table S3-2   VA7-PN synaptic configuration

config	PN1a	PN1b	PN1c	PN2a	PN3	PN4a	PN4	PN5	PN5a	PN5(c)	PN7	PN7c	PN9	PN13	PN14	PN16	PN17		all PN	percent	sum	percent
	pre	pre	pre	pre	pre	pre	pre	pre	pre	pre	pre	pre	pre	pre	pre	pre	pre				targets	
3	2	1	2		2	4	7	2	7		3		2	1					33	20.5%	99	15.4%
4	5	8	1	3	7	10	13	6	8	5	10		11	10	1		2		100	62.1%	400	62.1%
5	2		1		1		8	1	1		3		1	4			1		23	14.3%	115	17.9%
6				1		1	2										1		5	3.1%	30	4.7%
																		**total**	**161**	**100.0%**	**644**	**100.0%**
config	PN1a	PN1b	PN1c	PN2a	PN3	PN4a	PN4	PN5	PN5a	PN5(c)	PN7	PN7c	PN9	PN13	PN14	PN16	PN17					
	post	post	post	post	post	post	post	post	post	post	post	post	post	post	post	post	post					
3						7	19				13			10					49	22.8%	147	13.7%
4	2		2			6	28			2	18			12		2			72	33.5%	288	26.9%
5		2				3	15				14			12					46	21.4%	230	21.5%
6							9			2	4			2		1			18	8.4%	108	10.1%
7							3			1	1			2		1			8	3.7%	56	5.2%
8	1	1					1		1		1					2			7	3.3%	56	5.2%
9				1	1		1			1						1			5	2.3%	45	4.2%
10							2												2	0.9%	20	1.9%
11																1			1	0.5%	11	1.0%
12																1			1	0.5%	12	1.1%
13																			0	0.0%	0	0.0%
14														2					2	0.9%	28	2.6%
15							1	1											2	0.9%	30	2.8%
20							1								1				2	0.9%	40	3.7%
																		**total**	**215**	**100.0%**	**1071**	**100.0%**
																		< 7	185	86.0%	773	72.2%
																		> 6	30	14.0%	298	27.8%

config		PN4			
3		21	16.8%	63	11.6%
4		52	41.6%	208	38.3%
5		40	32.0%	200	36.8%
6		12	9.6%	72	13.3%
	**total**	125	100.0%	543	100.0%

config: synaptic configuration, e.g. 4 = tetrad, **total:** number of configurations; **sum targets**: number of all postsynaptic profiles targeted by output synapses
