# Supplementary material for: Synaptic circuitry of identified neurons in the antennal lobe of Drosophila melanogaster
Source: J Comp Neurol. 2016 Mar 9;524(9):1920–56. doi: 10.1002/cne.23966 (PMC6680330; doi:10.1002/cne.23966)
Supplement: Supplementary file 4 — Supporting Information Figure 4. [file CNE-524-1920-s004.pdf]

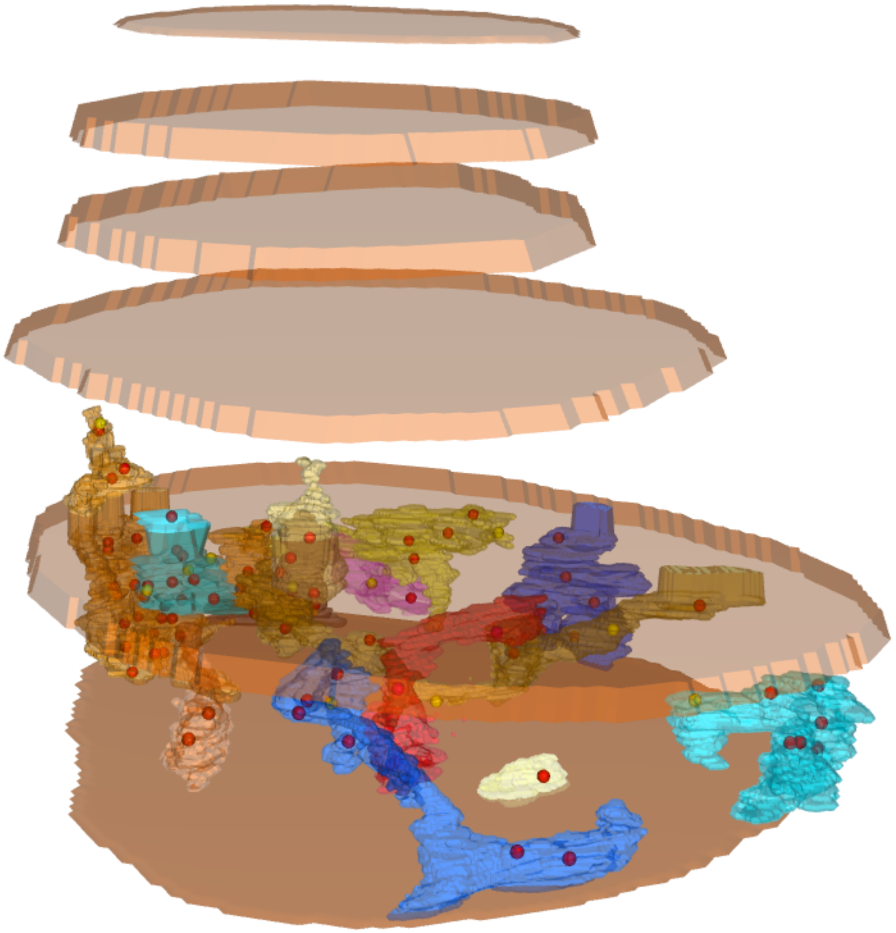

Table S4-1 VA7-OSN synaptic inventory

	OSN	vol (µm^3)	surf (µm^2)	pre	post	total	ratio	sy/ µm3	pre	post	sy/ µm2	pre	post	id
1	OSN1a-m	1.23	12.58	5	1	6	5.0	4.88	4.07	0.81	0.48	0.40	0.08	1393
2	OSN1b	1.08	7.79	3	0	3		2.78	2.78	0.00	0.39	0.39	0.00	1097
3	OSN1c	5.84	52.53	26	2	28	13.0	4.79	4.45	0.34	0.53	0.49	0.04	628
4	OSN2m	1.37	12.87	1	1	2	1.0	1.46	0.73	0.73	0.16	0.08	0.08	665
5	OSN3a	1.92	19.88	6	1	7	6.0	3.65	3.13	0.52	0.35	0.30	0.05	530
7	OSN4	2.03	18.55	4	1	5	4.0	2.46	1.97	0.49	0.27	0.22	0.05	663
1	OSN11	2.90	26.61	6	3	9	2.0	3.10	2.07	1.03	0.34	0.23	0.11	1079
9	OSN13	0.23	3.22	1	0	1		4.35	4.35		0.31	0.31		1429
10	OSN14	1.19	13.00	4	0	4		3.36	3.36		0.31	0.31		1442
11	OSN16	0.85	8.34	2	0	2		2.35	2.35		0.24	0.24		1512
12	OSN17	2.43	23.22	6	1	7	6.0	2.88	2.47	0.41	0.30	0.26	0.04	1528
13	OSN18	1.68	17.08	2	2	4	1.0	2.38	1.19	1.19	0.23	0.12	0.12	1811
14	OSN19	1.32	14.19	6	1	7	6.0	5.30	4.55	0.76	0.49	0.42	0.07	1932
15	OSN21	1.01	10.43	8	2	10	4.0	9.90	7.92	1.98	0.96	0.77	0.19	2432
	total	25.08	240.29	80	15	95	5.3	3.79	3.19	0.60	0.40	0.33	0.06	

vol: neurite volume; surf: neurite surface; total: number of all synapses counted per profile; pre: presynaptic site (output synapse) post: postsynaptic site (input synapse); ratio: number of out-to-input synapses; sy: synapse; sy/µm^3: volumetric density ;sy/µm^2: surface density

Table S4-2 VA7-OSN synaptic configuration

config	OSN1a-m	..1b	..1c	..2m	..3a	..4	..1	..3	..4	..16	..17	..18	..19	..21		all PN	percent	sum targets	percent
	pre	pre	pre	pre	pre	pre	pre	pre	pre	pre	pre	pre	pre	pre		.			
3	1		3													4	5.6%	12	2.62%
4	1		9		1	1			1		1		2	3		19	26.4%	76	16.59%
5			4		1	2	1				2			2		12	16.7%	60	13.10%
6		1	4		1		1						1	1		9	12.5%	54	11.79%
7	1	1	4				1		1	1			2	1		12	16.7%	84	18.34%
8								1	1	1						3	4.2%	24	5.24%
9			1		1		1				1			1		5	6.9%	45	9.83%
10		1										1				2	2.8%	20	4.37%
11							1						1			2	2.8%	22	4.80%
13																0	0.0%	0	0.00%
14									1							1	1.4%	14	3.06%
15						1										1	1.4%	15	3.28%
20							1									1	1.4%	20	4.37%
															**total**	**72**	**100 %**	**458**	**100 %**
															< 7	44	61.1%	202	44.10%
															> 6	28	38.9%	256	55.90%
config	OSN1a-m	..1b	..1c	..2m	..3a	..4	..1	..3	..4	..16	..17	..18	..19	..21		all PN	percent	sum targets	percent
	post	post	post	post	post	post	post	post	post	post	post	post	post	post					
4				1	1		2									4	30.8%	16	18.82%
5			1			1										2	15.4%	10	11.76%
6			1		1		1				1		1			5	38.5%	30	35.29%
9					1											1	7.7%	9	10.59%
20													1			1	7.7%	20	23.53%
															**total**	**13**	**100 %**	**85**	**100 %**
															< 7	11	84.6%	56	65.9%
															> 6	2	15.4%	114	34.1%

config: synaptic configuration, e.g. 4 = tetrad, **total:** number of configurations; **sum targets**: number of all postsynaptic profiles targeted by output synapses
