# Supplementary material for: Synaptic circuitry of identified neurons in the antennal lobe of Drosophila melanogaster
Source: J Comp Neurol. 2016 Mar 9;524(9):1920–56. doi: 10.1002/cne.23966 (PMC6680330; doi:10.1002/cne.23966)
Supplement: Supplementary file 5 — Supporting Information Figure 5. [file CNE-524-1920-s005.pdf]

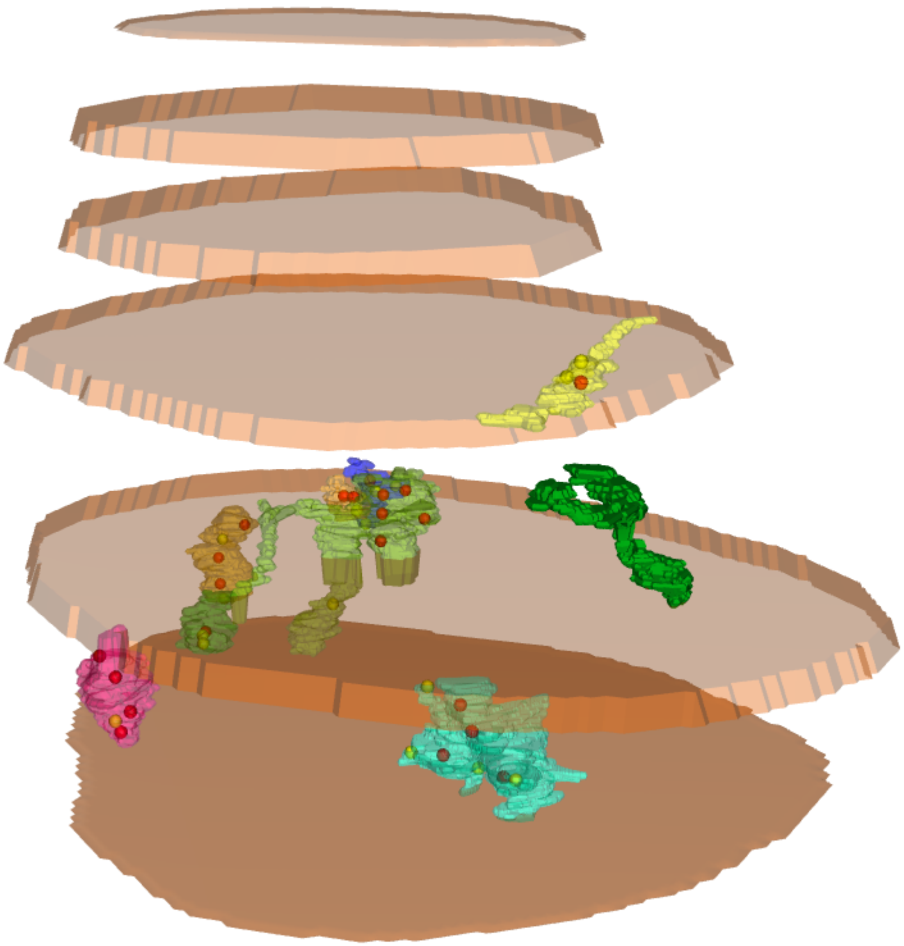

**Table S5-1 VA7-LN synaptic inventory**

| | LN | vol ($\mu m^3$) | surf ($\mu m^2$) | pre | post | total | ratio | sy/$\mu m^3$ | pre | post | sy/$\mu m^2$ | pre | post | id |
|---|---|---|---|---|---|---|---|---|---|---|---|---|---|---|
| 1 | LN1 | 1.32 | 14.34 | 5 | 1 | 6 | 5.0 | 4.55 | 3.79 | 0.76 | 0.42 | 0.35 | 0.07 | 2495 |
| 2 | LN2 | 0.79 | 6.42 | 4 | 1 | 5 | 4.0 | 6.33 | 5.06 | 1.27 | 0.78 | 0.62 | 0.16 | 2621 |
| 3 | LN3 | 0.25 | 3.8 | 1 | 2 | 3 | 0.5 | 12.00 | 4.00 | 8.00 | 0.79 | 0.26 | 0.53 | 2633 |
| 4 | LN4 | 0.12 | 1.96 | 1 | 3 | 4 | 0.3 | 33.33 | 8.33 | 25.00 | 2.04 | 0.51 | 1.53 | 2704 |
| 5 | LN5 | 0.12 | 1.62 | 2 | 1 | 3 | 2.0 | 25.00 | 16.67 | 8.33 | 1.85 | 1.23 | 0.62 | 2688 |
| 6 | LN6 | 0.66 | 8.93 | 2 | 3 | 5 | 0.7 | 7.58 | 3.03 | 4.55 | 0.56 | 0.22 | 0.34 | 2737 |
| 7 | LN7 | 1.55 | 13.53 | 4 | 4 | 8 | 1.0 | 5.16 | 2.58 | 2.58 | 0.59 | 0.30 | 0.30 | 2747 |
| 8 | LN8 | 0.4 | 3.58 | 1 | 3 | 4 | 0.3 | 10.00 | 2.50 | 7.50 | 1.12 | 0.28 | 0.84 | 2761 |
| 9 | LN9 | 0.58 | 5.56 | 3 | 2 | 5 | 1.5 | 8.62 | 5.17 | 3.45 | 0.90 | 0.54 | 0.36 | 3526 |
| | total | 5.79 | 59.74 | 23 | 20 | 43 | 1.2 | 7.43 | 3.97 | 3.45 | 0.72 | 0.39 | 0.33 | |

vol: neurite volume; surf: neurite surface; total: number of all synapses counted per profile; pre: presynaptic site (output synapse) post: postsynaptic site (input synapse); ratio: number of out-to-input synapses; sy: synapse; sy/$\mu m^3$: volumetric density ;sy/$\mu m^2$: surface density

## Table S5-2 VA7-LN synaptic configuration

| configuration | LN1 | LN2 | LN3 | LN4 | LN5 | LN6 | LN7 | LN8 | LN9 | | all PN | percent | sum targets | percent |
|---|---|---|---|---|---|---|---|---|---|---|---|---|---|---|
| | pre | pre | pre | pre | pre | pre | pre | pre | pre | | | | | |
| | | | | | | | | | | | | | | |
| 3 | 3 | 1 | 1 | | 2 | 1 | | | 2 | | 10 | 43.5% | 30 | 36.6% |
| 4 | 2 | 3 | | 1 | | 1 | 4 | 1 | 1 | | 13 | 56.5% | 52 | 63.4% |
| | | | | | | | | | | **total** | **23** | **100.0%** | **82** | **100.0%** |
| | | | | | | | | | | | | | | |
| configuration | LN1 | LN2 | LN3 | LN4 | LN5 | LN6 | LN7 | LN8 | LN9 | | **sum** | percent | **sum** | percent |
| | post | post | post | post | post | post | post | post | post | | | | | |
| | | | | | | | | | | | | | | |
| 3 | | | 1 | | | | | | | | 1 | 5.3% | 3 | 3.3% |
| 4 | 1 | | 1 | 2 | 1 | 3 | 2 | 2 | 1 | | 13 | 68.4% | 52 | 57.1% |
| 5 | | | | 1 | | | | 1 | | | 2 | 10.5% | 10 | 11.0% |
| 6 | | | | | | | | | 1 | | 1 | 5.3% | 6 | 6.6% |
| 8 | | 1 | | | | | | | | | 1 | 5.3% | 8 | 8.8% |
| 12 | | | | | | | 1 | | | | 1 | 5.3% | 12 | 13.2% |
| | | | | | | | | | | **total** | **19** | **100.0%** | **91** | **100.0%** |
| | | | | | | | | | | **< 7** | **17** | **89.5%** | **71** | **78.0%** |
| | | | | | | | | | | **> 6** | **2** | **10.5%** | **20** | **22.0%** |

**config:** synaptic configuration, e.g. 4 = tetrad, **total:** number of configurations; **sum targets**: number of all postsynaptic profiles targeted by output synapses
