# Supplementary material for: Synaptic circuitry of identified neurons in the antennal lobe of Drosophila melanogaster
Source: J Comp Neurol. 2016 Mar 9;524(9):1920–56. doi: 10.1002/cne.23966 (PMC6680330; doi:10.1002/cne.23966)
Supplement: Supplementary file 6 — Supporting Information Figure 6. [file CNE-524-1920-s006.pdf]

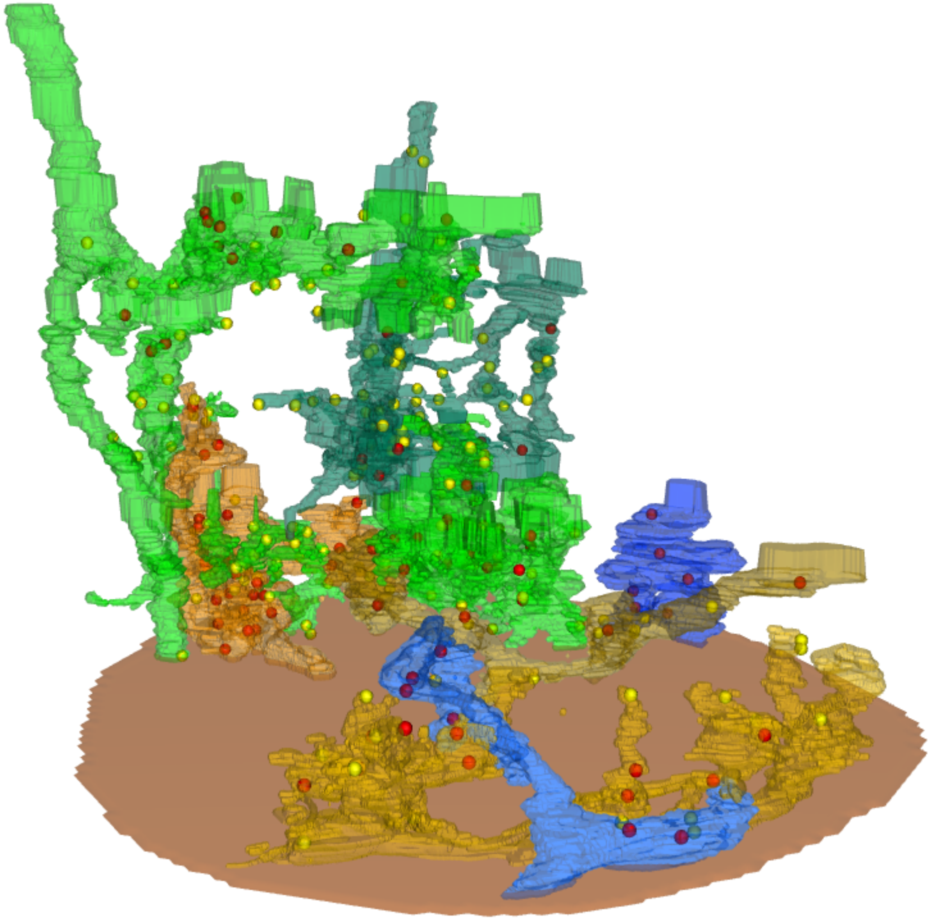

Table S6-1 VA7: OSN – PN – LN connectivity

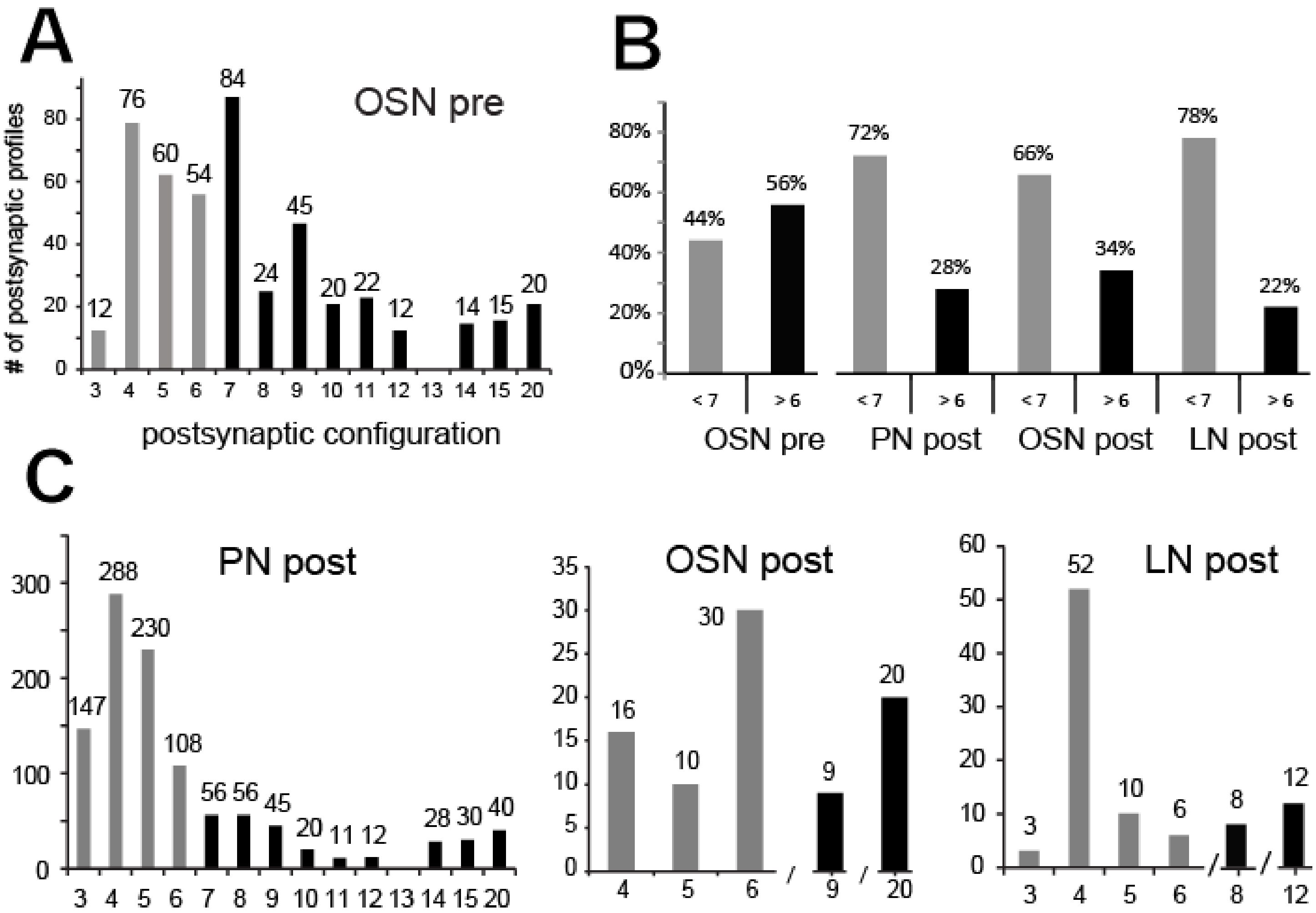

pre: presynaptic; **post**: postsynaptic; **PN**: projection neuron; **OSN**: putative olfactory sensory neuron; **LN**: putative local interneuron

Table S6-2 Connectivity of VA7 cell types

pre	connector	target	input to			
			N	PN	OSN	LN
PN4	30	125	99	21	4	1
			79.20%	16.80%	3.20%	0.80%
OSN	73	465	152	308	2	2
			32.7%	66.2%	0.4%	0.4%
LN	23	81	43	32	3	3
			53.1%	39.5%	3.7%	3.7%
post			input from			
			N	PN	OSN	LN
PN4	80	390	351	9	26	3
			90.00%	2.31%	6.67%	0.77%
OSN	9	59	53	3	3	0
			89.8%	5.1%	5.1%	0.0%
LN	18	87	72	9	3	3
			82.8%	10.3%	3.4%	3.4%

pre: presynaptic; **post**: postsynaptic; **target**: number of postsynaptic profiles targeted by a pre-synapse; **N**: non-identified profile **PN**: projection neuron; **OSN**: putative olfactory sensory neuron; **LN**: putative local interneuron
