# Supplementary material for: Synaptic circuitry of identified neurons in the antennal lobe of Drosophila melanogaster
Source: J Comp Neurol. 2016 Mar 9;524(9):1920–56. doi: 10.1002/cne.23966 (PMC6680330; doi:10.1002/cne.23966)
Supplement: Supplementary file 7 — Supporting Information Figure 7. [file CNE-524-1920-s007.pdf]

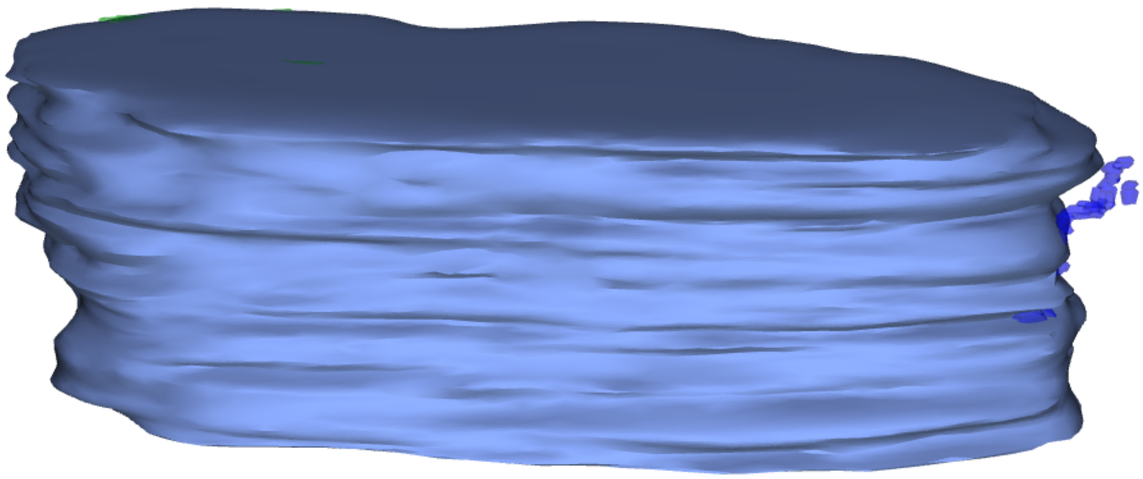

Table S7-1 DL5 synaptic inventory

	PN	vol (µm³)	surf (µm²)	pre	post	total	ratio	sy/µm³	pre	post	sy/µm²	pre	post	id
1	PN1	34.3	252.21	47	121	168	0.39	4.90	1.37	3.53	0.67	0.19	0.48	569
2	PN2	14.88	137.46	21	67	88	0.31	5.91	1.41	4.50	0.64	0.15	0.49	749
	**all PN**	**49.18**	**389.67**	**68**	**188**	**256**	**0.36**	**5.21**	**1.38**	**3.82**	**0.66**	**0.17**	**0.48**	
	**OSN**	vol (µm³)	surf (µm²)	pre	post	sum	ratio	sy/µm³	pre	post	sy/µm²	pre	post	id
1	ON1	1.55	19.44	3		3		1.94	1.94	0.00	0.15	0.15	0.00	846
2	OSN2	1.13	11.92	7	1	8	7.00	7.08	6.19	0.88	0.67	0.59	0.08	1533
	**all OSN**	**2.68**	**31.36**	**10**	**1**	**11**	**10.00**	**4.10**	**3.73**	**0.37**	**0.35**	**0.32**	**0.03**	

vol: neurite volume; surf: neurite surface; total: number of all synapses counted per profile; pre: presynaptic site (output synapse) post: postsynaptic site (input synapse); ratio: number of out-to-input synapses; sy: synapse; sy/µm³: volumetric density ; sy/µm²: surface density

Table S7-2 DL5-PN synaptic configuration

config	PN1	PN2		all PN	percent	sum targets	percent
	pre	pre					
3	14	4		18	27.3%	54	18.9%
4	11	11		22	33.3%	88	30.9%
5	14	3		17	25.8%	85	29.8%
6	4	1		5	7.6%	30	10.5%
7	2	2		4	6.1%	28	9.8%
**total**	**45**	**21**		**66**	**100.0%**	**285**	**100.0%**
config	PN31	PN32					
	post	post					
2	13			13	8.2%	26	3.6%
3	28			28	17.6%	84	11.6%
4	34	6		40	25.2%	160	22.1%
5	25	20		45	28.3%	225	31.1%
6	11	7		18	11.3%	108	14.9%
7	4	2		6	3.8%	42	5.8%
8	3	2		5	3.1%	40	5.5%
9	1	2		3	1.9%	27	3.7%
10							
11		1		1	0.6%	11	1.5%
**total**	**119**	**40**		**159**	**100.0%**	**723**	**100.0%**
			**<7**	**144**	**90.6%**	**603**	**83.4%**
			**>6**	**15**	**9.4%**	**120**	**16.6%**

config: synaptic configuration, e.g. 4 = tetrad, **total:** number of configurations; **sum targets**: number of all postsynaptic profiles targeted by output synapses
