# Supplementary material for: Synaptic circuitry of identified neurons in the antennal lobe of Drosophila melanogaster
Source: J Comp Neurol. 2016 Mar 9;524(9):1920–56. doi: 10.1002/cne.23966 (PMC6680330; doi:10.1002/cne.23966)
Supplement: Supplementary file 8 — Supporting Information Figure 8. [file CNE-524-1920-s008.pdf]

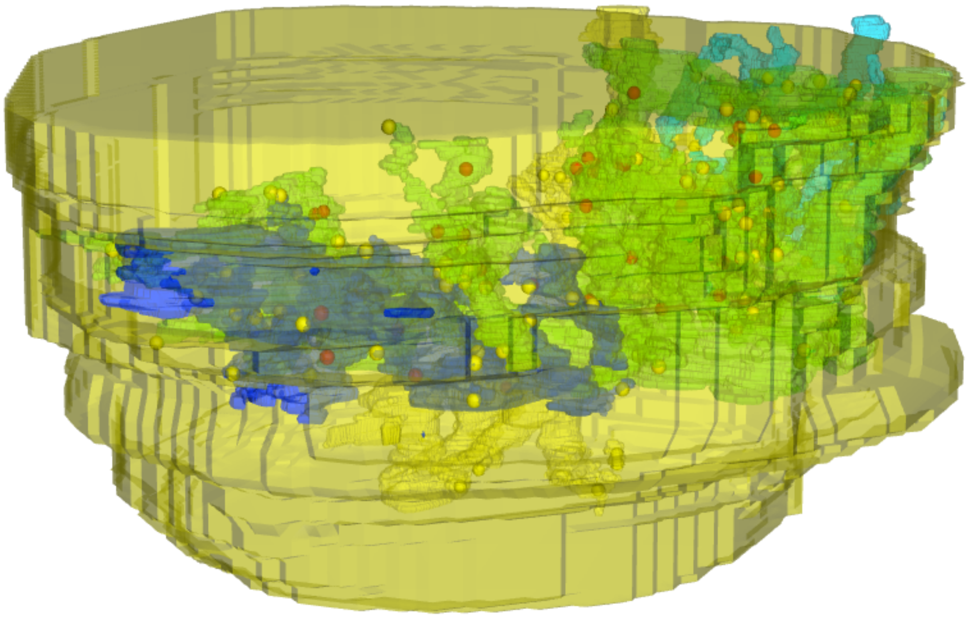

Table S8-1 DM2 synaptic inventory

		vol (µm³)	surf (µm²)	pre	post	total	ratio	sy/µm³	pre	post	sy/µm²	pre	post	id
PN														
1	PN10	4.43	36.29	2	6	7	0.40	1.58	0.45	1.13	0.19	0.06	0.14	638
2	PN31	4.25	43.7	2	23	25	0.09	5.88	0.47	5.41	0.57	0.05	0.53	711
3	PN32	4.87	49.37	1	18	19	0.06	3.90	0.21	3.70	0.38	0.02	0.36	687
4	PN33	26.31	220.97	28	63	91	0.44	3.46	1.06	2.39	0.41	0.13	0.29	689
5	PN47	1.45	16.22		6	6		4.14	0.00	4.14	0.37	0.00	0.37	719
6	PN50	6.48	56.75	4	23	27	0.17	4.17	0.62	3.55	0.48	0.07	0.41	2406
	**all PN**	**47.79**	**423.3**	**37**	**139**	**176**	**0.27**	**3.68**	**0.77**	**2.91**	**0.42**	**0.09**	**0.33**	
OSN														
2	OSN2	1.65	17.97	2	-	2		1.21	1.21	0.00	0.11	0.11	0.00	1479
3	OSN3	0.87	8.48	2	1	3	2.00	3.45	2.30	1.15	0.35	0.24	0.12	2592
2	OSN4	1.54	14.66	3	3	6	1.00	3.90	1.95	1.95	0.41	0.20	0.20	2595
33	OSN34	1	10.51	2	4	6	0.50	6.00	2.00	4.00	0.57	0.19	0.38	691
	**all OSN**	**5.15**	**51.62**	**9**	**8**	**17**	**1.13**	**3.30**	**1.75**	**1.55**	**0.33**	**0.17**	**0.15**	

vol: neurite volume; surf: neurite surface; total: number of all synapses counted per profile; pre: presynaptic site (output synapse) post: postsynaptic site (input synapse); ratio: number of out-to-input synapses; sy: synapse; sy/µm³: volumetric density ;sy/µm²: surface density

Table S8-2 DM2 synaptic configuration

config	PN31	PN32	PN33	PN50		all PN	percent	sum targets	percent
	pre	pre	pre	pre					
2			4			4	12.1%	8	6.6%
3	1	1	7			9	27.3%	27	22.3%
4	1		14	2		17	51.5%	68	56.2%
5			1			1	3.0%	5	4.1%
6			1			1	3.0%	6	5.0%
7			1			1	3.0%	7	5.8%
**total**	**2**	**1**	**28**	**2**		**33**	**100.0%**	**121**	**100.0%**
config	PN31	PN32	PN33	PN50					
	post	post	post	post					
2			7			7	5.7%	14	2.8%
3	4	3	19	10		36	29.3%	108	21.4%
4	10	9	19	6		44	35.8%	176	34.9%
5	5	4	11	2		22	17.9%	110	21.8%
6	4	2	2	1		9	7.3%	54	10.7%
7			1			1	0.8%	7	1.4%
8				2		2	1.6%	16	3.2%
9						0	0.0%	0	0.0%
10			2			2	1.6%	20	4.0%
**total**	**23**	**18**	**61**	**21**		**123**	**100.0%**	**505**	**100.0%**
					**< 7**	**118**	**95.9%**	**462**	**91.5%**
					**> 6**	**5**	**4.1%**	**43**	**8.5%**

config: synaptic configuration, e.g. 4 = tetrad, **total:** number of configurations; **sum targets**: number of all postsynaptic profiles targeted by output synapses
